# Supplementary material for: Extensive public health initiatives drive the elimination of Aedes aegypti (Diptera, Culicidae) from a town in regional Queensland: A case study from Gin Gin, Australia
Source: PLoS Negl Trop Dis. 2022 Apr 8;16(4):e0010243. doi: 10.1371/journal.pntd.0010243 (PMC9020727; doi:10.1371/journal.pntd.0010243)
Supplement: S1 Text — Fig A. Cycle of dengue transmission Fig D. Map of Gin Gin showing properties surveyed (yellow) and properties positive for Ae. aegypti (red). Table A. Dengue notifications by place of acquisition for Queensland (2001–2011) Table B. Dengue notifications by place of acquisition for Bundaberg LGA (2001–2011) Table C. Categories of Barker Hudson et al. (1988) including examples Table D. Mosquito species found in container habitats and positive premises. Table E. Prevalence of water bearing containers and number positive for Ae. aegypti. (PDF) [file pntd.0010243.s001.pdf]

Queensland Health

Central Regional Services

Population suppression of *Aedes aegypti* in Gin Gin, Bundaberg, Queensland

February to May 2012

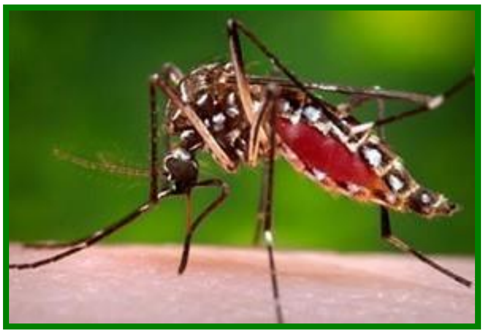

Report on population suppression efforts of *Aedes aegypti* in Gin Gin, Bundaberg Regional Council
February to May 2012

Published by the State of Queensland (Queensland Health), August 2012

This document is licensed under a Creative Commons Attribution 3.0 Australia licence. To view a copy of this licence, visit creativecommons.org/licenses/by/3.0/au

State of Queensland (Queensland Health) 2012

You are free to copy, communicate and adapt the work, as long as you attribute the State of Queensland (Queensland Health).

For more information contact:
Brisbane North Public Health Unit
Central Regional Services
Division of the Chief Health Officer
Queensland Health
PO Box 4256, Locked Bag 2
Stafford DC, Queensland Australia 4053.
(07) 3624 1111 fax
(07) 5562 1649

Disclaimer

Whilst considerable care has been taken in compiling this document, Queensland Health accepts no responsibility for errors or omissions, or for decisions or actions taken or not taken as a result of any information, statement or advice, express or implied, in this document.

Front cover image: Queensland Health 2011.

CONTENTS

EXECUTIVE SUMMARY **1**

INTRODUCTION **2**

WHAT IS DENGUE? 3

How does Dengue spread 3
Dengue in Queensland 3
Imported cases in Queensland 4
Dengue outbreaks in Queensland 4
Dengue mosquito vectors 5
Stakeholders and their roles 6

Background 7

Aim 7

Methods 7
STAFF 7
LARVAL SURVEILLANCE 8
LARVAL CONTROL 10
ADULT SURVEILLANCE 10
ADULT CONTROL 11
Public awareness and community engagement 11

Results 12
LARVAL SURVEILLANCE 12
ADULT SURVEILLANCE 14 **Conclusions and Recommendations 15**

References 16

EXECUTIVE SUMMARY

Exhaustive larval and adult surveillance within the town of Gin Gin indicate that the dengue vector, *Ae. aegypti*, is not well established in the area. Of the 473 properties surveyed, *Ae. aegypti* was only present at 12 properties (11 positive during larval surveillance, one as a result of the ovitrap network). Coupled with the larval control conducted during the survey, the adult control conducted in the three months that followed, and the shift into winter, it is likely that *Ae. aegypti* has been successfully suppressed in Gin Gin. As a result, Gin Gin could currently be considered at 'low risk' of a dengue outbreak. Continued surveillance and control efforts, coupled with a strong community involvement are necessary to ensure this status remains and to prevent the range expansion of this species into the wider Bundaberg Regional Council area.

INTRODUCTION

What is dengue?

Dengue is an infection caused by one of four dengue viruses in the family Flaviviridae. Other diseases caused by flaviviruses include yellow fever, Japanese encephalitis and Murray Valley encephalitis. In terms of morbidity, mortality and economic costs, dengue is the most important mosquito-borne viral disease of humans. Dengue occurs in over 100 countries worldwide and is found primarily in urban settings in the tropics. Fifty million cases of dengue are reported around the world each year and over 2.5 billion people are at risk of infection.

There are four dengue virus serotypes (DENV- 1, 2, 3 and 4) and genetic variants of these serotypes are found in different geographic locations. Therefore a person can acquire a maximum of four dengue infections during their lifetime, one infection with each dengue serotype. Infection with one dengue serotype confers immunity to that particular serotype, but may result in an increase risk of complications if subsequent infections with other serotypes occur. Infection with the dengue virus may be subclinical (asymptomatic) or may cause illness ranging from a mild fever to a severe, even fatal, condition such as dengue haemorrhagic fever (DHF) or dengue shock syndrome (DSS). Hospitalisation may be required depending on the severity of symptoms. DHF manifests generally as plasma leakage leading to shock that can be fatal, particularly among young children. Approximately 2.5% of people affected with DHF die, although with experience in dealing with DHF and timely treatment this rate is often reduced to less than 1 per cent. There is no vaccine for dengue.

How does Dengue spread

A female dengue mosquito becomes infected with dengue when it bites a human who is viraemic with the dengue virus (i.e. there are enough dengue virus in the person's blood to infect a mosquito). Generally in 8 -12 days the infected mosquito is able in turn to transmit the virus to people. One dengue-infected female mosquito is capable of biting and infecting several people during one feeding session. Consequently mosquito control activities need to be initiated urgently to reduce the likelihood of transmission. A patient with dengue can transmit the virus to mosquitoes within three to four days of contracting dengue. Thus the cycle of transmission may take only 14 days (Fig A).

Dengue in Queensland

Dengue has historically been reported in the Northern Territory, New South Wales and Queensland, but locally acquired dengue has only been reported in north Queensland in recent decades.

Transmission of the virus is limited by the distribution of its vector, the mosquito *Aedes aegypti* in Queensland (Fig B - removed due to copyright). While dengue is not endemic in Queensland, *Ae. aegypti* is widespread throughout urban tropical north Queensland and has been detected in many towns in sub-tropical Queensland as far south as Goomeri near the coast and Roma inland. It was first detected within the Bundaberg region in the town of Gin Gin in 2011 as part of joint Queensland

Health and Bundaberg Regional Council (BRC) surveillance program. A 2006 survey failed to detect *Ae. aegypti* which means this was a potentially recent introduction into the area.

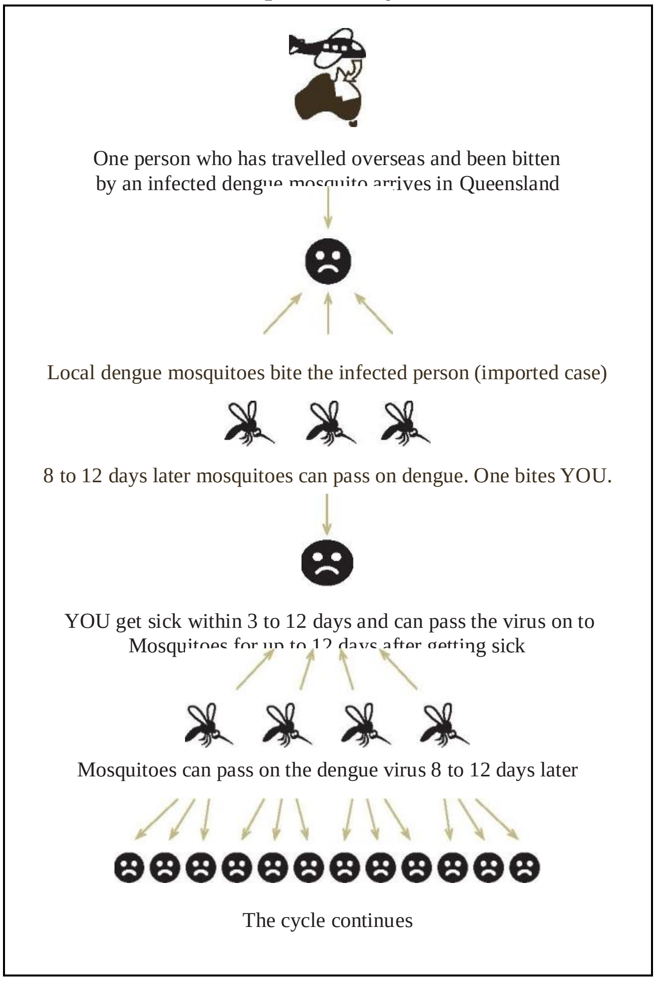

Fig A. Cycle of dengue transmission
Fig B. Removed due to copyright

Imported cases in Queensland

A single imported viraemic person with dengue in an area populated by the dengue vector and human hosts can lead to a dengue outbreak in Queensland. Dengue is not endemic to Australia and local dengue outbreaks, currently confined to north Queensland, all begin with a single imported case referred to as ‘patient zero’.

Queensland Health currently relies on surveillance by medical practitioners and diagnostic laboratories to detect imported cases. Since 1999 Queensland Health has been notified of an increasing number of imported cases per year. Currently most imported cases originate in Indonesia, Thailand, the Philippines and Papua New Guinea (PNG), largely due to increased dengue activity in these regions and the low cost of holidaying in Thailand and Indonesia.

Dengue outbreaks in Queensland

Queensland has a history of dengue epidemics dating back to 1879, most of which occurred in north Queensland. Thirteen notable dengue epidemics have occurred in Queensland since 1885. The first fatality attributed to dengue occurred in Charters Towers in 1885 and the first fatality

attributed to DHF occurred in the same town during the 1897 epidemic, when 60 fatalities were recorded (30 of those were children).

Based on the number of dengue notifications by place of acquisition for Queensland in the past 10 years, overseas acquired cases have become much more common in recent years (Table A). This is also evident in the Bundaberg region (Table B), which has seen 2 imported cases in both 2010 and 2011 whereas these events were previously very rare (a single importation in 2003).

Table A. Dengue notifications by place of acquisition for Queensland (2001 - 2011)

Year	Queensland acquired	Overseas acquired	Not stated	Total
2001	8	22	14	**44**
2002	29	48	2	**79**
2003	658	49	7	**714**
2004	240	42	3	**285**
2005	75	39	2	**116**
2006	38	39	4	**81**
2007	47	67	4	**118**
2008	88	95	4	**187**
2009	957	112	2	**1071**
2010	78	207	3	**288**
2011	71	113	0	**184**
**Grand Total**	**2289**	**833**	**45**	**3167**

Table B. Dengue notifications by place of acquisition for Bundaberg LGA (2001 - 2011)

Year	Queensland acquired	Overseas acquired	Not stated	Total
2001	0	0	0	**0**
2002	0	0	0	**0**
2003	1	0	0	**1**
2004	0	0	0	**0**
2005	0	0	0	**0**
2006	0	0	0	**0**
2007	0	0	0	**0**
2008	0	0	0	**0**
2009	0	0	0	**0**

2010	0	2	0	**2**
2011	0	2	0	**2**
**Grand Total**	**1**	**4**	**0**	**5**

Dengue mosquito vectors

In Queensland, the dengue virus is almost exclusively transmitted by the highly domesticated *Ae. aegypti* mosquito. *Aedes aegypti* is unusual in that it breeds primarily in domestic environments and does not often bite at night. An exotic species, *Ae. albopictus* (a less competent vector of dengue), is also a concern for colonising mainland Queensland and Australia due to its detection (May 2005) and rapid establishment in the Torres Strait, despite the efforts of a mosquito control program by Queensland Health and the Commonwealth Government to contain the species. This vector was detected on the northern most tip of Cape York in 2009. Intense eradication measures by Queensland Health and Local Government successfully prevented the establishment of *Ae. albopictus* in this location.

<u>Breeding and larval habitat</u>

Aedes aegypti breed primarily in artificial containers holding water, including cans, buckets, jars, pot plant dishes, birdbaths, boats, tyres and tarpaulins. With the recent emphasis placed on domestic water storage in Queensland, ad hoc water storage devices, poorly maintained roof gutters and rainwater tanks are important potential breeding sites. These mosquitoes can also breed in natural sites such as bromeliads and fallen palm fronds. Subterranean sites such as wells, telecommunication pits and drain sumps can also be important breeding sites, especially in drier conditions. In addition to artificial breeding sites *Ae. albopictus* also breeds in other natural environments such as tree holes.

<u>Adult mosquito behaviour</u>

Unlike most mosquitoes that prefer swamps and bushland, *Ae. aegypti* is truly domesticated and prefers to live in and around people's homes. Household residents can exert a much higher degree of control on exposure to this species because it does not disperse far from breeding sites (e.g. 100-200 m). *Aedes albopictus* on the other hand is more dispersive and tolerant of cooler climates. The adult *Ae. aegypti* mosquito likes to rest in dark places such as wardrobes and under beds. Females are very cautious when biting, being easily disturbed and prefer to bite humans during daylight hours.

Stakeholders and their roles

<u>Local Government</u>

Local governments are delegated with the responsibility of monitoring and enforcing the *Public Health Act 2005 and Public Health Regulation 2005* which relate to mosquitoes and mosquito breeding sites. Many local governments conduct scientifically based mosquito management programs based on Integrated Pest Management (IPM). These programs include

elements of chemical and biological control, habitat modification and public education. Other local governments rely on health education and/or limited treatment of known breeding sites to control mosquitoes. Local governments are ideally placed to carry out mosquito management within their own areas. In addition to having knowledge of local conditions conducive to mosquito breeding, many have access to resources or the ability to acquire resources for conducting mosquito control operations eg. Insecticide application equipment, appropriate vehicles and staff.

Queensland Health

Queensland Health is responsible for setting strategic direction and implementing actions for the prevention of and response to dengue outbreaks in Queensland. This includes:

- Investigating notifications of dengue virus infections;
- Monitoring incidences of dengue in Queensland;
- Leading dengue surveillance and emergency control activities in dengue receptive areas in Queensland;
- Coordinating, supporting and assisting local government with the implementation of surveillance and control activities for dengue mosquitos through a partnership arrangement;
- Leading public awareness activities to promote self-protective behaviours by the public, including reducing mosquito breeding places around the home and businesses;
- Monitoring the distribution of dengue vectors in Queensland;
- Supporting local government through the provision of specialised training in mosquito identification, surveillance and control methods, and medical entomology support;
- Development of relevant public health legislation and monitoring and supporting its administration.

Australian Quarantine and Inspection Service (AQIS)

AQIS is responsible for detection of exotic mosquitoes on behalf of the Department of Health and Ageing (DoHA) at international first ports of entry into Australia. AQIS is also responsible for maintaining an exotic mosquito exclusion zone of 400 m around first ports of entry.

BACKGROUND

Following the detection of *Ae. aegypti* within the Bundaberg region in the town of Gin Gin in 2011 a report was provided to BRC detailing recommended actions in the town to prevent the establishment and potential spread of *Ae. aegypti* in the region. These included;

- Eliminating *Ae. aegypti* as soon as possible from premises where they were collected so as to reduce the risk of dengue transmission.
- Surveying premises in which *Ae. aegypti* were found at least once a month for five months (November-March) to determine whether *Ae. aegypti* are still present.
- Surveying the premises adjacent to the ones where *Ae. aegypti* were found to ensure that they are also free of this mosquito species.

Following the larval surveys, BRC undertook routine adult surveillance at and around the premise where *Ae. aegypti* was collected. Ovitraps were set at 4 'high risk' locations (the original positive premise, the council depot, ambulance station and caravan park) between the 4th and 11th of May. *Aedes aegypti* were recorded at the original positive premise. A network of BG traps was set at several different locations weekly between the 4th of May and 25th of October. *Aedes aegypti* was recorded at 4 of the 11 locations at which traps were placed on at least one occasion.

Following consultation with the Advanced Medical Entomologist, Central Regional Services (CRS), it was agreed that a population suppression program be implemented.

AIM

The aims of the population suppression program were to;

- Determine the extent of *Ae. aegypti* infestation across the entire town,
- Suppress the population of *Ae. aegypti* within Gin Gin to densities that are below the threshold for local transmission should a dengue case be imported into the area,
- Suppress the population of *Ae. aegypti* within Gin Gin to densities that are less likely to allow movement into other areas of Bundaberg, and
- Train BRC staff in the methods required for *Ae. aegypti* control and the use of portable data entry devices to improve service delivery across the region.

METHODS

Staff

Bundaberg Regional Council was the lead agency for the population suppression program with field and technical support from Queensland Health. The initial larval surveillance and control was undertaken over a two week period from Monday 30th January to Friday 17th February 2012. Staff training in inspection methodology and techniques was undertaken prior to commencement and further reinforced in the field.

Local Government staff involved in the program included;

- David Zorzan, Senior Environmental Health Officer (SEHO), Bundaberg RC
- Prue Brinkley, Environmental Health Officer (EHO), Bundaberg Regional Council (BRC)
- Julie Barazza, EHO, Bundaberg RC
- Giselle Parsons, EHO, Bundaberg RC
- Russell Lyons, EHO, Bundaberg RC
- Adam Gardner, EHO, Bundaberg RC
- Karen Marais, EHO, Bundaberg RC

- Darryn Bebendorf, Environmental Health Technical Officers (EHTO), Bundaberg RC
- Mark Hillier, EHTO, Bundaberg RC
- Brad Peel, Manager Environmental Health (MEH), North Burnett RC
- Sue Paul, EHO, North Burnett RC

Queensland Health staff involved in the program included;

- Ted Aldred, Assistant Manager Environmental Health (MEH), Central Regional Services (CRS)
- Dr Tim Hurst, Advanced Medical Entomologist, CRS
- Wayne Ingall, Advanced Environmental Health Officer (AEHO), CRS
- Jarod Butler, SEHO, CRS
- Jim Edwards, SEHO, CRS
- Ty Jackson, EHO, CRS
- Jason Gilmore, EHO, CRS
- Pauline Dunn, EHO, CRS
- Matt Wessling, EHO, CRS

Larval surveillance

To ensure a greater chance of success, the aim was to survey (and control) mosquitoes in every one of the approximately 480 premises in the town of Gin Gin. Each team (of 2 to 3 people) were provided with a map of the area and designated areas for which to conduct surveillance and control activities. Verbal consent was obtained at each premise and if nobody was home the property was re-visited at a later date. In the third week of the program an ‘Authorised Inspection Program’ was implemented under the Local government Act allowing power of entry without consent. Each premises visited was characterised from the entry gate by the Premise Condition Index (PCI, from Tun-Lin *et al*. [1]). The PCI was designed to rank the likelihood that any given property will be breeding dengue mosquitoes (*Ae. aegypti*) based on the subjective ranking (scored 1-3) of three criteria; the amount of shade, the relative maintenance condition of the house, and the relative tidiness of the yard. The PCI predicts that shady, untidy and poorly maintained houses (scoring a maximum of 9 points) will be most likely to provide containers capable of breeding mosquitoes. Conversely, the PCI predicts that premises that are unshaded, well-maintained and have tidy yards (scoring a minimum of 3 points) will be unlikely to provide containers available for mosquito breeding.

All natural and artificial containers holding water were checked for the occurrence of mosquito larvae. A sample of 6 to 12 larvae was taken from each positive habitat and placed into ethanol for identification. Dry containers that could potentially hold water were recorded as potential breeding sites. All containers were categorised into groups of breeding sites based on the description provided by Barker-Hudson et al [2] (Table C) and the details entered into portable data entry device.

Table C. Categories of Barker Hudson et al. [2] including examples

**Category**	**Examples of containers included in category**
Garden Container	plant pots and saucers, striking pots, water features, birdbaths etc 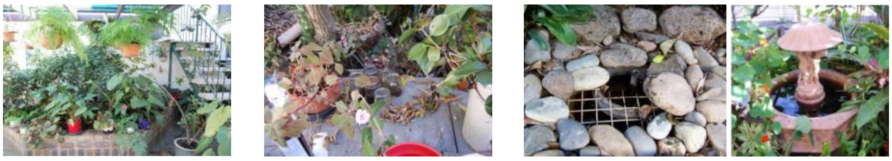
Natural Habitat	bromeliads, tree holds & plant axils tyres, disposable 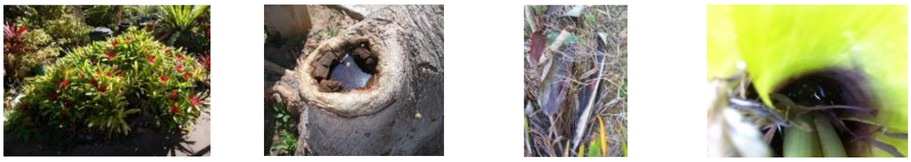
Rubbish	plastic containers, drink cans, old drums etc 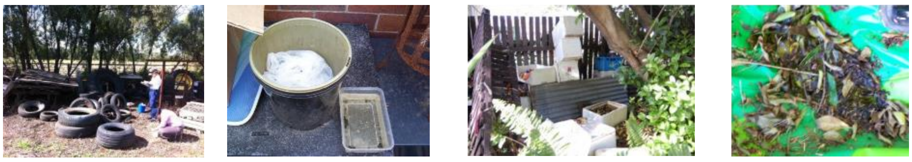
Discarded Household Items	retrievable or reusable garden containers, kitchen items etc 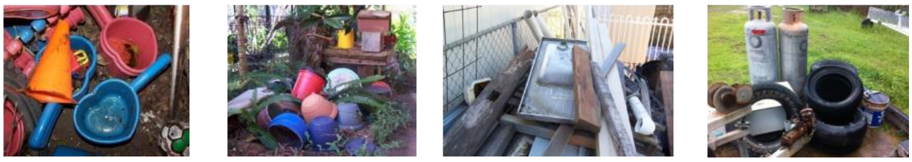
Water Storage Container	buckets, drums, containers designed to harvest and store water 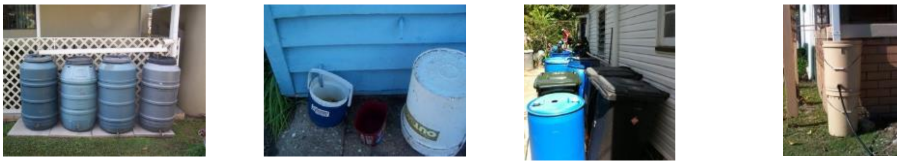
Building Fixtures	roof guttering, gully traps and sumps, fence posts etc 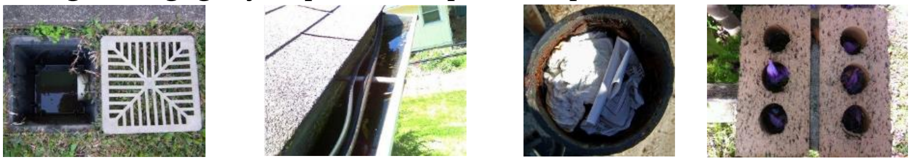
Recreational Items	boats, canoes, swimming pools 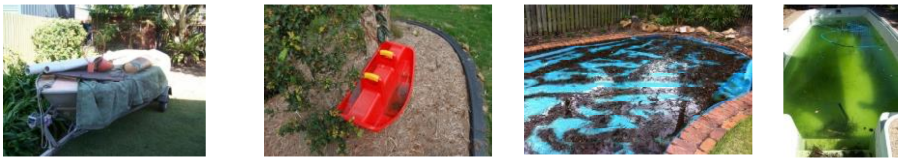

Domestic/Commercial Usage Containers	copper boilers, concrete wash tubs, tarpaulins, animal water dishes 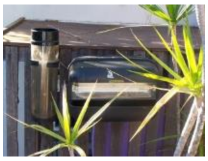 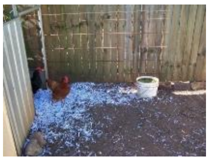 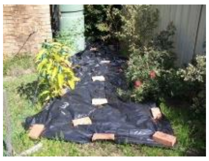 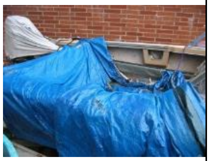
Rain water tanks	poly, galvanised etc 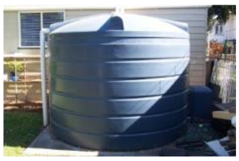 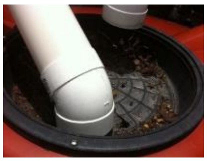 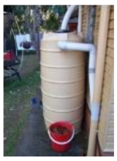 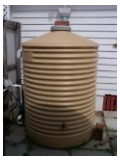 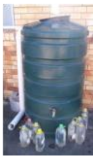

Fig C. Removed due to identifiable persons in image.

Peri-domestic mosquito breeding is quantified by using three indices:

1. The House Index (HI) is the percentage of premises where mosquito breeding occurs and is used to indicate the percentage of population at risk of dengue Fever.
2. The Container Index (CI) is the percentage of water holding containers in which mosquito breeding occurs.
3. The Breteau Index (BI) is number of containers where mosquito breeding occurs, per 100 houses and is used for estimating vector density.

According to WHO [3] *Ae. aegypti* density figures – (designed to evaluate public health significance) - a House Index or Breteau Index of more than five for *Ae. aegypti* indicates that there is risk of dengue virus transmission should the virus be present.

Larval control

Larval control consisted of the removal and/or insecticide treatment of containers that were breeding or could potentially breed *Ae. aegypti*. Prolink Pellets containing the insect growth regulator (S)-methoprene were put into hard-to-inspect containers or large containers that could not be emptied or removed (eg. drain sumps, drums, tyres, tree holes). Prolink ProSand was used in bromeliads. Prolink Pellets offer residual activity of one month duration due to the slow release formulation and a low non-target toxicity. Prolink XRBriquets, a residual slow release formulation block lasting approximately 3 months, were used in non-compliant (eg. damaged or removed screens) rainwater tanks to prevent emergence of adult mosquitoes. All chemical treatment was consistent with label recommendations, conducted by or supervised by a licensed Pest Management Technician (PMT) and Pest Control Advice (PCA) sheets provided when treatment occurred.

Adult surveillance

Adult surveillance was undertaken using a combination of ovitraps (Appendix 2) and BG traps (Appendix 3). Ovitraps were set weekly at 14 premises between the 21st of March and 22nd of May (10 trap weeks). BG traps were set fortnightly at 10 premises between the 22nd of March and the 14th of May (5 trap nights)

Adult control

Two (front and back) lethal ovitraps (Appendix 2) were set at each of the 11 positive premises. Lethal ovitraps are designed to kill ovipositing mosquitoes, thereby reducing populations of older mosquitoes. Lethal ovitraps have been successfully deployed in dengue control operations in north Queensland since 2004.

Public awareness and community engagement

The prevention of dengue is the responsibility of both government (state and local) and the public. Mosquito control workers cannot eliminate mosquito breeding in all homes and businesses in Queensland, hence an important element of dengue management is raising public awareness about the community's role in eliminating mosquito breeding at home and in the workplace as well as supporting positive behaviour change around personal protective practices. This was achieved through targeted awareness campaigns and community engagement strategies (Appendix 1) including;

- A 'Survey to eliminate *Aedes aegypti* to reduce a public health risk' fact sheet,
- A media release on the 'Gin Gin *Ae. aegypti* elimination program',
- Letter for premises that were positive for *Ae. aegypti*,
- Letter for premises within a 100m radius of premises that were positive for *Ae. aegypti*, and
- Provide an information sheet regarding prevention and control of mosquito breeding in the yard.

RESULTS

Larval surveillance

Surveillance and control activities were undertaken at a total of 473 premises (Fig D). A total of 5035 wet containers and 724 dry containers were found, resulting in an average of 12 potential breeding sites per premise. Mosquito breeding was identified in approximately 40% of these premises and 11% of wet containers (Table D). *Aedes aegypti* was recorded at approximately 2% of premises. The average PCI for positive premises was 5.0 ± 1.1. This is not significantly different to the average PCI for all properties in Gin Gin (4.9 ± 1.5) and reflects the good condition of the majority of properties. This demonstrates that it will be difficult to

effectively identify (and target) particular houses for surveillance in Gin Gin. The BI, HI and CI were 3.6, 2.3 and 0.3, respectively indicating the risk of dengue transmission at the time of the program was low. This also strengthens the theory that the introduction into the area was recent and that continued population suppression is possible.

Table D. Mosquito species found in container habitats and positive premises.

Species	No. of containers	No. of properties	% premises positive
Aedes notoscriptus	493	175	37.0
Culex quinquefasciatus	126	76	16.1
*Aedes aegypti**	17	11	2.3
Toxorhynchites speciosus	15	14	3.0
Lutzia halifaxii	6	6	1.3
Tripterpides punctolateralis	5	5	1.1
Culex australicus	2	2	0.4
Anopheles annuipes	2	2	0.4
Culex pullus	1	1	0.2
Unknown	1	1	0.2
ALL	566	190	100.0

*Appendix 4 lists the premises where *Ae. aegypti* were collected.

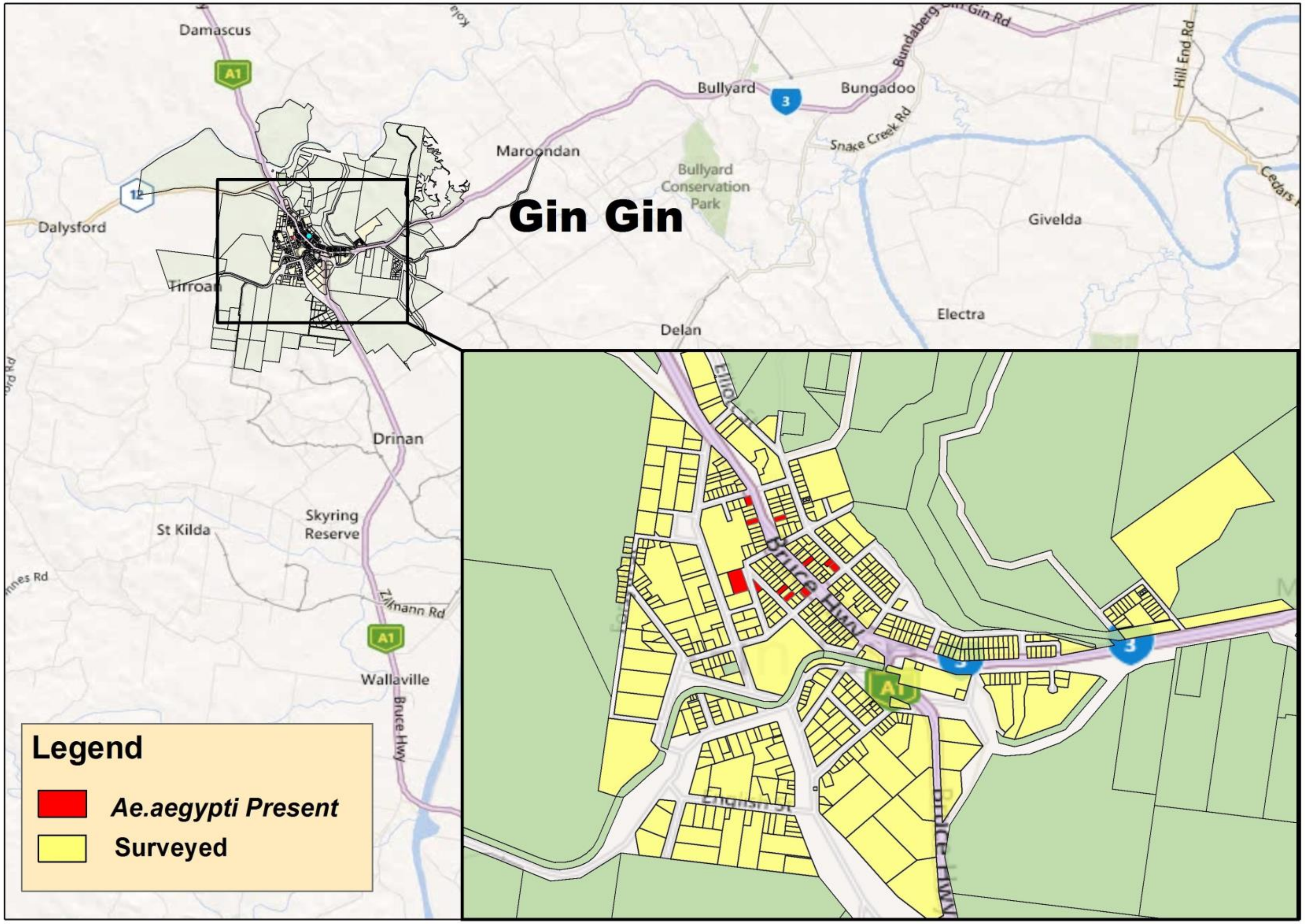

Fig D. Map of Gin Gin showing properties surveyed (yellow) and properties positive for *Ae. aegypti* (red). Map base layer from OpenStreet Map licensed under the Open Data Commons Open Database License (ODbL) by the OpenStreetMap Foundation (OSMF). Residential block imagery digitized manually from public domain cadastre data https://www.data.qld.gov.au/dataset/cadastral-data-queenslandseries.

The most prevalent potential mosquito breeding sites were natural habitats (39%) and garden containers (31%). The most prevalent container category positive for *Ae. aegypti* (35%) were garden accoutrements such as plant pots & saucers, birdbaths, buckets and striking pots (Table E). Discarded Household Items and domestic containers could also be considered a key container in Gin Gin backyards, that is, while only representing 13% of wet containers, these categories represented 47% of positive containers. The most common positive containers in this category were buckets. This information could be use in guiding community education campaigns and ensuring that householders are targeting the right breeding sites.

Table E. Prevalence of water bearing containers and number positive for *Ae. aegypti*.

Category	Total	Wet	Positive
Garden accoutrement	1796 (31.2)	1408 (28.0)	6 (35.3)
Discarded Household item	460 (8.0)	409 (8.1)	4 (23.5)
Domestic/Commercial use	259 (4.5)	246 (4.9)	4 (23.5)
Recreational Item	63 (1.1)	62 (1.2)	1 (5.9)
Water Storage	162 (2.8)	157 (3.1)	1 (5.9)
Rubbish	200 (3.5)	159 (3.2)	0
Building Fixture	224 (3.9)	205 (4.1)	0
Natural Habitat	2248 (39.0)	2044 (40.6)	0
TOTAL	5759	5035	17

There were 347 rainwater tanks (73.4% of premises inspected) recorded during the survey, almost all of these tanks were wet and 4 were positive for mosquito larvae (1 was positive for *Ae. aegypti* larvae). However, due to the difficulty of gaining access, not all tanks could properly be inspected or sampled and therefore more than 1 tank may of have been positive.

Adult surveillance

Over the 10 week ovitrapping period, *Ae. aegypti* were recorded at only one of the 14 ovitrap locations on only one occasion (week 7). Similarly, over the 10 week BG trapping period (5 fortnights), *Ae. aegypti* were recorded at only 2 locations at fortnight 1 and 1 location at fortnight 2. Only a single individual was collected on each occasion. These low numbers and low distribution are not unexpected given that the population suppression program was undertaken prior to this and that peak breeding season was over.

CONCLUSIONS AND RECOMMENDATIONS

Exhaustive larval and adult surveillance within the town of Gin Gin indicate that the dengue vector, *Ae. aegypti*, is not well established in the area. Of the 473 properties surveyed, *Ae. aegypti* was only present at 12 properties (11 positive during larval surveillance, one as a result of the ovitrap network). Coupled with the larval control conducted during the survey, the adult control conducted in the three months that followed, and the shift into winter, it is likely that *Ae. aegypti* has been successfully suppressed in Gin Gin. As a result, Gin Gin could be considered 'low risk' for a potential dengue outbreak. This will help guide the level of response required should an imported case be reported in the area in the immediate future.

Although this program was successful, it's continued success requires further surveillance and control efforts. BRC should consider an *Ae. aegypti* surveillance plan be introduced and be part of the Council's Mosquito Management Plan. Surveillance, prevention and control programs are essential for detecting and identifying the mosquito species populating an area, evaluating corresponding mosquito-borne disease risk and developing management plans to minimise the associated public health risk to communities. Specific recommendations as to future control and surveillance are as follows:

- Control (as undertaken in the current program) of mosquito breeding sites within a 100m radius of each positive premise during the next mosquito breeding season.
- Routine (weekly) surveillance of adults in areas within Gin Gin that are outside of the current distribution of *Ae. aegypti*. Given that PCI was not a reliable predictor of *Ae. aegypti* presence in this instance, 'key premises' should be selected based on the level of larval activity recorded during the current program.
- Given the potential for re-invasion from surrounding areas, thought should be given as to the potential mechanisms of introduction of *Ae. aegypti* into the area originally and for the potential movement of *Ae. aegypti* out of Gin Gin into surrounding towns and cities.
- An additional adult surveillance network (sticky traps are recommended) outside of Gin Gin should be established in key areas (see above) within the Bundaberg LGA for the early detection of *Ae. aegypti*.

Although the community was actively engaged during the current program and the right messages presented, it is difficult to gauge the impact of these messages on household behaviour in regards to removing potential mosquito breeding sites. Regardless, community participation is paramount to the success of any program based around the control of dengue vectors. As such, BRC should continue to support media campaigns aimed at increasing mosquito awareness within the community.

References

1. Tun-Lin, W, BH Kay and A Barnes 1995, The Premise Condition Index: A tool for streamlining surveys of *Aedes aegypti*, Am. J. Trop. Med. Hyg. 53: 591-594.

2. Barker-Hudson, P, Jones, R and Kay, BH 1988, Categorization of domestic breeding habitats of *Aedes aegypti* (Diptera: Culicidae) in northern Queensland, Australia. J Med. Entomol, 25: 178182.

3. World Health Organization (1972). An international system for the surveillance of vectors. *Wkly Epidem.Rec.* **47:** 178-182.

Appendix 1 – various community engagement strategies used during the program

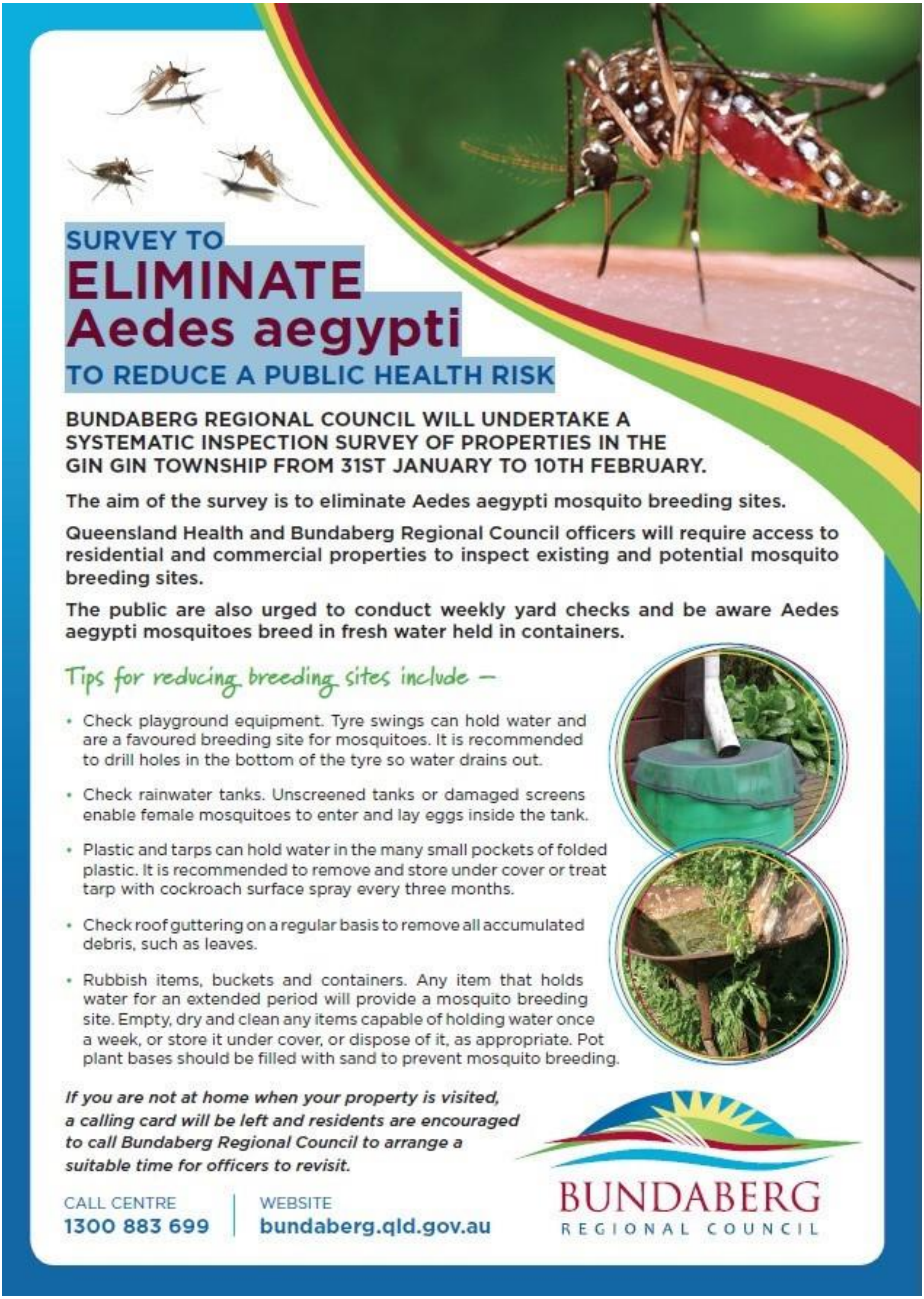

MEDIA RELEASE

BUNDABERG
REGIONAL COUNCIL

Gin Gin *Aedes Aegypti* elimination program

Council is continuing its efforts to eliminate the *Aedes aegypti* mosquito from the Gin Gin township area. Gin Gin residents have been extremely supportive during the program and in many cases breeding sites have been removed, but Council Officers have again recently found breeding in one of the mosquito monitoring sites that are collected weekly.

This indicates that the mosquito is still active in our region, even though the air temperatures have decreased. Council urges all Gin Gin residents to be diligent and take a look around their yard and work environment for containers that are either holding, or have the potential to, hold water. Where possible these containers should be emptied, and wiped out with a mild bleach solution or simply discarded of. For animal drinking containers, and other containers that cannot be removed residents should ensure that they are emptied and wiped out at least once a week and refilled with fresh, clean water. Residents are also asked to ensure that all rain water tanks are appropriately sealed to prevent mosquito access.

This particular mosquito has a very limited flight range and frequents backyards in search of containers holding water in and around the home, such as:

- cans
- buckets
- jars
- vases
- pot plant dishes
- birdbaths
- boats
- tyres – discarded with no rims
- blocked roof gutter
- striking containers (used to strike plants)
- tarpaulins and black plastic.

Bundaberg Regional Council's Health and Environment Spokesperson, Cr Wayne Honor, said the mosquito can also breed in natural containers like bromeliads and fallen palm fronds. "In drier conditions it also known to breed in water in subterranean sites such as wells, telecommunication pits, sump pits and gully traps" said Cr Honor. "Your vigilance will help stop the spread of this mosquito" added Cr Honor.

To find out more pick up a brochure from Council's Gin Gin Service Centre or Neighbourhood Centre or download from Council's website: http://bundaberg.qld.gov.au/feature/mosquito-survey-gin-gin

23.5.12

ALL MEDIA ENQUIRIES TO:
COUNCIL'S CORPORATE COMMUNICATIONS TEAM
on 1300 883 699 or communications@bundaberg.qld.gov.au

Elimination Program

BUNDABERG REGIONAL COUNCIL

Where to from here?

In order to eliminate the *Aedes Aegypti* mosquito from our region the following steps need to implemented:

- Eliminate or reduce as many available breeding sites (wet containers) and potential breeding sites (dry containers) as possible by turning them or removing containers from properties. We require your assistance in ensuring this is done on a continual basis throughout your property.

- Implement monitoring and control measures on properties that have been found to be breeding *Aedes aegypti* mosquito larvae. Council Officers will require your co-operation and permission to set up the following on your property:
    A. Adult mosquito monitoring equipment (BG Traps). The traps used are to capture live adult mosquitoes which are then taken back to a laboratory for identification. The traps utilised are either run by battery or electricity. These will be set up every two (2) weeks from the beginning of March and will be in place on your property for a minimum of a 24 hour period.
    B. "Lure n Kill" larvae traps. The trap is designed to attract female mosquitos searching for places to lay their eggs. The trap contains only a small amount of chemical that is not harmful to humans or animals. These traps are effective for a period of about six (6) weeks. Further details pertaining to these traps will be explained to you as the program progresses.

- Larvae monitoring stations will also be set in various properties located within 100m radius of properties that have been identified as breeding sites for the *Aedes aegypti* mosquito.

- Monitoring and trapping will be carried out every 2 weeks until <<Date>>; or until no *Aedes aegypti* mosquito are detected, whichever occurs first.

- A further full mosquito breeding survey of the entire town will be conducted <<Date>>.

If there are no further *Ae aegypti* mosquito found the program in <<area>> will have been successful and completed!!!

ABN 72 427 835 198
PO BOX 3130 BUNDABERG QUEENSLAND 4670
T: 1300 883 699 F: 07 4150 5410 E: ceo@bundaberg.qld.gov.au W: bundaberg.qld.gov.au

Date

[click here to insert name & address]

Dear <<Name>>

<u>Re: Mosquito Survey – (address)</u>

I refer to the survey recently conducted by officers from both <<Council>> and Queensland Health of the <<area>>, for the purposes of identifying any breeding areas of *Aedes aegypti* mosquito.

This survey has allowed us to identify breeding areas and to gain an understanding of the prevalence of this mosquito in the <<area>>. You are advised that *Aedes aegypti* mosquito larvae were found breeding on your property.

A control program is to be soon implemented by <<Council Name>> to reduce breeding areas and ultimately eliminate the *Aedes aegypti* mosquito from our region. To find out more about the A*edes aegypti* mosquito and how you may further assist, please read the attached fact sheets for more information.

If you require any further information, please contact <<Position>>, <<Officer Name>>, on <<Contact Number>>.

Yours sincerely

<u>Name</u> <u>Position</u>

Document No
File No

Dengue Mosquito Survey - Information for the householder/occupier

Although there is no immediate threat to you, the *Aedes aegypti* mosquito larvae was found on a property within 100 metres of your premises

Officers from Queensland Health and Local Government recently conducted a dengue mosquito survey in the <<area>> to check for the presence of the dengue transmitting mosquito *Aedes aegypti.*

What does this mean for you?

The mosquito found on the property is capable of spreading dengue if it bites a person who has dengue. Whilst there are no local cases of dengue in your area, please be aware that residents or tourists that have recently travelled to dengue affected areas and have been bitten by *Aedes aegypti* can increase the risk of local transmission.

Where do dengue mosquitoes breed? *Aedes aegypti* is a mosquito that lives close to humans and breeds in household containers that hold water including: pot plant bases buckets tyres pets water dishes tarpaulins and black plastic cans and plastic containers rainwater tanks with damaged or missing screens, birdbaths, boats and roof gutters.

Aedes aegypti does not breed in rivers, swamps, open drains, creeks or mangroves.

What should you do?
You should check in and around your home regularly and: throw out unused containers tip out containers that can hold water and store in a dry place regularly maintain the screens on rainwater tanks clean out roof gutters.

By taking these actions you can help reduce the risk of dengue transmission to your community.

How do dengue mosquitoes transmit dengue? Dengue mosquitoes are common in Queensland (though not as common in South East Queensland) but usually do not carry dengue. A female mosquito can only become infected with dengue after biting an infected human who has dengue. An infected person can transmit dengue to mosquitoes at home, at work or anywhere they visit. This can happen within 3-4 days of being bitten and can continue to do so up to 12 days.

Symptoms of dengue
Symptoms are most commonly seen in adults and older children. Young children may show no symptoms. Typical symptoms may include: sudden onset of fever (lasting three to seven days) intense headache (especially behind the eyes) muscle and joint pain (ankles, knees and elbows) unpleasant metallic taste in mouth, loss of appetite, vomiting, diarrhoea, abdominal pain flushed skin on face and neck, fine skin rash as fever subsides, rash on arms and legs, severe itching, peeling of skin and hair loss minor bleeding (nose or gums), heavy menstrual periods and extreme fatigue.

If you or someone you know has recently travelled to dengue infected areas and has symptoms of dengue please consult your general practitioner immediately.

Further information can be obtained by contacting <<Council Name & Section>> on <<Contact Number>> or by visiting the Queensland Health Dengue website www.health.qld.gov.au/dengue

<<Date>>

[click here to insert name & address]

Dear *[click HERE to insert Christian name where possible, not Sir/Madam]*

Re: Mosquito Survey – (address)

I refer to the survey recently conducted by officers from both Bundaberg Regional Council and Queensland Health of the <<area>>, for the purposes of identifying any breeding sites for the *Aedes aegypti* mosquito.

This survey has allowed us to identify breeding areas and to gain an understanding of the prevalence of this mosquito in the <<area>>. You are advised that *Aedes aegypti* mosquito larvae were found breeding at properties within a 100 metre radius of your property. With this in mind, we are requesting your assistance in allowing us to place monitoring equipment on your property.

The control program is to be soon implemented by <<Council>> to reduce breeding areas and ultimately eliminate the *Aedes aegypti* mosquito from our region. To find out more about the A*edes aegypti* mosquito and how you may further assist, please read the attached fact sheets for more information.

If you require any further information, please contact <<Position>>, <<Name>>, on <<Contact Number>>.

Yours sincerely

Name Position

File No

Appendix 2. OVITRAP PROCEDURES FOR *AE. AEGYPTI*

Dengue Action Response Team (DART) Tropical Population Health Network

Revised Aug 2008

EQUIPMENT

A. Trap Construction **B. Chemical Application**

- Anti-bird mesh • Bucket (10 L)
- Divot Buckets (1.2 L) • Bifenthrin insecticide (Country
- Material blade "Cutter" or scissors Bifenthrin Aqua)
- Glue panels • Container to store treated strips in
- Rubber bands -size 64 darkness (brown paper lunch bags)
- 'Giant' paper clips or 32mm 'fold • Disposable Gloves back' clips • Shade cloth
- Lucerne pellets • Drying rack
- Stanley Knife • Lucerne pellets
- Protective cover (butchers paper) • PPE for use with chemical
- Plastic 'cutting board' • Red flannelette (ovitrap cloth paddle)
- Silicon adhesive (Sika- Flex) • *s*-methoprene (Prolink pellets)
- Warning Labels

SUPPLIERS

• Ovitrap Buckets:

David Golf & Engineering Pty Ltd, Email: sales@davidgolf.com.au

Ph: 03 9540 0011 Fax: 03 9540 0909. 22 39-41 Winterton Road, Clayton, VIC 3168

Clayton Plastics, Email: clayplas@alphalink.com.au

Ph: 03 9480 0611 Fax: 03 9416 9984. 221-223 Dundas St, Preston, VIC 3072

Atlantic Paste & Glue Co, Email: mnentivegna@att.net

UVR-32 (Wet Glue)
Phone: (718) 4923648
Michael Bentivegna Jr Address: 170-53rd St Brooklyn, NY11232

$6.85US per glue strip minimum of 1000 strips needs to be purchased

- Glue Panels – TPHU Cairns
- Anti-bird mesh - Hardware store

- Materials to make drying rack- Hardware store
- Rubber bands, Stanley knife, cutting board etc. - stationery store
- Baby Oil – supermarket
- Lucerne pellets – stockfeed store
- Bifenthrin - **Globe Australia**, Utility Lane, Bohle, QLD, 4818. Ph 4774 8877, Fax 47748911
- *s*-methoprene pellets (Prolink) – **Pacific Biologics** - P/L PO Box 58 Scarborough Ph: 07 3283 5077 Fax: 07 3283 5088

1. CONSTRUCTION OF VARIOUS OVITRAPS

Cover a bench top with protective sheeting. Wear disposable gloves whenever appropriate.

A. Sticky Trap – for Adult Mosquitoes

1. Drill two overflow holes (6 mm diam.) 50mm from bucket brim and on opposite sides of bucket (to prevent glue strips being submerged in water).
2. Cut glue sheet into strips (DART prefer 5 cm W x 15 cm L) by starting from the edge of the glue sheet (where the glue is closest to the edge) and cutting at every 8th corrugation. We discard the narrower piece at the end (Fig.1A).
3. Cut each strip in half to complete a sticky trap panel (Fig. 1B). Two panels are used per bucket.

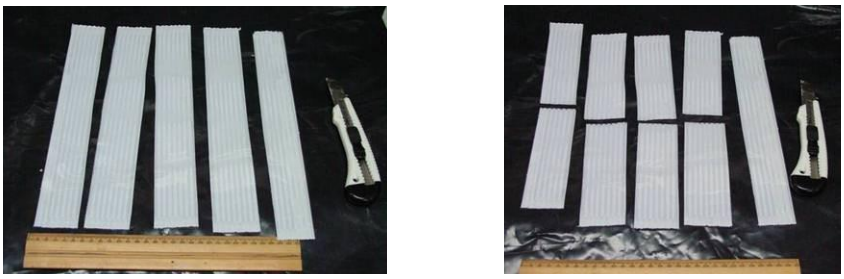

Fig. 1(A) Glue strips (approx. 5 cm W). **(B)** Glue strips cut into panels.

4. Attach panels to the bucket with a paper clip or a folding clip (Fig. 2). Paper clips need to be pressed down firmly to prevent the panel slipping.

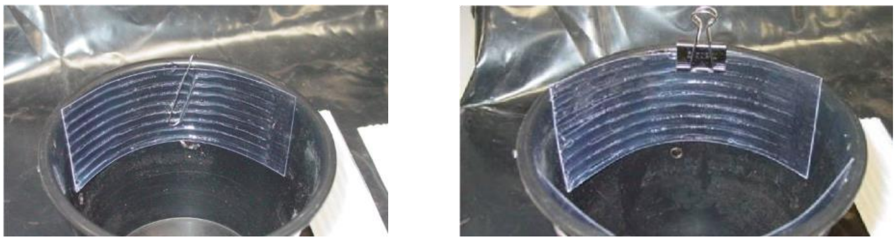

Fig. 2. Panels attached with a giant paper clip or fold back clip.

5. Fit mesh over top of bucket, using a rubber band to complete the Sticky Trap (Fig. 3).

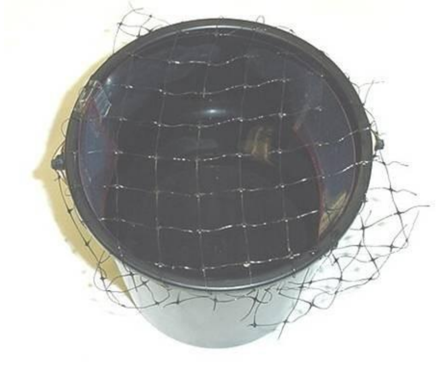

Fig. 3. Assembled Sticky Trap.

B. Clippy Trap – for Adult Mosquitoes

1. Cut a glue panel to size, using the DART template.

2. Drill an 80 mm hole in the base of one bucket (forms the top half of trap).
3. Some models of bucket will require the removal of a 'strut' under the lip (and on opposite sides) of top and bottom bucket (as per Fig. 4 LHS).

4. Roughen (with sandpaper) the first 10 mm from lip of the top bucket (end opposite to the 80 mm hole), i.e. roughen to the prefabricated line inside the bucket.

5. Apply 'Sika-Flex' carefully to the roughened section to form a neat ridge, level with the prefabricated line within the bucket. This ridge holds the glue panel in place. Ensure that the Sika-Flex ridge does not protrude beyond the prefabricated line.

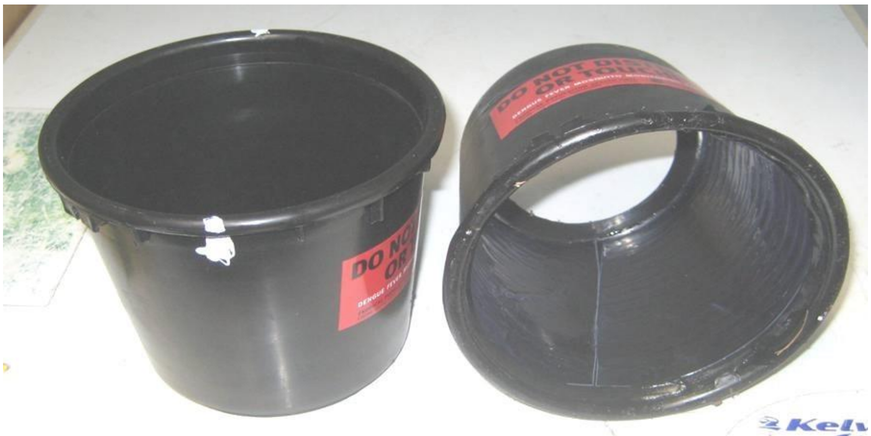

Fig. 4. The two halves of a Clippy Trap. Clip attachment points indicated by white marks (LHS bucket); glue panel held in place by Sika-Flex ridge (RHS bucket).

6. Apply 'Fold back' clips x 2 to clamp the buckets together (see Fig. 5A). The glue panel is replaced once the accumulation of insects interferes with the capture of mosquitoes (see Fig.5B).

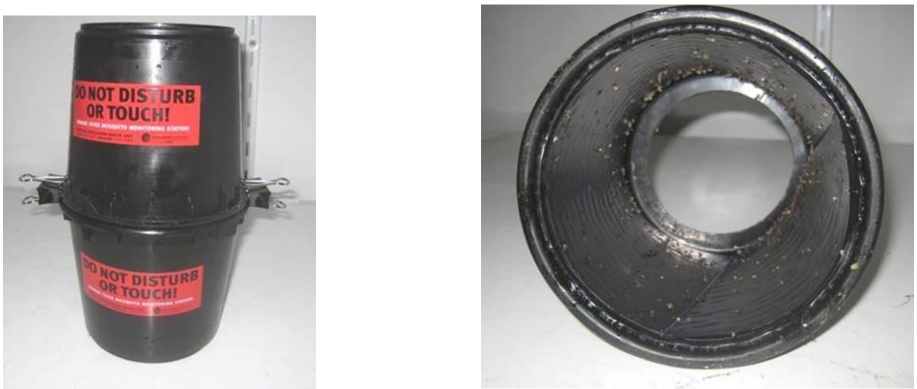

A. **B.**

Fig. 5 (A). Clippy Trap showing the position of the bulldog fold back clips. **(B)** Top half of Clippy Trap showing glue panel due for replacement.

C. Lethal Ovitrap.

1. Using 1L Bifenthrin bottle labelled as below

This bottle to be used for treating strips only.<br>Batch number ______<br>Manufacturing date ________<br>Country Bifenthrin Aqua

Mix chemical at initial mosquito control application rates into 10L plastic bucket.

2. Cut flannelette sheet to appropriate size.
   a. For small quantities of ovistrips - cut flannelette into strips (15 cm W x 200 cm L)
   b. For large quantities of ovistrips - cut flannelette sheet 1250mm. (see Appendix 1).
3. Add flannelette strips/sheet to bucket, completely submerge and soak (2 mins per strip).
4. Without wringing flannelette, spread strips/sheet on a plastic 'drying sheet' (or drying rack, see Appendix 2) where strips can dry undisturbed, out of sunlight.
5. Label Drying rack with info of each batch (Date of treatment, Batch No & Signed.)
6. Allow 24 hrs for flannelette to dry completely (Fig. 6). A fan can be used to speed up the drying process.

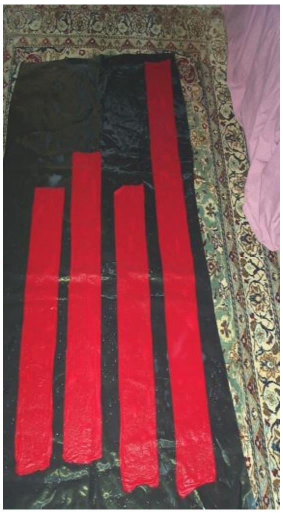

Fig. 6. Treated strips drying on plastic 'drying sheet' out of sunlight.

7. Cut flannelette strips to size (5 cm W x 15 cm L). For large quantities a 2-part jig should be considered (see Appendix 1).
8. Keep three random strips from each sheet of material for efficacy testing. Approximately nine strips per treated batch from drying rack. Store in Labelled plastic bag, (Batch No. & Date) then put in freezer in correct place.

9. Store 100 X treated strips in a container out of sunlight (eg. brown paper bag, cardboard box or in closed containers). With Batch Nos, Date of treatment and Your initials
10. Affix “warning label,”
11. Drill 2 x 8mm holes opposite each other in top just under rim (about 15mm down) of all black buckets.
12. Use gloves to affix one treated ovistrip per bucket with a giant paper clip/fold back clip.(use the treated strip bags with oldest dates first)
13. Use a rubber band to attach anti-bird mesh, to the top of the black Ovitrap.
14. Label each black carry box with one Batch No & date from when strips were treated. Only one Batch No per box. (this should be on brown paper bag strips came in)
15. Ovitraps should be deployed ASAP.
16. After eighteen months from treatment date randomly test three strips from each bag.
    Discard all strips after two years.

2. OVITRAP DEPLOYMENT

1. Set each ovitrap in an area protected from the weather (eg. under steps, house eaves, or under house), out of direct sunlight but visible to the mosquitoes.
2. Add appropriate level of tap water and add lucerne pellets (x 2, or @ 0.5 g). Sticky Traps and Clippy Traps are provided with a Prolink pellet (*s*-methoprene) to prevent mosquito breeding in traps (not necessary for Lethal Ovitraps).
3. Legislation stipulates that a maximum of four Lethal Ovitraps per property. There is no limit for Sticky Ovitraps or Clippy Ovitraps.
4. Place traps in the field for 2 to 4 weeks, can be checked weekly for water top up. Keep accurate records of locations and properties with ovitraps.

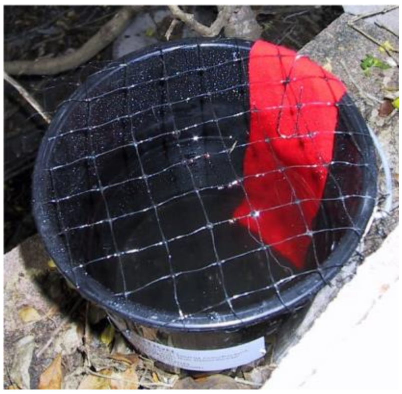

Fig. 7. Lethal Ovitrap

3. OVITRAP RETRIEVAL

1. Retrieve traps as required (1-2 weeks for Sticky and Clippy Traps, 3-4 weeks for Lethal Ovitraps);. Adult ID may be possible in the field using a hand lens. Eggs numbers assessment is best done in the lab.
2. Keep three random strips from each Dengue response, labelled (Batch No & Date) in a plastic bag and put in freezer in correct place.
3. Place the ovistrip in a container to prevent chemical contamination of the microscope when counting. Eggs can be counted / estimated with a hand lens or microscope.
4. Keep separate any mesh from Lethal Ovitraps and Sticky Ovitraps to avoid chemical contamination.

4. OVITRAP CLEAN UP

1. Dispose of the used ovistrips, glue panels and paper clips (into bin).
2. Wash buckets in hot water to remove any extra eggs and organic matter. Avoid harsh scrubbing of buckets to ensure mosquitoes lay eggs on ovistrips.

Preparing Large Quantities of Strips for Lethal Ovitraps

Additional Equipment

- Ply wood sheets (x 2), 1200 mm x 4 mm.
  'Jig A'. Cut 50 mm slots in ply wood sheet (Fig.1 LHS).
  'Jig B'. Cut 150 mm slots with the jig saw (Fig.1 RHS).

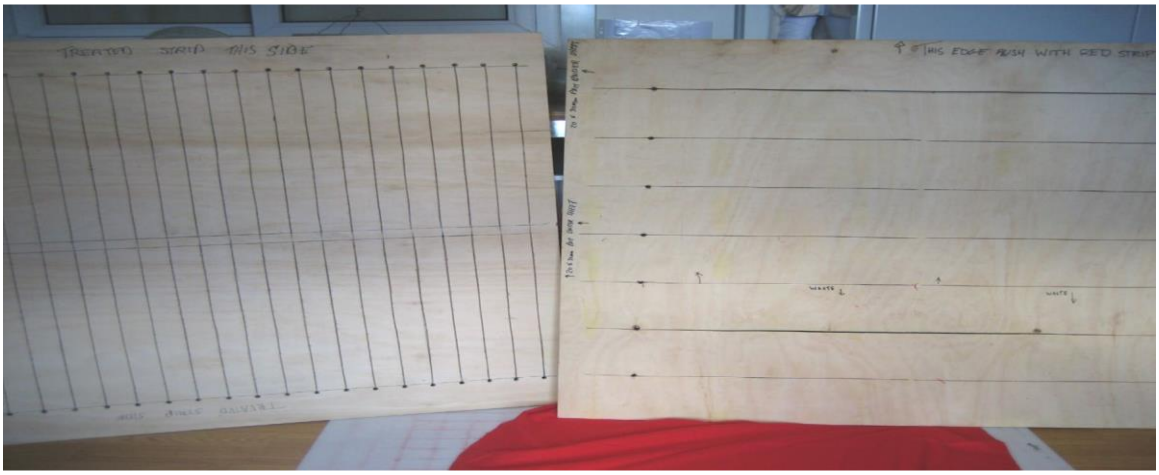

Fig.1. 2-Part Jig for ovistrip production. Jig A (LHS) , Jig B (RHS).

- Large Nylon Cutting Board (1250 mm) - Butcher supply shop
- Drying Rack (See Appendix 2).

Method

1. Ensure flannelette is to the left hand side of the cutting board with Five mm over hang at top.

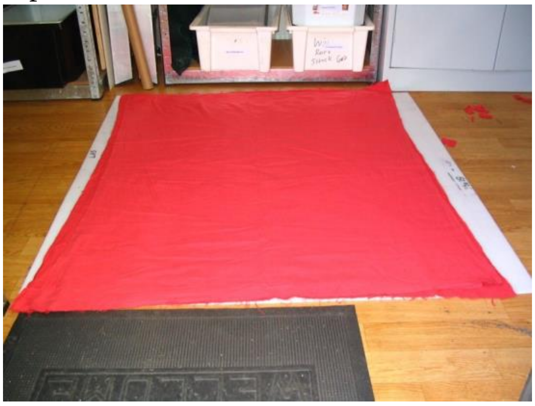

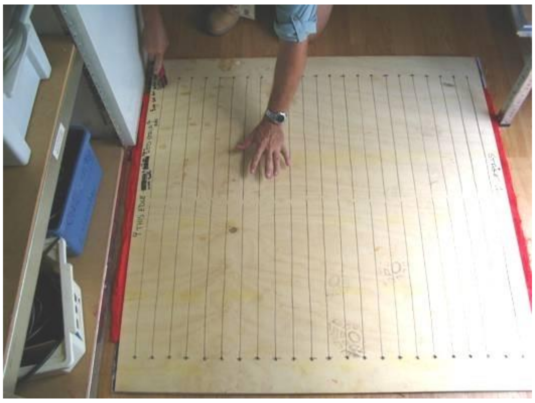

Fig. 2. Flannelette stretched on nylon cutting board.

Fig. 3. Jig A positioned over flannelette sheet.

This should be more towards Left Hand Side.

2. Place 'Jig A' 5 mm down from top of cutting board. Trim excess material from top and bottom of jig.
3. Run the blade along 50 mm slots - ensure cut goes through flannelette (see Fig. 3).
4. Taking care not to disturb the flannelette, remove 'Jig A'.
5. Cut flannelette free-hand in middle where 'Jig A' slots did not join.
6. Slide the cut flannelette Five mm past RHS edge of cutting board and Ten mm down from top of cutting board.
7. Place 'Jig B' on the cut flannelette, Five mm in from RHS and with 50 mm overhang at the top of the cutting board.
8. Run material blade along 150 mm slots, ensuring complete cut through the flannelette.
9. Remove 'Jig B', taking care not to disturb the flannelette.
10. Cut the flannelette free-hand on the end where 'Jig B' did not cut through.
11. Discard the waste; any flannelette that is not 150 mm x 50 mm or with badly frayed edges.
12. Pack cut strips into labelled brown paper bags 100 per bag.
13. Remember to take three random strips from each sheet of Material for testing. Put strips into plastic bag labelled with Treated Date Batch No. & person who treated material.

Construction of Drying Rack for Treated Ovistrips

1. Determine area required to dry treated material.
2. Construct lightweight wooden frame.
3. Attach shade cloth to wooden frame (eg. screws, heavy duty staples).
4. Attach small wheels or legs to elevate rack off the ground to keep material clean.

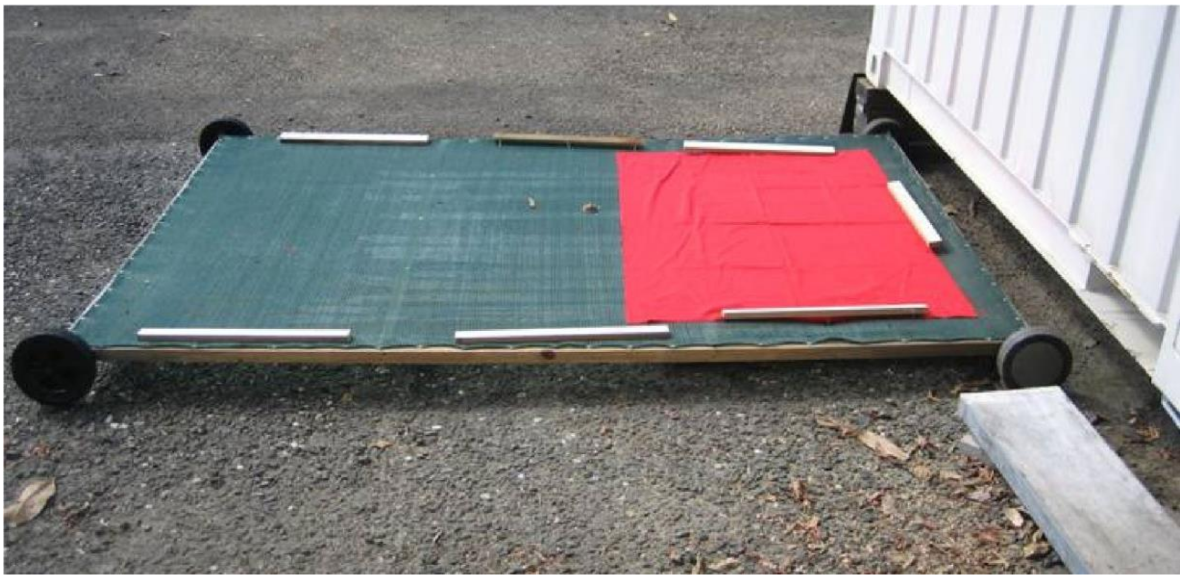

Fig. 1. Drying Rack with treated flanellette.

Appendix 3: BG-Sentinel Traps Procedures

<u>A. Setting and Maintenance</u>

13 May 2009

1.0 BG trap- Placement rules

- The trap needs to be set out of the wind, rain and direct sunlight.
- The trap should not be placed under any structures/objects where there is less than 1 m gap above the trap.
- Do not set the trap in any location where the top of the trap is over 1.5m off the ground, the closer to the ground the more effective the trap.
- Running the trap on mains power is preferred over battery operation. If the trap is to function correctly, then the battery must be 12V with a of minimum 24Ah output.

2.0 BG Trap components (see Fig. 1)

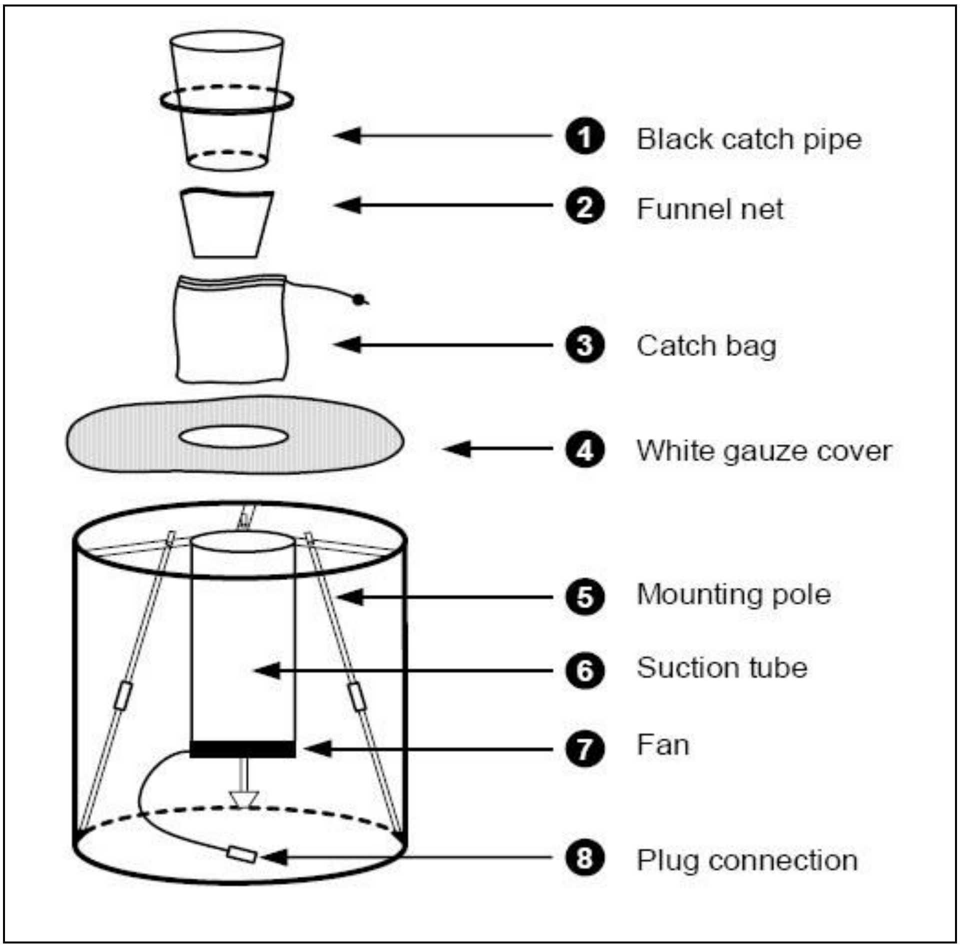

Fig. 1. Components of the BG Trap.

3.0 Trap construction.

3.1 Model A

1. Open the trap by undoing the two toggles on the side of the trap.
2. Insert the mounting poles into the loops on the floor of the trap and into the eyelets on the straps across the top of the trap (Fig. 2a).

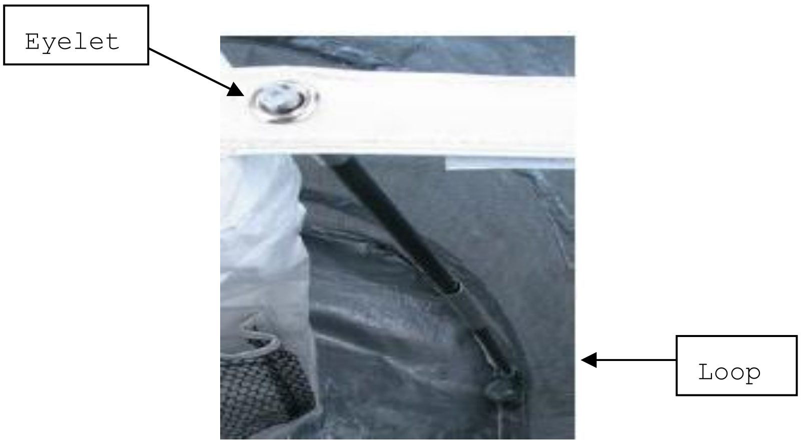

Fig. 2. Correct placement of the mounting poles in BG Trap Model A

3.2 Model B

1. Gently pull the trap into the upright position.
2. Slot the mounting poles into the gaps at the base of the trap, ensuring that the groove in the base of the pole is aligned over the bottom frame wire, very gently bend the pole until you are able to slot the top of the pole around the top frame wire. (See Fig. 2b)

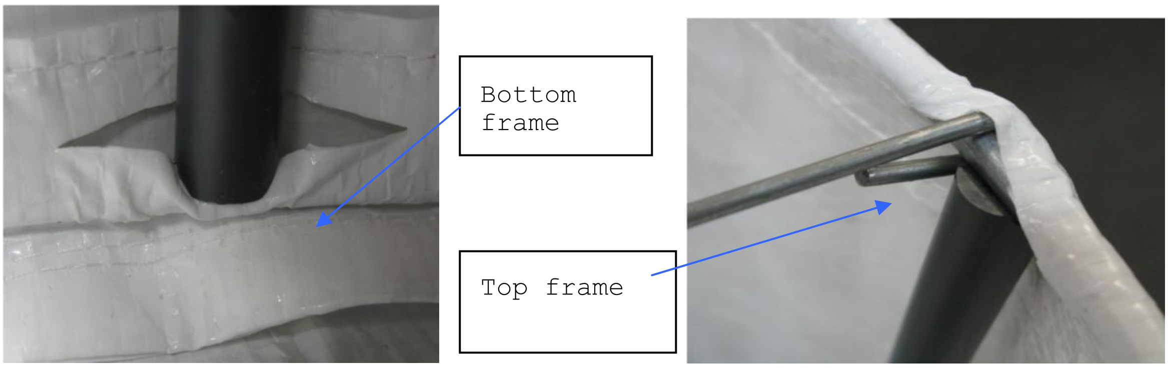

3. Gently push the pole so that it sits against the side of the trap. (see Fig. 3.)

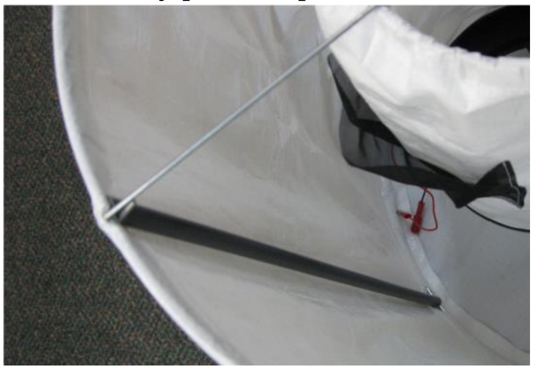

Fig. 3. BG Trap Model B with correct placement of the mounting poles.

3.3 Both Models

Once the mounting poles are in place the remaining trap construction is the same for both trap models

1. Ensure that the suction tube is fully expanded and the fan is not damaged.
2. Add the white gauze cover - ensure that the lead has not pulled the fan to one side of the trap. The cover must sit neatly over the trap with no gaps around the outside of the suction tube.(See Fig. 4)
3. The funnel net needs to sit high on the catch pipe (above the top of the groove on the catch pipe-Fig. 5) to allow the mosquitoes uninterrupted access to the catch bag. Once the funnel net is in place, the catch bag is placed over the funnel net. (Fig. 5)

4. Check that there are no holes or tears in the catch bag. The stitching of the catch bag MUST be on the outside of the bag to avoid mosquitoes becoming caught in the stitching and not being removed for identification. The catch bag requires regular inspection to avoid escapees (bag material is easily torn).

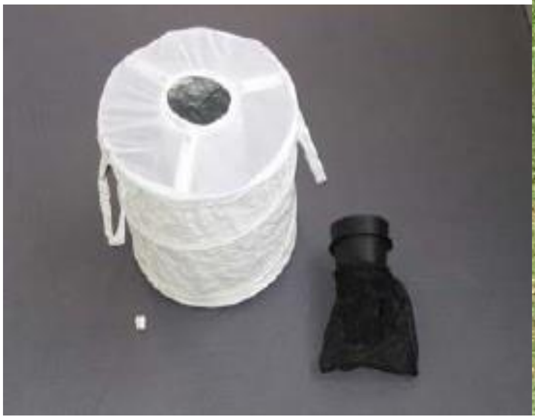

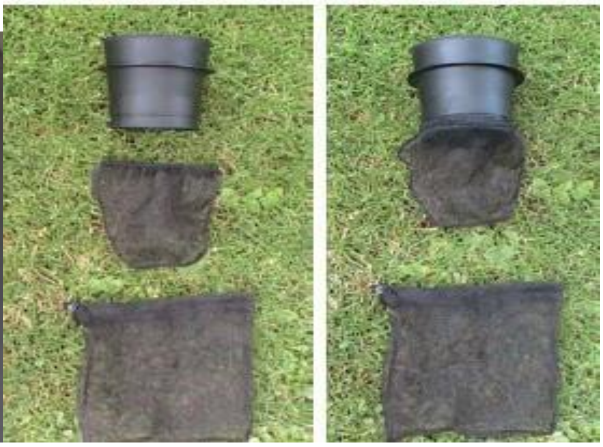

Fig. 4. BG Trap with white gauze cover in place **Fig. 5.** Correct placement of the funnel net.

5. Ensure there is a gap between the bottom of the funnel net and the catch bag. (Fig 6) Tighten the toggle on the catch bag until the bag is firm around the funnel net and catch pipe

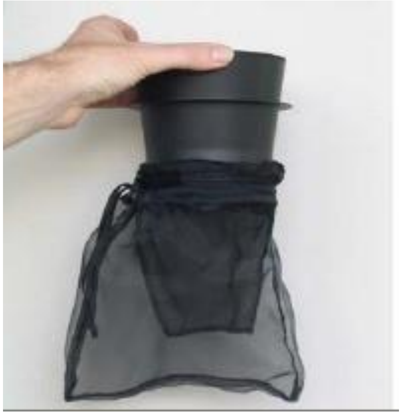

Fig. 6. Catch bag ready to be inserted into the BG trap.

6. Place the catch bag in the suction tube, the BG trap is now ready to be run. (Fig 6)

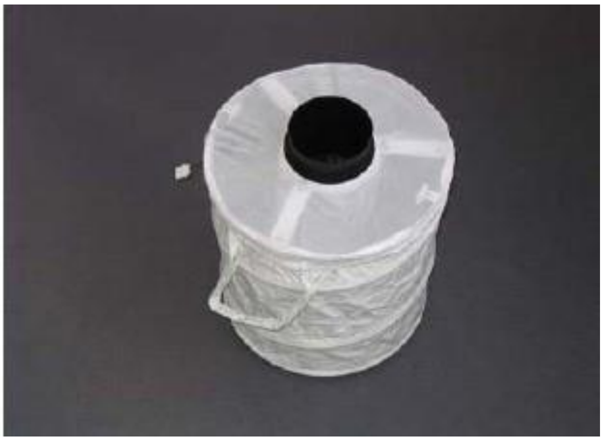

Fig. 7. The constructed BG Trap

4.0 Trap Operation.

1. Once the trap is running check the sound of the fan to ensure the fan is working and that the catch bag is not touching the fan. The sound of the fan will change if the catch bag, or part of the suction tube, is interfering with the airflow.

5.0 Collection of the catch bag.

The fan must be running when the catch bag is collected to prevent escapees.

1. Run your hand around the inside of the catch pipe and rattle the catch pipe to ensure that all the mosquitoes are inside the catch bag.
2. Carefully lift the catch pipe until the toggle of the catch bag is visible above the rim of the suction tube
3. Loosen the toggle until the catch pipe and funnel net can be removed from the catch bag.
4. Leaving the catch bag within the suction tube, tighten the toggle until firm and there is no gap in the top of the catch bag.
5. Attach label with trapping details to elastic of catch bag.

6.0 Transport of the collected catch bag

1. The label (collection details) for the catch bag should be attached to the elastic of the bag, preventing the label from falling off or damaging the collected mosquitoes.
2. Ideally, the collected mosquitoes will be transported within an esky to keep them alive and in good condition. A piece of dowel wedged in the esky, from which the catch bag can be hung, prevents the mosquitoes being squashed or badly rubbed during transport.
3. The label for the catch bags should be set out as below (Fig. 8)

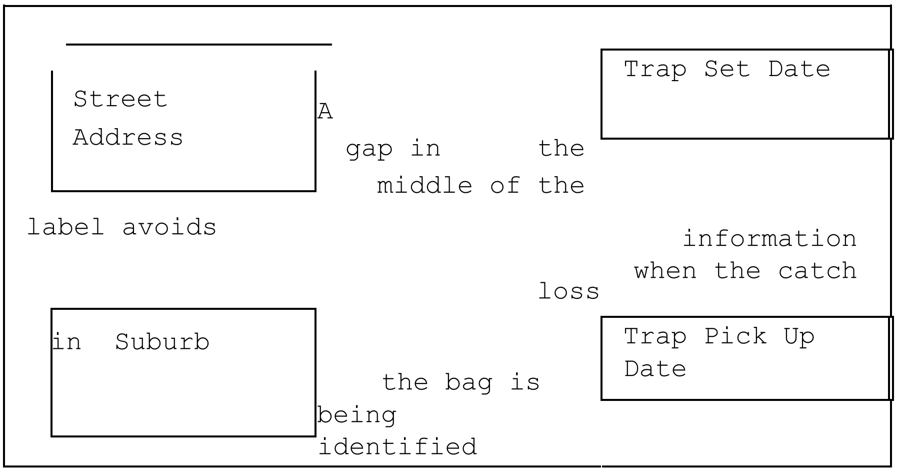

Fig. 8. Layout of label for catch bag

7.0 Trap maintenance.

7.1 White Gauze Cover

- The cover needs to be inspected regularly as they are prone to elastic degradation and can be torn.

- The cover requires washing (in warm soapy water) on a regular basis to keep it white and prevent dust interfering with the air flow.

7.2 Funnel Net

- The funnel net requires washing (in warm soapy water) on a regular basis to keep it clean and prevent dust interfering with the air flow. The elastic in the funnel net is also prone to degradation

7.3 Catch Bag

- Each time the catch bag is returned to the lab for the collection to be identified it MUST be carefully checked for holes, especially around the edges
- The catch bags also require regular washing to keep them clean and the airflow unhindered

8.0 Operational Notes

- The manufacturers (www.bg-sentinel.com) recommend that the catch bag be replaced every 3-4 days to maintain quality of the mosquito specimens. After this time the damage to the caught mosquitoes increases and identification becomes more difficult.
- If the trap is not working check the connecting plugs as sometimes the wire becomes loose and power doesn't reach the fan.
- CO_2 gas can be added to the trap to increase the catch of *Aedes aegypti*, as well as other mosquito species. The gas is added from the bottom of the trap, escaping upward.
- Ants can become a problem with long term surveillance trapping locations. Move the BG trap to a different location within the trapping property to deter the ants or treat the ground around the BG with surface spray (remove the BG trap from the area first and allow any excess chemical to dissipate before returning the trap). At no time should any part of the BG Trap be treated with chemical.
- The trap will work effectively without the funnel net, which is only required to assist in the prevention of insect escape if the power is interrupted.

Brisbane North Public Health Unit
Central Regional Services
Division of the Chief Health Officer
Queensland Health Lockbag 2,
Stafford QLD 4053.
(07) 3624 1111 fax
(07) 3624 1129
