# Supplementary material for: Extensive public health initiatives drive the elimination of Aedes aegypti (Diptera, Culicidae) from a town in regional Queensland: A case study from Gin Gin, Australia
Source: PLoS Negl Trop Dis. 2022 Apr 8;16(4):e0010243. doi: 10.1371/journal.pntd.0010243 (PMC9020727; doi:10.1371/journal.pntd.0010243)
Supplement: S2 Text — (PDF) [file pntd.0010243.s002.pdf]

Community Engagement Plan

As part of the Gin Gin Mark Release Recapture and Suppression plan, we hope to undertake an extensive community engagement process that informs and engages with all stakeholders in a way that is thorough and transparent. The strategy outlined below follows the framework and outcomes set by Queensland Health guidelines [1].

What is community engagement?

A democratic society revolves around the expectation that community members are able to have a say in processes that directly affect them. Community engagement is the process by which people are informed and involved in decisions affecting their lives. It enables high-quality governance and informed decision making by promoting shared responsibilities for decisions and outcomes. It provides a clear-cut approach to managing risk by providing a sturdy foundation for understanding decisions and building trust within the community on the decision-making process. Through sustained consultation and active participation, we seek to provide measurable health outcomes that benefit the entire community and region. WHO IRS guidelines state; "Community awareness and support are critical factors that influence the effectiveness of IRS programmes. To ensure that IRS is accepted by the local population, community education and communication campaigns must be implemented. Households should be well-informed about the programme and aware of its benefits" [2].

Principles of this community engagement plan:

1) Ensure the engagement is timely, accessible, planned and meaningful
2) Undertake engagement activities that overcome barriers to public participation and build the capacity of the community to participate in the proposed research.
3) Provide feedback to participants on the results of any research performed.
4) Review and evaluate with the community the effectiveness of our mosquito research and suppression work.

These values are informed by the International Association of Public Participation core values and reflect the CSIRO and QIMR Berghofer values of high scientific standards and integrity, better health and the prevention of disease through research.

Objectives of our community engagement:

1) To provide opportunities for the Gin Gin community to participate in the decision-making process and ensure outcomes are beneficial to the entire community. By involving all stakeholders in the planning process, we hope to create a sense of ownership of problems associated with faulty rainwater tanks and *Ae. aegypti* in the town.
2) To provide a strong foundation for understanding the research and suppression plan and promote a shared responsibility for decisions and trust in the decision-making process. This will include the formation of a Community Reference Group (CRG).
3) To develop a sustained collaboration in ways that involves and empowers the community to achieve measurable public health outcomes.

Community engagement activities:

Gin Gin is a small town and it is essential that the entire community is aware of research and suppression activities in their neighbourhood. There are a number of processes and strategies which can be leveraged to ensure stakeholders and fully informed, which include:

- Public forum and consultation
- Local media release
- Radio
- Letterbox drops and door knocking with information pack
- Website with satisfaction survey
- Notice board

Stakeholders as part of the engagement plan:

The consultation and engagement process will involve a number of discussions and negotiations with different levels of government, organisations and residents. Below is the list of stakeholders associated with this research and suppression plan. Each listing is the order in which stakeholders will be engaged.

1) Wide Bay Public Health Unit
2) Bundaberg Regional Council
3) Mary Burnett Vector Management Group
4) Queensland Department of Health, Communicable Diseases Unit
5) Gin Gin town residents and CRG
6) Individual residents around study sites

A number of local organisations will also need to be included in the community engagement, as they will be influenced by activities associated with the mosquito research plan. These include; the Gin Gin kindergarten, primary school and school principal, the Department of Education and Training and Gin Gin St Mary's Anglican church. Below is the estimated timeline for community engagement to take place (Table A).

Outcomes for councils and public health units:

- Data to inform future management of rainwater tanks and disease vectors in the region
- Greater community understanding of the roles and responsibilities of local council vector management and public health units
- Strengthened council capabilities in responding to potential disease outbreaks in the region through engagement and training by experienced and qualified vector management staff
- Opportunities to examine how health and vector management staff roles can engage with the broader community
- Opportunities for frontline council involvement in community engagement activities.
- Build awareness of the importance that rainwater tank infrastructure plays in the establishment of disease vectors
- Build awareness of the importance of PHU's role in leading community health outcomes

Outcomes for community of Gin Gin:

- Provide information to facilitate the identification of community priorities and expectations for the future of disease vectors in the region
- Build positive relationships and trust between council, Queensland Health staff and local communities
- Build community ownership of and connectedness with the councils objectives around rainwater tank infrastructure and the management of disease vectors
- Provide opportunities for all community members to participate in the development of long term goals for community health
- Foster common understandings and productive relationships between service delivery agencies such as the council and public health units.

- Help to solidify community expectations regarding the council's ability to service community needs.

Following up on engagement:

Engagement activities are undertaken to generate change within the community. The findings and outcomes of our research and suppression plan will be made available for the local community to access. Ongoing feedback will enable continued participation by stakeholders, clarify whether community issues have been understood and dealt with, assess the effectiveness of engagement and research outcomes, clarify research goals and objectives, improve relationships between all stakeholders and build trust and confidence in the engagement process overall.

Access to information and data will include:

- Details of total mosquitoes released and recaptured
- Total numbers of rainwater tanks surveyed and made compliant with regulations
- Answers to any concerns raised by community members
- Results of any feedback surveys
- Any changes that occurred to the research and suppression plan
- Details of any future engagement or follow up research

Feedback will be given in terms of written letters to all participants via email or post. Results and outcomes will be provided in a follow up presentation in a community forum similar to that performed before the research where the community will be acknowledged for their valuable contribution to the project. Public information releases will also be provided to local media, summarising the experiment's outcomes/benefits and gratitude to the Gin Gin community.

Community Engagement Timeline

Table A: Community Engagement Timeline

	September				October				November				December				January				February				April			
**Week**	1	2	3	4	1	2	3	4	1	2	3	4	1	2	3	4	1	2	3	4	1	2	3	4	1	2	3	4
**WBVMG**																												
**Council Meeting**																												
**QH Brisbane North**																												
**Town Meeting**																												
**Local Residents**																												
**Follow Up**																												

Community Reference Group terms of reference

The formation of the Gin Gin CRG is to ensure the researchers remain in touch with views of the community. To do this, the CRG provides a forum for community members to raise and discuss issues of relevance and concern that may arise through the activities undertaken by the research and suppression projects. The CRG provides a valuable tool for understanding community points of view when making decisions while also providing a mechanism to provide feedback on research and health outcomes. This terms of reference provides the framework and methodology for the community engagement undertaken by the researchers.

Role:

The CRG's role will be to aid communication between the community and the researchers. The CRG will:

- provide advocacy for the research project within the local community
- provide advice on how messages are expressed, including appropriate language and tone.
- provide assistance in directing messages through the appropriate channels relevant to the community (eg SMS, local papers, notice boards, community gatherings)
- provide a conduit for communication from the local community throughout the research project; including concerns or questions that may arise.
- facilitate feedback for results and outcomes of the research and suppression projects.

Ultimately it will serve as a way to facilitate community awareness of the disease risk posed by not maintaining rainwater tanks.

Membership:

The CRG should have a membership that reflects the community demographics and interest groups as closely as possible. Members of the CRG will be recruited from prominent members of the community of Gin Gin, as well as local residents involved with the research. This membership will be formed by 3 to 6 people from the area and will include an independent facilitator, Councillor Wayne Honor of the Bundaberg Regional Council.

Members will be encouraged to participate at least once a month, from December 2015 until April 2016. This may include a combination of phone discussions, online surveys or group workshops. Appointment to the group will finish when the adult trapping has ceased and results are made available to the community through a final presentation and a prominent community centre.

Members' Commitment:

Member support for the CRG is imperative to its ongoing success. The participants will commit to supporting the objectives of the CRG through regular attendance, recognition of the need to communicate and consult as well as appropriately responding to issues raised by the Group. Members will ensure any issues raised will be directed at the organisations involved and not at an individual. Responses to issues raised within the CRG will be provided in a timely manner once the facts are available. Issues will be placed on the agenda prior to any meetings to ensure a prepared response can be delivered. Members will be respectful and listen to the opinions of others and recognize that participation in the CRG forum is crucial to the success of the project.

Reference

1. Queensland Government. Consumer and Community Engagement Framework. In: Health Q, editor. 2012. p. 32.
2. World Health Organisation. Indoor residual spraying: An operational manual for IRS for malaria transmission, control and elimination. Second edition ed. 20 Avenue Appia, 1211 Geneva 27, Switzerland: World Health Organisation; 2015. p. 134.
