# Supplementary material for: Extensive public health initiatives drive the elimination of Aedes aegypti (Diptera, Culicidae) from a town in regional Queensland: A case study from Gin Gin, Australia
Source: PLoS Negl Trop Dis. 2022 Apr 8;16(4):e0010243. doi: 10.1371/journal.pntd.0010243 (PMC9020727; doi:10.1371/journal.pntd.0010243)
Supplement: S3 Text — Table A: Hazard Analysis and Risk Assessment. (PDF) [file pntd.0010243.s003.pdf]

Risk Management Strategy

Hazard Identification

Potential hazards associated with the release of *Aedes aegypti* into Gin Gin are listed in Table A and outlined below. As the species is already established in the town, it exists at a low density and there is a possibility that collections of this small population may result in insufficient data.

Table A: Hazard Analysis and Risk Assessment

Hazard	Type	Description	Risk	Potential Impact
Vector in the environment	Community	Create awareness of disease vector in the environment	Low	Vector is already present in community
Increased vector population	Biological	Create a larger number of mosquitoes in the environment	Moderate	Higher perceived risk of disease
Imported disease case	Health	Dengue notified in the town during experiment	Low	Highest threat to community and study
Increased mosquito range	Biological	Risk of spread of the vector to other part of the town or towns	Moderate	Spread to towns further south or north
Increased host biting	Community	Awareness or increase in mosquito biting	Low	Nuisance mosquitoes
Negative media attention	Community	Perceived increase risk of disease	Low	Increased interest by wider community
Sting or bite from dangerous animal	Study Team	Aggressive dog attack, wasp sting, spider bite.	Moderate	Hospitalization
Low recapture rate of *Ae. aegypti*	Research	Low population of the *Ae. aegypti* in Gin Gin may lead to low or no recaptures in traps	Moderate	No data for research

Increased awareness of a vector in the environment

Releasing mosquitoes into the environment draws the attention of the local community to a mosquito population that is already present. From previous surveys, the mosquito is established in the community and homeowners are aware of its presence.

Increased vector population

The nature of a mark, release, and recapture study involves the marking and release of organisms into the environment. An elevated number of mosquitoes will enter the environment and may temporarily increase exposure of residents to mosquito bites. However, the timing of the release will occur when all mosquito species are in peak numbers in the environment.

Imported disease case

The release of disease vectors into Gin Gin and the associated potential increase in exposure to mosquito bites has a very small risk of disease spread. Dengue fever has not been documented in the town during the past 25 years, suggesting that opportunities for local transmission are rare and unlikely to occur during the study period. However we cannot rule out the possibility of an imported dengue case arriving during the study.

Increased population range

As the mosquito is already established throughout the town, there is a very small risk that the introduction of more mosquitoes will increase the local population range. The dispersal range of *Ae. aegypti* is considered very small, and it is unlikely that we will observe movement towards high

traffic areas during the two weeks of the release. There is a small risk that mosquitoes may enter cars and travel to towns north or south of Gin Gin.

Negative media attention

This is a small risk that local media could use the study to create negative media towards the council, public health units or research organisations.

Personal injury due to aggressive behaviour, sting or bite from a dangerous animal

There is a moderate risk of dogs acting aggressively towards survey teams entering properties. There is a small risk of exposure to biting arthropods in yard surveys and trap placement. Risk of disease from a mosquito bite is addressed in the previous disease hazard.

Low recapture rate of *Ae. aegypti*

There is a moderate chance that there are small *Ae. aegypti* egg banks within in the rainwater tanks selected as the release sites.

Mitigation Strategy

Increased perception of a vector in the environment

Our community engagement aims to ensure that all residents that may be affected during the release will be informed and given the opportunity to voice their concerns. It has been recommended that the community provided the opportunity to form a reference committee to assist with managing perceived risk. This will involve the formation of this group at a community meeting, with 3-6 members to be selected from residents, representatives from the Bundaberg Regional Council and the Wide Bay Public Health Unit. This group will be in a position to answer any community questions and act as a conduit to the project team for any concerns raised.

To lower concerns about an increase in mosquito numbers we will provide all residents (within the study blocks) with repellent to be used to mitigate any short term in nuisance biting. After the study has been completed and to align with the subsequent suppression program, we will also offer insecticide spray to all homeowners at the release sites.

Increased vector population

The release will occur when all mosquito species are in peak abundance in the urban environment so we anticipate that the proportional increase in biting pressure will be minimal. The total number of mosquitoes released will be controlled through the use of emergence traps on rainwater tanks. These traps capture emerging mosquitoes and allow them to be marked for release. It is unknown how many mosquito eggs are inside the tanks. However, we estimate that no more than 1000 mosquitoes of each species will be released from tanks in what would be considered a normal rain event, with the remainder being sealed inside the tanks and treated with chemicals.

To minimize the risk of an increased vector population we intend to apply a suppression program using a number of control mechanisms. We believe that any increase in vector population numbers will be temporary due to the suppression program and the release being at a point in the year where all mosquito species are at peak abundance. The use of chemicals in the suppression plan will target adults that have survived the release. All non-compliant tanks within 200m will be sealed and made to be compliant with Queensland government regulations. This will remove a major source of recruitment to the *Ae. aegypti* population around the release area. If suppression is effective, there will be a lower abundance of *Ae. aegypti* in the study area as measured by our pre-and-post trapping.

An El Niño is forecast to occur the summer of this experiment. This climatic event brings with it dryer than normal conditions which would be favourable for suppression of *Ae. aegypti*, who are reliant upon water around human dwellings. The lower rainfall and the sealing of non-compliant tanks will combine synergistically with the suppression program to decrease the likelihood that mosquito populations will increase in after the release.

We acknowledge that there is a risk that the abundance of the *Ae. aegypti* will increase if suppression is ineffective. In this case there will need to be consultation between local residents, public health units and Bundaberg Regional Council to determine if another round of suppression is required. A subsequent round of suppression may require greater coverage of the area including truck mounted ULV treating and further IRS application and larval site removal (including rainwater tanks) in areas up to 300m from the release site.

Disease risk

This risk of dengue transmission will be managed through close engagement with the Public Health Unit which receives notification of new dengue fever cases. We plan to engage with local General Practitioners to make them aware of our work and to be conscious of any potential dengue-like symptoms that may present to patients. If Queensland Health are notified of a viraemic case of dengue fever in Gin Gin, we will implement the control and suppression operation immediately (ceasing the experiment) and expand the suppression to around the household and surrounding blocks to where the disease case was recorded, following standard Queensland Health procedures.

Increased population range

The site of release is more than 200m away from the highway that leads through the town and previous studies have indicated that *Ae. aegypti* is unlikely to disperse more than 175m from their original emergence source over a two week period [1]. Adult trap placement has been designed to limit the movement of adult mosquitoes towards the highway by interception. However, it must be noted that the species has been detected in premises along the highway during previous surveys.

Negative media attention

We plan to have close engagement with the community, local council and public health units as well as ethics approval from the organisations representing the research and we will receive endorsement before any research is undertaken. We plan to inform the community well in advance of research, and the population has been very helpful in previous survey work. Our community consultation will be an open strategy that seeks to engage and educate all stakeholders, in an effort to present all research goals and outcomes in a transparent way. In the case that negative media does arise, all organisations involved in the planning process and with the help of communication teams, will seek to provide clear and concise responses to any concerns focusing on positive health outcomes to the community. It will be made clear that the ultimate goal of the research is to increase awareness of the risks posed by non-compliant rainwater tanks in Gin Gin on potential disease transmission and vector spread.

Personal injury due to aggressive behaviour, sting or bite from a dangerous animal

Our goal will be to prevent unnecessary exposure to dangerous animals. To do this we will reduce exposure to insect bites by taking care when moving around properties and the use of repellent; avoid potential dangerous or aggressive householders or dogs; and develop safety measures in collaboration with OH&S staff. A first aid kit will be available with itch removing cream (such as Stingoes), an antiseptic cream for more serious bites and cuts and other general purpose safety items. CSIRO staff have all been trained in first aid. If it is essential that we access a premises, we will

leave documentation requesting urgent access or require dogs to be restrained. Any serious injury will be taken to the hospital located on the north side of Gin Gin.

Low recapture rate of *Ae. aegypti*

To mitigate the risk of low recaptures, the design of the experiment maximises the recapture rate of *Ae. aegypti*. To do this we are using the most effective trap available for collecting the species – the BG Sentinel trap. These traps have been proven to be the most sensitive to container breeding mosquitoes. To enhance potential mosquito recaptures, we will mark another species of mosquito, *Ae. notosctriptus,* which has also been observed as larvae inside the proposed release tanks.

References

1. Russell RC, Webb C, Williams C, Ritchie S. Mark–release–recapture study to measure dispersal of the mosquito *Aedes aegypti* in Cairns, Queensland, Australia. Med Vet Entomol. 2005;19(4):451-7.
