# Supplementary material for: Extensive public health initiatives drive the elimination of Aedes aegypti (Diptera, Culicidae) from a town in regional Queensland: A case study from Gin Gin, Australia
Source: PLoS Negl Trop Dis. 2022 Apr 8;16(4):e0010243. doi: 10.1371/journal.pntd.0010243 (PMC9020727; doi:10.1371/journal.pntd.0010243)
Supplement: S4 Text — Table A: Timeline of suppression events including time budgeted for activity Table B: Staff availability and required for suppression program. (PDF) [file pntd.0010243.s004.pdf]

Mosquito Suppression Plan

Background and Guidelines

The primary role of this suppression plan is to simulate a response to an imported case of dengue into the Wide Bay area. This response is dictated by the Queensland Dengue Management Plan (DMP)[1].

The Queensland Pest Management Act 2001 [2] requires all mosquito control activities involving the use of pesticides to be conducted by a licensed pest management technician. The Queensland DMP states that upon receipt of a dengue notification and subsequent risk assessment undertaken, the appropriate mosquito control must be applied to limit the extent of further disease spread. These activities include larval control, adult control and community engagement.

Larval control consist of intensive inspections of all yards within 200m of the case house (and other places visited during viraemic periods) and controlling mosquito larvae in containers. Containers can be emptied of water and rendered mosquito proof. Chemical control is done via the application of Prolink Pellets containing the growth regulator (S)-methoprene and offers the residual activity of one month duration due to slow release formulation. Prolink XR-Briquets contain (S)-methoprene and are recommended by the Dengue Management Plan as a temporary measure for treating rainwater tanks. Residents are made aware that rainwater tanks must be screened to comply with the Public Health Regulation 2005 [3].

Adult control is based on Queensland research showing that focal area control (radius of 200m from the viraemic case) is successful at limiting disease spread. This response is more effective if control is initiated within four days of dengue infection or the disease carrying person enters the location. Interior residual spraying (IRS) is the application of a long-lasting, residual insecticide to mosquito resting surfaces within houses or structures where dengue vectors might come into contact with the insecticide. IRS is most effective against indoor feeding (endophagic) and indoor resting (endophilic) vectors and therefore a very effective way to kill adult *Ae. aegypti* around human habitation [4]. The residual insecticides used include bifenthrin, deltamethrin or lambda-cyhalothrin. Premises occupants must grant permission for treatment and are provided with information about the chemicals and safety precautions. Truck based 'fogging' is highly visible and popular internationally and although it is not effective at controlling *Ae. aegypti*. It is effective at reducing mosquito species outside human habitation and will lower the abundance of other mosquito species already in the environment or associated with the research (*Ae. notoscriptus*).

Gin Gin Mosquito Population Suppression

In responding to an imported case of dengue fever, the goal of any suppression plan is to reduce or prevent virus transmission through the control of mosquito vectors or to interrupt human-vector contact [5]. We propose to apply the DMP methodology to suppress any mosquitoes released as part of the simulated rain event and mark recapture study. This process will be divided into four stages; pre-surveillance, tank survey and larval control, chemical suppression and post surveillance. The primary outcomes of the suppression phase of this project will aim to have a substantial and long lasting impact on the local *Ae. aegypti* population in Gin Gin.

Pre-and-post-release surveillance

Pre-and-post-release surveillance will use BG traps and GATs in five properties considered high risk along the main commercial areas of the town. Adult trapping will occur for 3 weeks prior to the release of mosquitoes (Table 2). Traps will be serviced and species identified weekly by local council

vector control teams and other available staff. Data will be recorded and stored through the Mobile Mozzie trapping and surveillance application. Following chemical suppression work, traps will be serviced weekly by local council staff and Brendan Trewin and run until early April and the beginning of colder and dryer winter conditions. Pre-and-post surveillance activities are designed to monitor the effectiveness of the larval and chemical suppression components.

Tank Survey

The location and condition of rainwater tanks within 200m of release sites will be identified and examined for compliance with regulations. Two teams consisting of two staff members will go from house to house and assess the condition of an estimated 42+ rainwater tanks within 200m of the release sites. If tanks are non-compliant, recordings will be made of faults and a mosquito survey will be performed. The five-sweep methodology of Knox et al. [6], will be implemented and samples will be stored in 80% ethanol for identification. All tanks that are non-compliant with regulations will be sealed to the entry/exit of mosquitoes, treated with (S)-methoprene or removed. Larval source reduction will occur among houses surveyed within 200m of release sites through emptying of containers or control using (S)-methoprene.

Chemical Suppression

Chemical suppression will be informed by Queensland DMP for control of *Ae. aegypti* with adulticides applied primarily as surface treatments. The objectives of IRS as defined by the WHO are:

1) to reduce the vector's lifespan to less than the extrinsic incubation period of the virus
2) to reduce vector density by immediately killing
3) to reduce human-vector contact

Outcomes of this type of intervention rapidly reduces the vector's transmission capacity by providing the maximum effect on vector populations. The WHO IRS guidelines state that a "successful IRS operation requires a high level of political commitment, dedicated human, logistic, transport and financial resources, and adequate organizational and planning capacity" [4].

In order to plan an IRS campaign it is essential to know the number of houses, the average number of rooms per household and the average size of one room. This information is used to estimate the total 'sprayable surface' area before insecticide quantification and procurement. To do this we will sample 5-10% of houses during the tank survey as a representative sample. Sprayable surface is defined as the inside surfaces of all structures or houses that should be sprayed and includes eaves, ceilings, under-floor areas in raised housing, inside walls of latrines under furniture and dark corners.

The goal of the chemical suppression work is to reduce adult mosquito numbers following the cessation of the recapture experiment. This would occur no longer than two weeks after the last date of release. We will seek approval from homeowners within 200m of the release sites to conduct IRS and these properties have been identified by GIS software. We will seek approval from homeowners at-and-next to the release site to apply barrier treatments to external foliage and resting sites. There will be the potential to use this exercise as a training response to an actual case of dengue in the region. This would therefore involve the use of IRS, which is the standard procedure used for a dengue response by Queensland Health in Cairns. All application of chemicals will be done by qualified staff with pest control licenses. Successful IRS spraying campaigns depend on the application of a suitable and uniform dosage of insecticide on all resting places. This requires the appropriate training of spray operators so we have in-principle support from the Cairns Public Health Unit to train operators in the application of IRS. Travel costs and accommodation associated with providing DART staff will be funded by QIMRB/CSIRO research funding. The Wide Bay Public Health

Unit has offered in-principle support for suppression work and we are seeking council vector control officers interested in IRS training.

All consenting properties within 200m of the release site will be treated with synthetic pyrethroids chosen specifically for their safe use for humans. These chemicals have a minimum required residual period of two to six months. It is estimated that to apply IRS to 42 properties within 200m and barrier treatments to foliage around release sites, it will take a team of four sprayers three days (Greg Devine, personal communication). Staffing requirements and availability is displayed in Table 3. Application of pyrethroids will be via hand-operated compression air sprayers by two teams of two spray members using protective clothing including googles, masks, rubber gloves and long sleaved overalls. All use of chemicals will be recorded to house level and collected for reporting by qualified pest inspectors. All residents of properties that receive chemical treatment will be provided with a Pest Control Advice as per section 13 of the Pest Management Regulation 2003 [7]. All non-compliant tanks (damaged/missing screens) identified within 200m will be made compliant, treated with (S)-methoprene or removed. Finally, it has been recommended that truck mounted ULV be applied along streets to a 200m radius of the release site.

Table A: Timeline of suppression events including time budgeted for activity

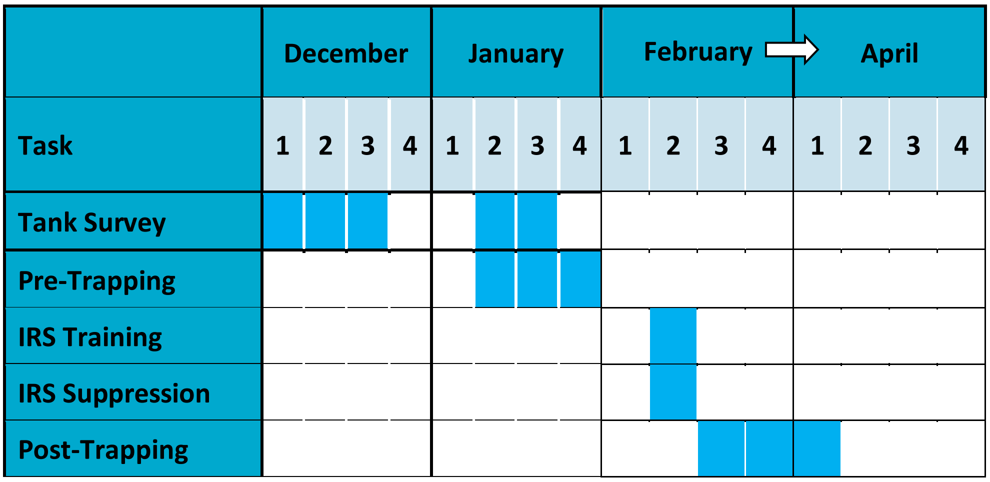

Table B: Staff availability and required for suppression program

Task	Available Staff	Time	Required Staff	Time
Tank Survey	1 x CSIRO<br>1 x Student	2 weeks, October and December	2	1-4 weeks, October until January
Pre-Trapping	1 x UQ/QIMR<br>1 x Student	3 days	Staff from tank survey	
IRS, Barrier Sprays and Training	1 x Cairns PHU	3 days	3-4	2 days
Post-Trapping	1 x Student	1 day a fortnight, 6 weeks	1	1 day a fortnight, 6 weeks

Reference

1. Queensland Government. (2010). *Queensland Dengue Management Plan*. 15 Butterfield St, Herston Qld 4006, Fortitude Valley BC 4006: Queensland Health.
2. Queensland Government. (2001). *Queensland Pest Management Act 2001*. Retrieved from https://www.legislation.qld.gov.au/LEGISLTN/CURRENT/P/PestManA01.pdf.
3. Queensland Government. (2005). *Public Health Regulation 2005*. Brisbane.
4. World Health Organisation. (2015). *Indoor residual spraying: An operational manual for IRS for malaria transmission, control and elimination.* 20 Avenue Appia, 1211 Geneva 27, Switzerland: World Health Organisation Retrieved from http://www.who.int/malaria/publications/atoz/9789241508940/en/.
5. World Health Organisation. (2009). *Dengue Guidelines For Diagnosis, Treatement, Prevention And Control*. 20 Avenue Appia, 1211 Geneva 27, Switzerland: World Health Organisation Retrieved from http://www.who.int/rpc/guidelines/9789241547871/en/.
6. Knox, T. B., Yen, N. T., Nam, V. S., Gatton, M. L., Kay, B. H., & Ryan, P. A. (2007). Critical evaluation of quantitative sampling methods for Aedes aegypti (Diptera: Culicidae) immatures in water storage containers in Vietnam. *Journal of Medical Entomology, 44*(2), 192-204.
7. QLD Government. (2003). Pest Management Regulation 2003.
