# Supplementary material for: Extensive public health initiatives drive the elimination of Aedes aegypti (Diptera, Culicidae) from a town in regional Queensland: A case study from Gin Gin, Australia
Source: PLoS Negl Trop Dis. 2022 Apr 8;16(4):e0010243. doi: 10.1371/journal.pntd.0010243 (PMC9020727; doi:10.1371/journal.pntd.0010243)
Supplement: S1 Table — Table A. Samples of Aedes aegypti larvae for genetic analysis taken from Gin Gin, Queensland during summer 2012/13. Table B. Number of genetic clusters (K) assigned by STRUCTURE in a sample of Aedes aegypti from Gin Gin, Queensland, Australia. Figure A. Genetic clusters assigned by STRUCTURE for samples of Aedes aegypti from Queensland, Australia. (PDF) [file pntd.0010243.s005.pdf]

Table A. Samples of *Aedes aegypti* larvae for genetic analysis taken from Gin Gin, Queensland during summer 2012/13.

Sample No.	Container	Number
10622	Bucket	1
10659	Bucket	2
16993	Cup	3
16980	Ice Cream Tub	3
10542	Ice Cream Tub	3
16985	Pool	1
10588	20 L Drum	3
10574	Pot & Base	1
18320	Pot & Base	2
10559	Pot & Base	1
10676	Esky	3
10649	Dog Bowl	1
10661	Kettle	3
10664	Galvanised Tank	1
18341	Self Watering Pot	1
16911	Tyre	1
10553	Tyre	1
16923	Ice Cream tub	1
16957	Dog Bowl	3
14200	Pot	3
16950	Drum Lid	1

Table B. Number of genetic clusters (K) assigned by STRUCTURE in a sample of *Aedes aegypti* from Gin Gin, Queensland, Australia.

# K	Reps	Mean LnP(K)	Stdev LnP(K)	Ln'(K)	|Ln''(K)|	Delta K
1	5	-3954.1200	0.0447	NA	NA	NA
2	5	-3856.9000	20.4043	97.220000	42.520000	2.083876
3	5	-3802.2000	29.8582	54.700000	1.220000	0.040860
4	5	-3748.7200	15.4135	53.480000	35.500000	2.303171
5	5	-3730.7400	16.3497	17.980000	17.760000	1.086258
6	5	-3695.0000	18.8654	35.740000	71.800000	3.805900
7	5	-3731.0600	22.9265	-36.060000	37.760000	1.647005
8	5	-3729.3600	30.4429	1.700000	NA	NA

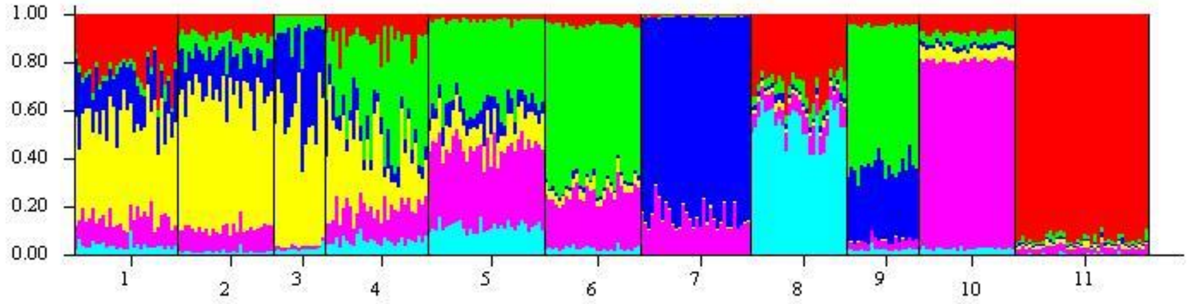

Figure A. Genetic clusters assigned by STRUCTURE for samples of *Aedes aegypti* from Queensland, Australia (1=Yorkeys Knob, 2=Gordonvale, 3=Ingham, 4=Rockhampton, 5=Mt Morgan, 6=Duaringa, 7=Bluff, 8=Emerald, 9=Capella, 10=Longreach, 11=Gin Gin)
