# Supplementary material for: Extensive public health initiatives drive the elimination of Aedes aegypti (Diptera, Culicidae) from a town in regional Queensland: A case study from Gin Gin, Australia
Source: PLoS Negl Trop Dis. 2022 Apr 8;16(4):e0010243. doi: 10.1371/journal.pntd.0010243 (PMC9020727; doi:10.1371/journal.pntd.0010243)
Supplement: Attachment — Submitted filename: PNTD-D-21-00837.pdf [file pntd.0010243.s006.pdf]

PLOS Neglected Tropical Diseases

Extensive public health initiatives drive the elimination of Aedes aegypti from a town in regional Queensland: a case study from Gin Gin, Australia.

--Manuscript Draft--

**Manuscript Number:**	PNTD-D-21-00837
**Full Title:**	Extensive public health initiatives drive the elimination of Aedes aegypti from a town in regional Queensland: a case study from Gin Gin, Australia.
**Short Title:**	Elimination of Aedes aegypti from Gin Gin, a regional town in Queensland, Australia
**Article Type:**	Research Article
**Keywords:**	Elimination; Aedes aegypti; Public Health; Community Engagement; Rainwater Tank; Population Genetics
**Abstract:**	Aedes aegypti is the primary vector of exotic arboviruses (dengue, chikungunya and Zika) in Australia. Once established across much of Australia, this mosquito species remains prevalent in central and northern Queensland. In 2011, Ae. aegypti was re-discovered in the town of Gin Gin, Queensland, by health authorities during routine larval surveillance. This town is situated on a major highway that provides a distribution pathway into the highly vulnerable and populous region of the state where the species was once common. Following the detection, larval habitat and adult control activities were conducted as a public health intervention to suppress the Ae. aegypti population and reduce the risk of exotic disease transmission. Importantly, genetic analysis revealed a homogenous cluster and small effective population vulnerable to elimination. By 2015, the population had expanded throughout the centre of the town. In response, a collaboration between research agencies and local stakeholders activated a council initiative in 2016 that included extensive community engagement, enhanced entomologic surveillance and vector control activities including the targeting of key containers such as unsealed rainwater tanks. Here we describe a model of the public health intervention which successfully reduced the Ae. aegypti population below detection thresholds, using source reduction, insecticides and novel, intensive genetic surveillance methods. This outcome has important implications for future elimination work in small towns in vulnerable regions and reinforces the longstanding benefits of a partnership model for public health-based interventions for invasive urban mosquito species.
**Additional Information:**	
**Question**	**Response**
**Financial Disclosure**<br><br>Enter a financial disclosure statement that describes the sources of funding for the work included in this submission. Review the submission guidelines for detailed requirements. View published research articles from *PLOS NTDs* for specific examples.<br><br>This statement is required for submission and **will appear in the published article** if the submission is accepted. Please make sure it is accurate.	NMEH was supported by National Health and Medical Research Council program grants 1037003 & 1132412. Funders did not play any role in study design, data collection and analysis, decision to publish or preparation of the manuscript.<br>https://www.nhmrc.gov.au/

Powered by Editorial Manager® and ProduXion Manager® from Aries Systems Corporation

**Unfunded studies**<br><br>Enter: *The author(s) received no specific funding for this work.*<br><br>**Funded studies**<br><br>Enter a statement with the following details:<br>• Initials of the authors who received each award<br>• Grant numbers awarded to each author<br>• The full name of each funder<br>• URL of each funder website<br>• Did the sponsors or funders play any role in the study design, data collection and analysis, decision to publish, or preparation of the manuscript?<br>• **NO** - Include this sentence at the end of your statement: *The funders had no role in study design, data collection and analysis, decision to publish, or preparation of the manuscript.*<br>• **YES** - Specify the role(s) played.<br><br>* typeset	
**Competing Interests**<br><br>Use the instructions below to enter a competing interest statement for this submission. On behalf of all authors, disclose any competing interests that could be perceived to bias this work—acknowledging all financial support and any other relevant financial or non-financial competing interests.<br><br>This statement **will appear in the published article** if the submission is accepted. Please make sure it is accurate. View published research articles from *PLOS NTDs* for specific examples.	The authors have declared that no competing interests exist.

**NO authors have competing interests**<br><br>Enter: *The authors have declared that no competing interests exist.*<br><br>**Authors with competing interests**<br><br>Enter competing interest details beginning with this statement:<br><br>*I have read the journal's policy and the authors of this manuscript have the following competing interests: [insert competing interests here]*<br><br>* typeset<br><br>This statement is required for submission and **will appear in the published article** if the submission is accepted. Please make sure it is accurate and that any funding sources listed in your Funding Information later in the submission form are also declared in your Financial Disclosure statement.	
**Data Availability**<br><br>Authors are required to make all data underlying the findings described fully available, without restriction, and from the time of publication. PLOS allows rare exceptions to address legal and ethical concerns. See the PLOS Data Policy and FAQ for detailed information.	Yes - all data are fully available without restriction

Powered by Editorial Manager® and ProduXion Manager® from Aries Systems Corporation

A Data Availability Statement describing where the data can be found is required at submission. Your answers to this question constitute the Data Availability Statement and **will be published in the article**, if accepted.<br><br>**Important:** Stating 'data available on request from the author' is not sufficient. If your data are only available upon request, select 'No' for the first question and explain your exceptional situation in the text box.<br><br>Do the authors confirm that all data underlying the findings described in their manuscript are fully available without restriction?	
**Describe where the data may be found in full sentences. If you are copying our sample text, replace any instances of XXX with the appropriate details.**<br><br>• If the data are **held or will be held in a public repository**, include URLs, accession numbers or DOIs. If this information will only be available after acceptance, indicate this by ticking the box below. For example: *All XXX files are available from the XXX database (accession number(s) XXX, XXX.).*<br>• If the data are all contained **within the manuscript and/or Supporting Information files**, enter the following: *All relevant data are within the manuscript and its Supporting Information files.*<br>• If neither of these applies but you are able to provide **details of access elsewhere**, with or without limitations, please do so. For example:<br><br>*Data cannot be shared publicly because*	All relevant data are within the manuscript and its Supporting Information files.

of [XXX]. Data are available from the XXX Institutional Data Access / Ethics Committee (contact via XXX) for researchers who meet the criteria for access to confidential data.<br><br>*The data underlying the results presented in the study are available from (include the name of the third party and contact information or URL).*<br>• This text is appropriate if the data are owned by a third party and authors do not have permission to share the data.<br><br>* typeset	
Additional data availability information:	

Powered by Editorial Manager® and ProduXion Manager® from Aries Systems Corporation

manuscript

Click here to access/download;Manuscript;GinGinElimination of Aedes aegypti_Final01_NoEndnote.docx

Extensive public health initiatives drive the elimination of *Aedes aegypti* from a town in regional Queensland: a case study from Gin Gin, Australia.

Brendan J. Trewin[1], Brian L. Montgomery[2], Tim P. Hurst[2], Jason S. Gilmore[2], Nancy M. Endersby[3], and Greg Crisp[2].

1. CSIRO, Health & Biosecurity, Queensland, Australia.
2. Queensland Health, Queensland, Australia.
3. University of Melbourne, Bio21 Institute, Victoria, Australia.

Key words: Elimination, Aedes aegypti, Public Health, Community Engagement, Rainwater Tank, Population Genetics

Abstract

Aedes aegypti is the primary vector of exotic arboviruses (dengue, chikungunya and Zika) in Australia. Once established across much of Australia, this mosquito species remains prevalent in central and northern Queensland. In 2011, *Ae. aegypti* was re-discovered in the town of Gin Gin, Queensland, by health authorities during routine larval surveillance. This town is situated on a major highway that provides a distribution pathway into the highly vulnerable and populous region of the state where the species was once common. Following the detection, larval habitat and adult control activities were conducted as a public health intervention to suppress the *Ae. aegypti* population and reduce the risk of exotic disease transmission. Importantly, genetic analysis revealed a homogenous cluster and small effective population vulnerable to elimination. By 2015, the population had expanded throughout the centre of the town. In response, a collaboration between research agencies and local stakeholders activated a council initiative in 2016 that included extensive community engagement, enhanced entomologic surveillance and vector control activities including the targeting of key containers such as unsealed rainwater tanks. Here we describe a model of the public health intervention which successfully reduced the *Ae. aegypti* population below detection thresholds, using source reduction,

insecticides and novel, intensive genetic surveillance methods. This outcome has important implications for future elimination work in small towns in vulnerable regions and reinforces the longstanding benefits of a partnership model for public health-based interventions for invasive urban mosquito species.

Introduction

Aedes aegypti (Linnaeus) (Diptera: Culicidae) is the primary vector of dengue fever, chikungunya and Zika on mainland Australia. Globally, *Ae. aegypti* is responsible for the rapid re-emergence and spread of viral diseases over the past 40 years (1). Currently, the number of annual global dengue cases is estimated to be 390 million (2), while the Zika epidemic in South America during 2015 was largely attributed to this species (3). *Aedes aegypti* exhibits several characteristics which make it one of the most invasive of all mosquito species. These include a lifecycle highly adapted to urban environments including; a penchant for human blood with multiple feedings per gonotrophic cycle, desiccation resistant eggs that can survive dry and winter conditions, and the utilization of domestic containers as larval habitat (4). Historically, these traits have enabled the species to spread widely within Australia, causing epidemics of dengue fever which have shaped public health policy (5).

Aedes aegypti is postulated to have been introduced into Australia around the time of British colonization, primarily through water storage on large sailing ships (6). By the early 1900s it had spread throughout the eastern seaboard as far south as the Victorian border (7). During the early part of the 20th century, *Ae. aegypti* was responsible for large epidemics of dengue fever in Queensland, with some affecting up to 90% of populations in major urban centres such as Brisbane (7). With a rapid increase in dengue prevalence worldwide since the 1980s, Queensland observed a resurgence of the disease in north Queensland (8). The artificial introduction of the *Wolbachia* symbiont into *Ae. aegypti* populations in 2010 (9) has subsequently reduced the threat of autochthonous dengue outbreaks in northern Queensland (10).

Traditional *Ae. aegypti* management focuses on the removal of larval habitat and the application of insecticides to either control adults or larvae (11). In Australia, the distribution of *Ae. aegypti* has retracted into central Queensland following the adoption of reticulated water and intensive post-World War 2 public health interventions that removed rainwater tanks (a permanent source of larval habitat)(5, 12). Health authorities targeting larval habitat in the state's capital, Brisbane, led to the elimination of *Ae. aegypti* around 1957 and cessation of local dengue transmission since 1947 (5). More recently, the elimination of incursions of *Ae. aegypti* populations from Queensland into the Northern Territory (Tennant Creek and Groote Eylandt) were undertaken through effective community education, larval source reduction and targeted insecticide treatments and monitoring programs (13, 14). These same processes form the core strategy developed by the Queensland government to manage dengue transmission (11).

Central and southern Queensland regions remain vulnerable to exotic disease outbreaks where the wild-type *Ae. aegypti* (no *Wolbachia* infection) vector is abundant and interacts with infective travellers. During mid-2019, Rockhampton recorded the first dengue outbreak in over 60 years (15, 16). It is unknown whether areas outside north Queensland are suitable for *Wolbachia* introduction (17, 18). Thus, traditional methods of mosquito control remain the strategy to reduce the risk of disease transmission by health authorities in southern Queensland regions (11). South East Queensland (SEQ) is highly vulnerable to the establishment of *Ae. aegypti* (19) and receives a high proportion of the annual numbers of viraemic travellers to Queensland (20). Developing regional capacity and capability alongside innovative strategies to eliminate invasive mosquito populations is particularly important when minimizing the risk of exotic disease transmission in large urban centres.

In 2011 *Ae. aegypti* was re-discovered in the small town of Gin Gin after 25 years through house-to-house surveys by state health authorities. The species was not identified in small surveys in 1996 and 2006 (Brian Montgomery, Queensland Health, pers. comm.). Here we provide a public health perspective and document the extensive and enhanced entomological surveillance and control

activities required to eliminate *Ae. aegypti* from Gin Gin that serve as a calibration exercise for similar or larger-scale interventions.

Methods

Chronology of intervention and the various annual activities are described below.

Summer 2011-2012. Initial Detection, Surveillance, Control and Community Engagement

Gin Gin (24.9908° S, 151.9500° E) is a small town (1,053 population)(21) in the Wide Bay Burnett region of Queensland, Australia, located on a major highway into the state capital of Brisbane (Figure 1). In Queensland, local governments are responsible for monitoring and enforcing the *Public Health Act 2005* (22) and *Public Health Regulation 2005* (23). Following the detection of *Ae. aegypti* in Gin Gin during 2011, a report was provided to the Bundaberg Regional Council (BRC) by Queensland Health. Recommendations to prevent the regional spread of the species included:

1) Elimination of the Gin Gin *Ae. aegypti* population to reduce the risk of dengue transmission (from viraemic travellers).
2) Surveying premises in which *Ae. aegypti* were detected (at least once a month for five months during summer), to determine whether *Ae. aegypti* was still present.
3) Surveying premises adjacent to those where *Ae. aegypti* were present to ensure these were also free of the species.

BRC conducted routine adult surveillance at and around the premises where *Ae. aegypti* was first collected (Figure 2A). Oviposition traps (ovitraps) and a network of BG Sentinel traps (24)(BGS; Biogents GmbH, Regensburg, Germany) for adults were set at key premises and collected weekly between the 4th and 11th of May and the 4th of May and 25th of October 2011, respectively. A population suppression program was implemented with BRC the lead agency and technical support provided by Queensland Health. Larval surveillance and control was undertaken over a two week

period (30th January to 17th February 2012). Council staff were trained in house-to-house inspection methodologies and larval surveillance techniques prior to an extensive larval

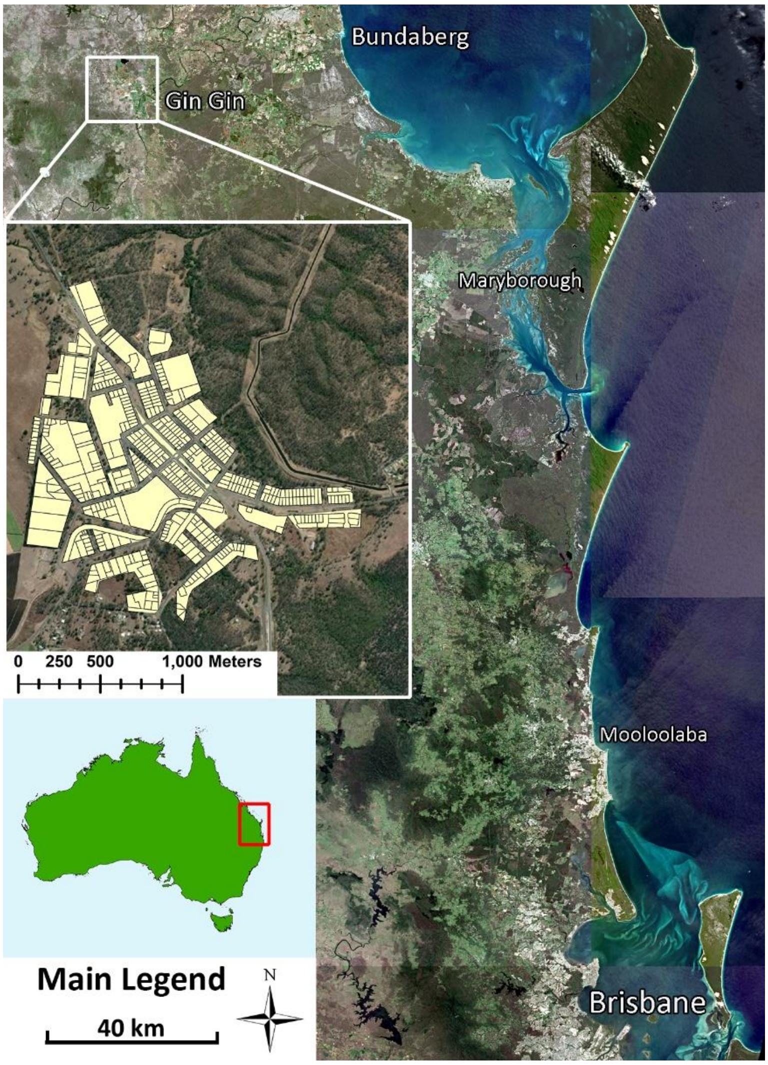

Figure 1. Location of the town of Gin Gin relative to Brisbane and South East Queensland. Location within Australia (bottom left) and Gin Gin town layout including scale (middle left). Map Source: Base Layer assembled from the Open Access Copernicus Australasia Regional Data Hub (38) and Australian map and residential features digitized from public domain cadastre data (37) in ArcGIS.

survey (480 premises). At each premises, verbal consent was obtained to conduct an inspection and if nobody was present the property was re-visited. Premises were scored by the Premise Condition Index (PCI)(25) and all natural and artificial containers holding water were checked for mosquito larvae. A sub-sample (6 to 12 larvae) from each positive larval habitat was placed into 80% ethanol for

species identification by microscopy. Adult surveillance was undertaken by BGS traps set fortnightly (10 premises, 22nd March – 14th May 2012) and ovitraps set weekly (14 premises; 21st March – 22nd May 2012).

Legal mechanisms in Queensland were activated to enable this intervention. Firstly, an 'Authorised Inspection Program' was implemented under the *Local Government Act 2009* to grant powers of entry to yards (not inside houses) by authorised officers without a resident's consent. Secondly, all chemical treatment was consistent with label recommendations, conducted by or supervised by a licensed Pest Management Technician (PMT) and a *Pest Control Advice* (PCA) provided for each premises when treatment occurred. Larval control activities consisted of the removal and/or insecticide treatment of containers that contain or have the potential to contain *Ae. aegypti*. Prolink Pellets® containing the insect growth regulator (*S*)-methoprene were applied to containers that were either; hard-to-inspect, or were large or could not be emptied or removed (e.g. drain sumps, drums, tyres, tree holes). Prolink ProSand® was applied to leaf axils of bromeliads that retain water. To prevent the emergence of adult mosquitoes from large permanent water sources, Prolink XR Briquets® were used in damaged rainwater tanks or if their screens had been removed. For adult control, two lethal ovitraps (26) were set at each of the positive premises.

As part of the 2012 elimination strategy, a community engagement plan was established through targeted awareness campaigns and community engagement strategies including:

- a 'Survey to eliminate *Aedes aegypti* to reduce a public health risk' fact sheet,
- a media release on the 'Gin Gin *Ae. aegypti* elimination program',
- letter for premises that were positive for *Ae. aegypti*,
- letter for premises within a 100 m radius of premises that were positive for *Ae. aegypti* and,
- an information sheet regarding prevention and control of mosquito breeding in the yard.

A full description of 2012 surveillance, control and community engagement methods is documented in S1 Appendix.

2012-2013. Genetic Assessment

A population genetics study was undertaken by BRC to understand the likelihood of *Ae. aegypti* population elimination following removal of the extant population. Samples of larvae (n=39) collected from containers during house-to-house surveys (April 2013) were sent to University of Melbourne (Pest and Environmental Adaptation Research Group) and assessed at the genetic level for population structure using neutral microsatellite markers and compared with other published data on samples from central Queensland (27). Allelic richness calculated from 15 individuals to match the size of the smallest population in the central Queensland study)(A), gene diversity (He), pairwise F_{ST} and inbreeding coefficient (F_{IS}) were estimated using FSTAT 2.9.3.2. Mean effective population size (Ne) was estimated using ONeSamp 1.2. A Bayesian analysis to estimate the number of populations within the sample data was made using STRUCTURE (Version 2) (28). A burn-in length of 100 000 was chosen followed by 250 000 iterations and the simulation was run using the admixture model with allele frequencies uncorrelated among populations. The number of populations within the data (*K*) is estimated by checking the fit of the model for a range of *K* values. *K* values of 1 to 8 were tested with five runs for each value of *K*. We used the method of Evanno *et al.* (29) to estimate the true *K* as applied in STRUCTURE Harvester.

2013-2014. Surveillance Activities

A larval and adult trapping survey was undertaken by Queensland Health (February to April 2014). House-to-house surveys were undertaken at 73 premises, and Gravid *Aedes* Traps (GAT)(30) were placed within six of these for 13 days, serviced after seven days.

2014-2015. Surveillance Activities

A rainwater tank survey on eight rainwater tanks in central Gin Gin premises was performed in December 2014. Tank compliance and a larval sample was collected using the methods of Knox *et al.* (31). Three ovitraps were set around unsealed rainwater tanks for four weeks.

An oviposition study (February - mid-April 2015) was undertaken at nine premises for ten weeks. Four ovitraps and a single GAT containing aged rainwater, a lucerne pellet (32) and water-proof sandpaper (80 grit) used as an oviposition substrate, were placed within the yard of each premises. Traps were serviced fortnightly, eggs counted, and larvae reared to fourth instar for identification to species via microscopy (33).

2015-2016. Surveillance, Experiment, Traditional Control and Community Engagement

A mark-release-recapture (MRR) study within Gin Gin was undertaken in 2016 to understand the distribution and movement of *Ae. aegypti* (34). Forty premises and 58 rainwater tanks were surveyed with the adult trapping methodology and rainwater tank non-compliance survey documented in Trewin *et al.* (34). As part of this study, extensive efforts were made to engage and educate the local community on the risk of *Ae. aegypti* breeding on their premises (S2 Appendix). Community engagement included the formation of a community reference group, town hall meeting, educational flyers and media activities. Homeowners were encouraged to clean-up surplus containers from yards and seal rainwater tanks. Prior to the MRR study, all non-compliant rainwater tanks as defined by regulatory standards (e.g. mesh size apertures to prevent entry or egress of mosquitoes) were sealed with new mesh screens or silicone in rust-related holes. Tanks unable to be sealed were treated with residual insecticides (Prolink XRBriquets®) and residents were encouraged to decommission high risk tanks. Traditional mosquito control was undertaken as part of the risk mitigation strategy including source reduction, residual insecticide treatments of Prolink ProSand® in bromeliads, and Prolink XRBriquets® in larger containers. Indoor residual spraying (35) was offered to residents once the experiment was complete (with two residents and one business opting to have their premises treated). Residents were compensated for their participation through an inexpensive voucher system for redeeming local produce. For 2016 community engagement and risk management and mosquito population suppression plans see S2 Appendix, S3 Appendix and S4 Appendix, respectively.

2017-2018. Surveillance Activities

Records for surveillance by BRC during 2016-2017 were unavailable for analyses. During summer 2017-2018 adult surveys using BGS traps for five weeks across five premises in late summer and classified adults to species by microscopy.

2018-2019. Surveillance Activities

As part of a larger regional population genetics survey, BGS and ovitrapping was undertaken in Gin Gin for ten weeks (February until May 2018), in six houses previously positive to *Ae. aegypti*. A single BGS trap and four ovitraps were placed in the yard of each house and serviced weekly. Concurrent BGS surveillance was undertaken by BRC (five weeks across five premises) in late summer. Larvae (reared to 4th instar) and adults were identified to species via microscopy (33).

2019-2020. Presence-absence surveillance: Rapid Surveillance for Vector Presence Survey

To interrogate the detection threshold indicated by negative records from the previous three summers, a highly sensitive *Ae. aegypti* survey was conducted using an innovative method that links ovitrap samples to molecular diagnostics. Rapid Surveillance for Vector Presence (RSVP) (36) can rapidly detect *Ae. aegypti* nucleic acids by using real-time reverse transcription polymerase chain reaction (RT-PCR) to screen large amounts of genetic material. Large volumes of endemic species can be processed by aggregating egg samples in cohorts (<5000 eggs) that typically are sourced from multiple ovitraps. The sensitivity of RSVP facilitates the efficiency of a regional presence-absence survey of target invasive species over large spatial and temporal scales, particularly when they are expected to be absent or in very low numbers. Since 2017, RSVP has been offered to regional councils in and near SEQ by Queensland Health on a seasonal basis. Premises across the town were selected to ensure all high-risk residential blocks within Gin Gin were sampled for the presence of *Ae. aegypti*. Twenty-one premises were surveyed with a single ovitrap placed within the yard and eggs collected fortnightly for two periods of four weeks (total 8 weeks from February until March 2020).

Mapping

Trapping and larval surveillance results are mapped at the block scale, the spatial unit under which *Ae. aegypti* is optimally targeted due to limited dispersal abilities (34), while also preserving the privacy of individual premises where surveillance was undertaken. All shapefile maps were digitized in ArcGIS by outlining residential features (blocks, roads, highways) and then overlaying public domain cadastre data (37). Base layer imagery of South East Queensland region sourced from the open access Copernicus Australasian Regional Data Hub (38). Trap days is a quantitative measure of the number of traps placed in the environment multiplied by the number of days present when the population was surveyed (39) over a summer season (November until May).

Results

2011-2012. Initial Detection and Elimination Strategy

Aedes aegypti was first detected in a single property in central Gin Gin by a routine Queensland Health house-to-house survey in 2011 (Figure 2A). Mosquito surveillance and suppression activities subsequent to detection revealed *Ae. aegypti* in 2.3% (11/473) of premises throughout central Gin Gin (Figure 2B), representing a modest increase from the 1986 detections (3 premises). During larval surveys, a total of 5,035 wet and 724 dry containers were observed, an average of 12 larval habitat sites per premises. Mosquitoes were present in approximately 40% and 11% of all premises and wet containers, respectively (Table 2). The most prevalent container category positive for *Ae. aegypti* (35%) were garden accoutrements such as plant pots and saucers, birdbaths, buckets and striking pots (S1 Appendix Table 5). Three hundred and forty-seven rainwater tanks (73.4% of premises inspected) were recorded during the survey, with most tanks containing water. Due to difficulty of access, not all tanks were inspected or sampled. Four tanks were positive for mosquito larvae and one was positive for *Ae. aegypti* larvae. Premises previously positive for *Ae. aegypti* remained positive in one of the 14 ovitrap locations and one of three BGS trap locations over the same ten-week period.

Table 1. Prevalence of water bearing containers and number with *Aedes aegypti* juvenile stages in Gin Gin, Australia. Collected during town-wide surveillance activities summer 2011-12.

Category	Total (%)	Wet (%)	***Ae. aegypti*** **Present (%)**
Garden Accoutrement	1,796 (31.2)	1,408 (28.0)	6 (35.3)
Discarded Household Item	460 (8.0)	409 (8.1)	4 (23.5)
Domestic Use Container	259 (4.5)	246 (4.9)	4 (23.5)
Recreational Item	63 (1.1)	62 (1.2)	1 (5.9)
Water Storage	162 (2.8)	157 (3.1)	1 (5.9)
Rubbish	200 (3.5)	159 (3.2)	0
Building Fixture	224 (3.9)	205 (4.1)	0
Natural Habitat	2,248 (39.0	2,044 (40.6)	0
Total	5,759	5,035	17

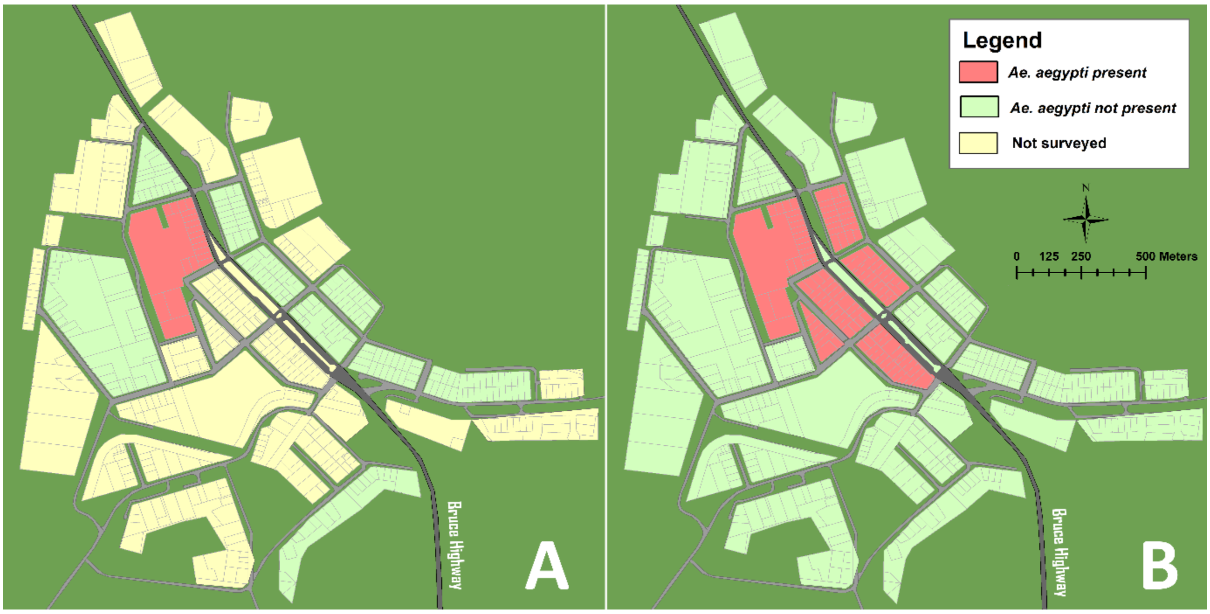

Figure 2. Detection block 2011 (A) and town-wide larval surveys 2012 (B) of *Aedes aegypti* in Gin Gin. Red indicates blocks where *Aedes aegypti* were detected, green where no *Ae. aegypti* were found to be present, and yellow blocks were not surveyed.

2012-2013. Surveys and Genetic Assessment

Aedes aegypti was present across six blocks in central Gin Gin, collected from yard containers and one rainwater tank (S5 Appendix, S6 Appendix Fig 1). The thirty-nine samples collected showed the lowest degree of allelic richness (i.e. low number of microsatellite alleles adjusted for sample size) and lowest gene diversity of the samples tested, which included a range of locations in central and northern Queensland (Table 3). The inbreeding coefficient for the Gin Gin sample was moderate, but significant (Table 3). Pairwise FST estimates between sample localities revealed significant population differentiation between the samples of *Ae. aegypti* from Gin Gin and all other localities (Table 4). The only samples not significantly differentiated from each other were from Gordonvale and Yorkeys Knob.

Effective population size in Gin Gin, estimated by ONeSamp, was small and similar to most samples from central Queensland (Table 2; mean =19.21, median =19.15, lower 95% CL =13.58, upper 95% CL =28.04). STRUCTURE analysis gave an estimate of six genetic clusters (K) within the complete dataset from Queensland, using both the highest log probability of the data and the ΔK method (Evanno *et al*. (29)) (S5 Appendix Table 2). For K = 6, all locations showed some degree of admixture, but for Gin Gin, admixture was minimal (S5 Appendix Fig 1). Yorkeys Knob and Gordonvale from north Queensland were grouped together; Longreach, Bluff, Duaringa and Emerald were separate clusters, while other locations showed a high degree of admixture (S5 Fig 1).

Table 3. Genetic diversity over seven microsatellite loci for *Aedes aegypti* from eleven locations in Queensland, Australia. Results grouped in regions (north Queensland - *NQL*, and central Queensland - *CQL*) with: sample size (N), allelic richness calculated from 15 individuals (A), gene diversity (He), inbreeding coefficient (F_{IS}), mean effective population size (Ne) and 95% confidence limits (in parentheses) (Table modified from Rašić *et al.* 2014 to include data from Gin Gin).

Name	Code	Latitude	Longitude	N	A	He	F_{IS}	Ne

North QLD								
Yorkeys Knob	1	-16.8094	145.7226	30	3.38	0.544	0.125	43 (22-146)
Gordonvale	2	-17.0966	145.7787	28	3.15	0.494	0.164	
Ingham	3	-18.6533	146.1604	15	3.00	0.519	0.083	15 (10-37)
Central QLD								
Rockhampton	4	-23.3795	150.4995	30	3.13	0.479	0.134	33 (19-101)
Mt Morgan	5	-23.6449	150.3889	34	2.75	0.442	0.108	19 (10-57)
Duaringa	6	-23.7110	149.6710	29	3.19	0.457	0.079	24 (14-72)
Bluff	7	-23.5786	149.0703	32	2.78	0.477	0.054	25 (15-90)
Emerald	8	-23.5162	148.1610	28	3.23	0.426	0.054	65 (33-282)
Capella	9	-23.0837	148.0245	21	2.98	0.461	-0.078	22 (14-63)
Longreach	10	-23.4433	144.2509	28	3.07	0.443	-0.063	27 (17-105)
Gin Gin	11	-24.98946	151.9500	39	2.51	0.329	0.093	19 (14-28)

Table 4. Pairwise FST estimates for eleven samples of *Aedes aegypti* from Queensland, Australia. Bold indicates no significant differentiation (Table modified from Rašić *et al*. 2014 to include data from Gin Gin).

	**1**	**2**	**3**	**4**	**5**	**6**	**7**	**8**	**9**	**10**	**11**
**1 Yorkeys Knob**	0										
**2 Gordonvale**	**0.0126**	0									
**3 Ingham**	0.0420	0.0507	0								
**4 Rockhampton**	0.0458	0.0272	0.0831	0							
**5 Mt Morgan**	0.0786	0.0789	0.1191	0.0283	0						
**6 Duaringa**	0.1117	0.1012	0.1589	0.0423	0.0499	0					
**7 Bluff**	0.0619	0.0759	0.0683	0.0871	0.1174	0.1541	0				
**8 Emerald**	0.0548	0.0734	0.0972	0.0527	0.0616	0.1093	0.1333	0			
**9 Capella**	0.1131	0.1263	0.1172	0.0910	0.1028	0.0611	0.0970	0.1533	0		
**10 Longreach**	0.0649	0.0708	0.1277	0.0567	0.0651	0.0573	0.1599	0.0735	0.1215	0	

**11 Gin Gin**	0.0779	0.1134	0.1785	0.1008	0.1455	0.1588	0.1695	0.1119	0.1691	0.1130	0

Summary of Surveillance Activities 2013-2020.

Six positive premises were detected in 2013-2014 (five from larval surveys and from a GAT at a separate premises, across five residential blocks in north-eastern Gin Gin (S6 Appendix Fig 2). Six of eight rainwater tanks inspected were non-compliant with regulations, but not sampled for *Ae. aegypti*.

Larval surveys (2014-2015) suggest *Ae. aegypti* distribution had expanded southward in residential blocks, with five of 10 positive (S6 Appendix Fig 3). The most extensive trapping effort (applying GAT and ovitraps) was undertaken during this season, consisting of 3,150 trap days over a ten-week period (Table 5). During this survey, all non-compliant rainwater tanks were positive (three of three) for *Ae. aegypti*, while three compliant tanks were negative (Table 5). Surveillance (2015-2016) that included a rainwater tank survey for compliance and background *Ae. aegypti* population monitoring during a mark, release, recapture (MRR) experiment is documented in Trewin *et al.* (34). Wild (unmarked) *Ae. aegypti* were collected in twelve of 26 premises (46%), while four of ten rainwater tanks sampled were positive (S6 Appendix Fig 4).

Surveillance activities for 2016-2017 summer season are unavailable, however, this was the first season that BRC reported *Ae. aegypti* to be absent from Gin Gin. Likewise, a five-week BRC survey with five BGS traps in five premises during the 2017-2018 summer season revealed *Ae. aegypti* to be absent (S6 Appendix Fig 5). During the 2018-2019 season both the BRC and the Commonwealth Scientific and Industrial Research Organisation (CSIRO) undertook trapping surveys at ten premises over a ten-week period, representing 1,365 trap days with both BGS and ovitraps, with no *Ae. aegypti* detected (Table 5, S6 Appendix Fig 6).

Enhanced RSVP ovitrap surveillance (2019-2020) did not detect *Ae. aegypti* in any of the blocks where *Ae. aegypti* had been present previously (S6 Appendix Fig 7). A total of 21 premises were surveyed across 13 different blocks for a total of 1,176 trap days across eight weeks (Table 5). A summary of

blocks positive to *Ae. aegypti* previous to 2017 suggest the species distribution was primarily in central areas within the town (Figure 4A), while surveillance effort of 3,591 trap days during the period from 2017 to 2020 suggests the species is no longer present in high-risk central blocks (Table 5, Fig 4B).

Table 5. Summary results of *Aedes aegypti* surveillance activities in the town of Gin Gin, Australia. Trap days indicate the number of traps placed in the town multiplied by the number of days each sampled over a summer season (November-May).

Summer Season	Survey Type	Premises Surveyed	Positive Premises (%)	Survey Period (days)	Trap Days	Positive Traps (%)
2012-2013	Larval	na	14 (na)	2	na	na
2013-2014	Larval	73	5 (7)	2	na	na
	GAT	6	1 (17)	13	78	1 (17)
2014-2015	Ovitrap	9	4 (44)	70	2,520	6 (3)
	GAT	9	2 (22)	70	630	5 (11)
	Rainwater Tank	7	3 (43)	70	na	na
2015-2016	BG Trap	26	12 (46)	13	455	17 (49)
	GAT	26	2 (8)	13	910	10 (14)
	Rainwater Tank	10	4 (40)	1	na	na
2016-2017	Data Unavailable					
2017-2018	BG Trap	5	0 (0)	35	175	0 (0)
2018-2019	BG Trap	10	0 (0)	105	560	0 (0)
	Ovitrap	6	0 (0)	70	1,680	0 (0)
2019-2020	Ovitrap (RSVP)	21	0 (0)	56	1,176	0 (0)

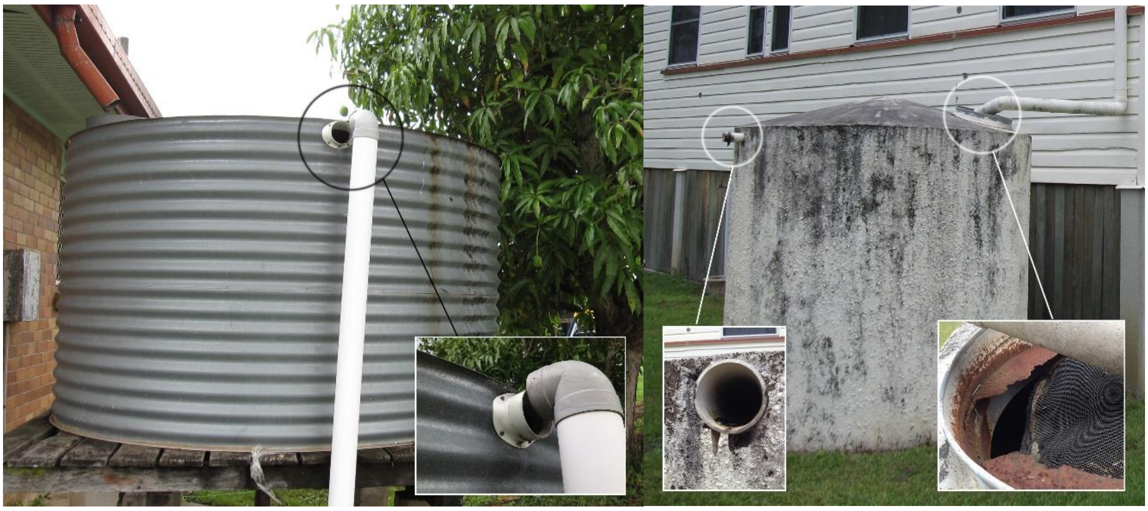

Figure 3. Non-compliant rainwater tanks (with anti-mosquito regulations) identified in Gin Gin containing *Aedes aegypti*. Inserts show detailed views of exposed overflows and rusted inflow sieves.

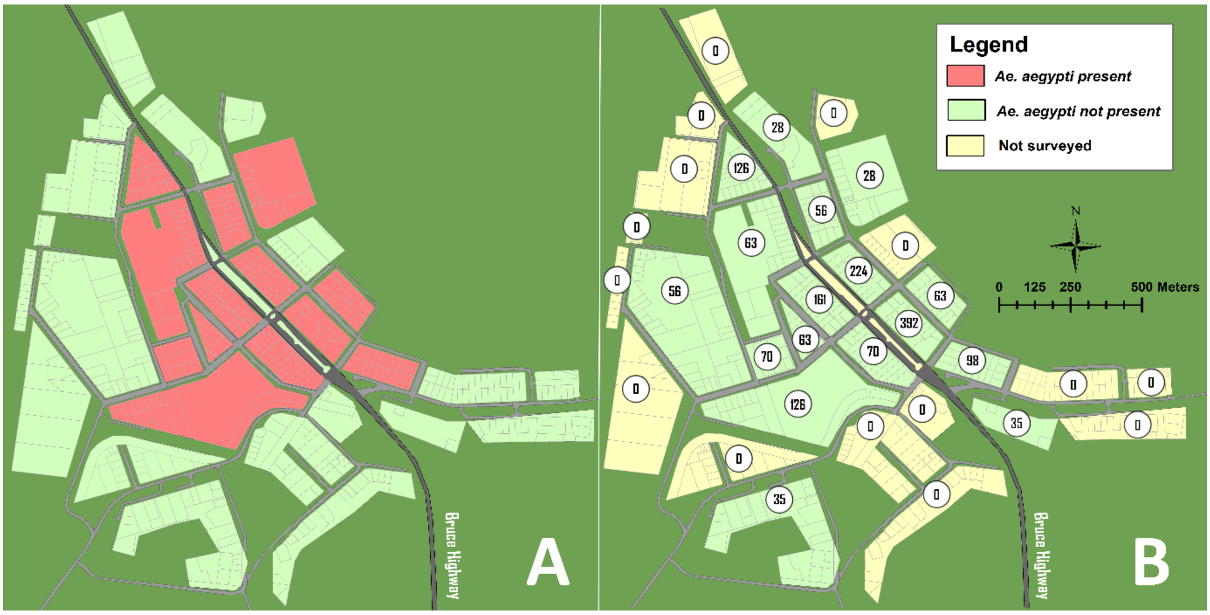

Figure 4. Blocks surveyed for *Aedes aegypti* 2011-2015 (A) and trapping 2016-2020 (B) in Gin Gin, Australia. Circled numbers represent the cumulative number of trap days across the town form 2016-2020 suggesting the species is no longer present (B).

Discussion

In Australia, the prevention of exotic vector-borne disease is a public health matter of national importance. A key component to understand disease transmission risk is access to data of the current distribution and abundance of vector species within different spatio-temporal scales, that range from local contact case addresses, larger environs of town or city and regional perspectives. Additionally, contemporaneous surveillance in regions that are vulnerable to stochastic invasion by urban vectors is required to enable the timely triggering of eradication campaigns as a strategy to avoid scenarios where cryptic outbreaks result from the belated recognition of covert incursions by vectors. However, the logistical challenges to obtain these data and perform eradication protocols is significant. In 2011, *Ae. aegypti* was re-detected in Gin Gin, a small regional town on a major highway into SEQ (a region which contains ~70% of Queensland's population). Infestations in towns on the margins of SEQ, particularly those that are also located on major transport pathways, increase the risk that *Ae. aegypti* could re-invade major population centres such as Brisbane (150 km further south) and transmit large epidemics of dengue (7). Gin Gin provides a case study of the sustained and concerted public health effort, involving both traditional mosquito control and innovative entomological surveillance ~~during the period~~ 2012-2020, that is required to obtain confidence that an *Ae. aegypti* population was suppressed below the level of detection in a small town. This important public health outcome demonstrates that traditional mosquito control is effective at suppressing and potentially eliminating *Ae. aegypti* populations in small towns. However, it should also serve as a warning that traditional methods may not be sustainable for larger towns and cities that will intensify both the spatial (heterogeneous distributions resulting from low dispersal behaviours) and temporal challenges (drought-resistant eggs) for surveillance sensitivity. This will be particularly difficult without investment in national capacity and capability to perform large scale interventions. The logistical resources and costs to scale our model to large urban areas (40) is significant and suggest strategic planning (20) to incursions should embed genetic analyses and additional innovative measures within

routine entomologic surveillance and emergency responses, and include *Wolbachia* or emergent technologies that are not insecticide based.

Managing the Gin Gin detection benefited from health authorities and scientists partnering to conduct regular entomological surveillance and control activities. During summer 2015/2016, the informal technical advisory group had oversight of key mosquito control activities which integrated source reduction (sealed rainwater tanks) and treatment of larval habitat with residual insecticides in the central business and residential areas of the town. This effectively suppressed the population to levels below detection thresholds by traditional surveillance methods. By late summer 2019/2020 and after four years of surveillance activities, *Ae. aegypti* was not detected in Gin Gin. The 2015/2016 intervention was the culmination of sustained and concerted effort by public health authorities and the community to destabilize the *Ae. aegypti* population. This effort included several local initiatives:

1) Effective engagement with the local community, which were highly supportive of mosquito surveillance activities, and ensured ongoing compliance with health authorities;
2) Ongoing surveillance that identified key rainwater tanks acting as major urban mosquito population sources; and
3) Consistent pressure/focus from local government that identified rainwater tanks non-compliant with mosquito regulations were drained, sealed or removed which removed egg banks and ensured larval habitat was unavailable during periods of low rainfall.

Aedes aegypti was initially re-detected in a single block in northern Gin Gin during a routine larval survey. Interestingly, two similar surveys had not previously identified the species in the town. The 2011 detection suggests the population may have persisted at very low levels or been recently re-introduced. Such uncertainty highlights the logistical challenges of traditional house-to-house, presence-absence surveillance for urban mosquitoes that can persist for extended periods as drought-resistant eggs. This species exhibits low movement over a lifetime (<200 m), however, *Ae. aegypti* and other anthropophilic species utilise human-mediated transportation thereby facilitating long-distance

dispersal (41). This long-distance dispersal may be one factor that contributed to the first detection of the species within the northern area of the town. Several reasons could be hypothesised for the 2011 re-detection of the species. The positive residential block contains the local showground which hosts a constant flow of travellers who overnight in campervans. There is also a large commercial trucking stop at the northern end of Gin Gin, two blocks from the detection where large numbers of trucks stay overnight after travelling for extended periods from areas where *Ae. aegypti* is abundant. Mosquitoes may have entered a campervan or truck freight and 'hitchhiked' from northern or central Queensland to Gin Gin. Alternatively, the detection is a remnant historical population last detected in routine house-to-house surveys in 1986. The determination of point-of-origin requires access to high resolution, genomic sequencing techniques and a separate analysis is currently being undertaken on *Ae. aegypti* populations in the region.

A key result of the genetic analysis indicated that the *Ae. aegypti* within Gin Gin formed a homogeneous cluster with a small effective population size. The resolution of the analysis did not differentiate whether this population was newly established in 2011 or a relict population. However, the result concluded that the population is vulnerable to elimination measures given the very low level of genetic admixture, low effective population size and level of inbreeding. Furthermore, the low genetic diversity and degree of differentiation observed between the Gin Gin *Ae. aegypti* population and other Queensland samples suggest a low likelihood of reinvasion from central Queensland. These findings suggest that all new incursion events (e.g. Tennant Creek invasion of 2021) should be genetically analysed to determine whether *Ae. aegypti* genetic profiles are characterised as invasive or not. Our evidence suggests that the Gin Gin *Ae. aegypti* population was vulnerable to elimination measures. Furthermore, this genetic catalogue of the *Ae. aegypti* population is important to determine the long-term effectiveness of suppression outcomes. It will provide a definitive answer to whether future detections are from the original population or introduced from separate populations that may or may not be 'invasive'. Characterization of genotypes from all Queensland population centres will inform a point-of-origin assessment, potentially from highly invasive genotypes, within

SEQ and other Australian incursions. Genomic catalogues will also provide a reference point for potential breaches at international first points of entry.

Elimination campaigns that have used traditional forms of urban mosquito control typically involve community education, source reduction, , and residual insecticides have been effective at eliminating *Ae. aegypti* populations in Australia. For example, the first elimination campaign in Australia removed *Ae. aegypti* from Brisbane and surrounding areas during the mid-twentieth century (5) when the city population was much smaller. The species was eliminated via effective anti-mosquito regulations which targeted larval habitat such as unsealed rainwater tanks (5, 12). More recently, the species has been eliminated from the small and isolated communities of Groote Eylandt (13) and Tennant Creek (14, 42) in the Northern Territory. It is likely that Australia's low rainfall contributed to the long-term and permanent suppression of those populations. A novel method of population suppression under low rainfall conditions is the application of *Wolbachia* that exploits the deleterious effects of certain strains (27, 43). This strategy utilizes the loss of desiccation resistance in *Aedes* eggs to eliminate a population over extended dry periods. This 'replace and suppress' strategy would not only prevent dengue transmission, but is likely to be highly effective for suppressing, populations in large urban settings that will otherwise prove difficult logistically to inspect and treat with insecticides, exacerbated by non-treatment of cryptic larval habitat (44). Utilizing the wet-dry seasonal dynamic of Australian landscapes will be important to future campaigns which seek to eliminate populations of container inhabiting vectors like *Ae. aegypti* and *Ae. albopictus*.

In Queensland, the prevention of dengue and controlling vector species is the shared responsibility of both state and local government organisations. Queensland Health has the overall responsibility under the *Public Health Act 2005* for the control of communicable diseases in Queensland, including exotic mosquito-borne diseases such as dengue fever. Provisions within Chapter 2 of the *Public Health Act 2005* provide local governments with the statutory support and powers to undertake mosquito surveillance and control activities (via insecticide treatment) and to prevent and control public health

risks in relation to mosquitoes within residents' premises. This involves the Queensland Health chief executive sanctioning an authorised prevention and control program when an area is likely to contain an infestation of a disease vector such as *Ae. aegypti* or risk of an outbreak of vector-borne disease. For example, unmaintained rainwater tanks can be made to comply with the *Public Health Regulation 2005* and *Public Health Act 2005* by local authorities, so they no longer function as larval habitat for *Ae. aegypti* or other mosquito species. These essential powers were drawn upon during the period 2011-2020 to ensure residents in Gin Gin did not continue to store or removed containers where *Ae. aegypti* was present. Importantly however, the logistical challenge for health authorities to access and eliminate mosquitoes in all homes and businesses during control activities in very large towns and cities using chemical models of elimination is immense. Thus, a central element of urban mosquito control is to raise awareness about the community's role to adopt behaviours that eliminate mosquito breeding at home and in the workplace. Concurrent investment is required to provide baseline monitoring programs that are more representative of the spatio-temporal parameters of urban mosquitoes to establish entomological confidence in a negative result for invasive species. Current use of surveillance programs that increase throughput and sensitivity by the use of molecular diagnostic platforms (RSVP) and that can be linked to citizen science platforms (Mozzie Monitors (45) and Zika Mozzie Seeker (46)) can provide opportunities to further increase sampling frequency and site number will inform a detection threshold.

Establishing and maintaining community support in Gin Gin was essential to the success of urban mosquito control and dengue prevention initiatives. An effective engagement strategy sets objectives and defines the underlying activities that will best meet these objectives and those of the project. Utilizing council and health officers from the local community promoted and encouraged local acceptance, ownership of the project's goals, and facilitated entomologic surveillance and control activities. Trust is an important element of community engagement which must be established and maintained to ensure community support throughout the life of the intervention. The formation of a community reference group during 2015-2016, was essential to building trust during the intervention

when rainwater tanks were sealed, and insecticides utilized for suppression. A community reference group can provide a social licence to operate and facilitates the transfer of information from scientists or health authorities to the community or opportunities for community concerns to be voiced. Efforts to ensure a comprehensive engagement strategy can foster increased community acceptance, provide local support for activities and even some level of ownership as it promotes both enthusiasm within the community and adherence to personal behaviours. Effective acts that reduce urban mosquito breeding sites in residential premises and significantly reduce the vulnerability of individuals and community to invasive urban species and associated diseases.

Conclusion

The extensive public health efforts documented here demonstrate that an integration of traditional mosquito control, a small genetically isolated mosquito population and public engagement can eliminate *Ae. aegypti* from a small regional town. Replicating this model for large towns and cities at appropriate spatial-temporal scales that will provide an early warning capability and monitor the efficacy and longevity of suppression activities may prove extremely difficult to sustain without incorporating innovative solutions. Removing vectors from a region is a strategic solution to preventing disease transmission. Re-emergence of dengue in Rockhampton after 60 years is a reminder that wild-type populations of *Ae. aegypti* in Queensland still represent a risk of disease transmission. While SEQ region is currently considered vector-free, this Gin Gin case study demonstrates that there is cause for caution. Detection thresholds are insensitive throughout much of the region and stochastic incursions risks remain via freight connections with established *Ae. aegypti* Queensland populations, and increased interceptions at international First Ports of Entry (airports and seaports). This regional risk is heightened by over 300,000 rainwater tanks installed throughout SEQ which are generally unmonitored by authorities and are approaching the end of their warranty periods. Engaging communities to participate in surveillance (citizen science) may also encourage broader awareness and adoption of personal behaviours that reduce availability of

residential and commercial sites to urban mosquito species that will also reduce regional vulnerability to invasive species and associated risk of exotic diseases, particularly in regions with a high number of viraemic travellers and/or proximal to ports of entry. Application of the Gin Gin model to large towns and urban cities of SEQ and Australia will require significant investment in national capacity and capability. Robust and contemporaneous urban mosquito surveillance programs are required that are expansive and sustainable to provide the level of sensitivity required to provide regional confidence that towns and cities are absent of vectors and sensitive enough to detect incursions (that may be focal for many years) relatively quickly. This capability is enhanced by linking surveillance methods to molecular diagnostic methods to develop genetic reference libraries to define species identification, point-of-origin and insecticide resistance. In turn, this investment will build essential experience and baseline monitoring data that will inform elimination strategies if invasive vectors such as *Ae. aegypti* or *Ae. albopictus* are detected in major Australian cities.

Acknowledgements

We would like to thank all current and past staff at the Bundaberg Regional Council who assisted with mosquito trapping and community engagement. We would like to acknowledge the community of Gin Gin for their enthusiasm and support in all surveillance and control activities undertaken within the town.

Supporting Information

S1 Appendix. Queensland Health Report: Population suppression of Aedes aegypti in Gin Gin, Bundaberg, Queensland. (re distributed through a Creative Commons Attribution 3.0 Australia licence).

S2 Appendix. Community Engagement Plan

S3 Appendix. Risk Management Strategy

S4 Appendix. Mosquito Suppression Plan

S5 Appendix. T1. Samples of *Aedes aegypti* larvae. T2. Number of genetic clusters assigned by STRUCTURE of *Aedes aegypti* from Gin Gin, Queensland. F1. Genetic clusters assigned by STRUCTURE for samples of *Aedes aegypti* from Queensland, Australia.

S6 Appendix. *Aedes aegypti* positive residential blocks in Gin Gin, Queensland F1. 2012-2013, F2. 2013-2014, F3. 2014-2015, F4. 2015-2016, F5. 2017-2018, F6. 2018-2019, F7. 2019-2020.

Data reporting

The datasets supporting the conclusions of this article are included within the article (and its additional files).

Financial Disclosure Statement

Bundaberg Regional Council provided funding for the genetic study. NMEH was supported by NHMRC program grants 1037003 & 1132412.

Competing interests

There are no competing interests, whether they be financial, personal or professional, that have influenced the work.

Human and animal research

Human ethics approval for the 2015-2016 community engagement, mosquito suppression and research was provided by the CSIRO Health and Medical Research Human Research Ethics Committee (Proposal #12/2015) and QIMR Berghofer Human Ethics Committee (#P2054).

References

1. WHO. Global Strategy For Dengue Prevention and Control 2012 to 2020. Geneva, Switzerland: World Health Organization; 2012.
2. Bhatt S, Gething PW, Brady OJ, Messina JP, Farlow AW, Moyes CL, et al. The global distribution and burden of dengue. Nature. 2013;496(7446):504-7.

3. Ferreira-de-Brito A, Ribeiro IP, Miranda RMd, Fernandes RS, Campos SS, Silva KABd, et al. First detection of natural infection of *Aedes aegypti* with Zika virus in Brazil and throughout South America. Mem Inst Oswaldo Cruz. 2016;111:655-8.
4. Christophers S. *Aëdes aegypti* (L.) The Yellow Fever Mosquito: Its Life History Bionomics and Structure: London : The Syndics of the Cambridge University Press, Bentley House, 200, Euston Road, N.W.I.; 1960. xii + 739 pp. p.
5. Trewin BJ, Darbro JM, Jansen CC, Schellhorn NA, Zalucki MP, Hurst TP, et al. The elimination of the dengue vector, *Aedes aegypti*, from Brisbane, Australia: The role of surveillance, larval habitat removal and policy. PLOS Negl Trop Dis. 2017;11(8):e0005848.
6. Maynard AJ. Invasion history and evolution of the Asian tiger mosquito *Aedes albopictus* (Skuse, 1894) and yellow fever mosquito *Aedes aegypti* (Linnaeus, 1762) in the Indo-Pacific. Brisbane, Australia: University of Queensland; 2020.
7. Lumley GF, Taylor FH. Dengue: School of Public Health and Tropical Medicine (University of Sydney); 1943.
8. Kay B, Barker-Hudson P, Stallman N, Wiemers M, Marks E, Holt P, et al. Dengue fever. Reappearance in northern Queensland after 26 years. Med J Aust. 1984;140(5):264-8.
9. Hoffmann AA, Montgomery BL, Popovici J, Iturbe-Ormaetxe I, Johnson PH, Muzzi F, et al. Successful establishment of *Wolbachia* in *Aedes* populations to suppress dengue transmission. Nature. 2011;476(7361):454-7.
10. Ryan PA, Turley AP, Wilson G, Hurst TP, Retzki K, Brown-Kenyon J, et al. Establishment of *w*Mel Wolbachia in *Aedes aegypti* mosquitoes and reduction of local dengue transmission in Cairns and surrounding locations in northern Queensland, Australia. Gates Open Res. 2020;3:1547-.
11. State of Queensland. Queensland Dengue Management Plan 2015-2020. In: Branch CD, editor. 15 Butterfield St, Herston Qld 4006, Fortitude Valley BC 4006: Queensland Health; 2015. p. 62.
12. Russell RC, Currie BJ, Lindsay MD, Mackenzie JS, Ritchie SA, Whelan PI. Dengue and climate change in Australia: predictions for the future should incorporate knowledge from the past. Med J Aust. 2009;190(5):265-8.
13. Whelan PI, Kulbac M, Bowbridge D, Krause V. The eradication of *Aedes aegypti* from Groote Eylandt NT Australia 2006-2008. Queensland Institute of Medical Research, Arbovirus Research in Australia (10). 2009.
14. Whelan PI, Kurucz N, Pettit WJ, Krause V. Elimination of *Aedes aegypti* in northern Australia, 2004–2006. J Vector Ecol. 2020;45(1):118-26.
15. Doherty R. Clinical and epidemiological observations on dengue fever in Queensland, 1954-1955. Med J Aust. 1957;44(22):753.
16. Webb CE. After decades away, dengue returns to central Queensland The Conversation2019 [Available from: https://theconversation.com/after-decades-away-dengue-returns-to-central-queensland-117821.
17. Ross PA, Wiwatanaratanabutr I, Axford JK, White VL, Endersby-Harshman NM, Hoffmann AA. Wolbachia Infections in *Aedes aegypti* Differ Markedly in Their Response to Cyclical Heat Stress. PLoS Pathog. 2017;13(1):e1006006-e.
18. Mancini MV, Ant TH, Herd CS, Gingell DD, Murdochy SM, Mararo E, et al. High temperature cycles result in maternal transmission and dengue infection differences between *Wolbachia* strains in *Aedes aegypti*. bioRxiv. 2020:2020.11.25.397604.
19. Trewin B. Assessing the risk of establishment by the dengue vector, *Aedes aegypti* (L.)(Diptera: Culicidae), through rainwater tanks in Queensland: back to the future. Brisbane: University of Queensland; 2018.
20. Health Q. Framework for Risk Assessment and Response for Incursion of Exotic Mosquitoes into a New Location in Queensland. In: Branch CD, editor. Herston, Queensland: Queensland health; 2020. p. 37.

21. Australian Bureau of Statistics. Regional Population Growth, Australia, 2015-16 2016 [Available from: http://www.abs.gov.au/AUSSTATS/abs@.nsf/DetailsPage/3218.02015-16?OpenDocument.
22. Public Health Act. Public Health Act 2005. Queensland: State of Queensland; 2005.
23. Public Health Act. Public Health Act – Mosquitos and the Public Health Regulation 2005. In: Government TQ, editor. 2005.
24. Williams CR, Long SA, Russell RC, Ritchie SA. Field efficacy of the BG-Sentinel compared with CDC Backpack Aspirators and CO2-baited EVS traps for collection of adult *Aedes aegypti* in Cairns, Queensland, Australia. J Am Mosq Control Assoc. 2006;22(2):296-300.
25. Tun-Lin W, Kay B, Barnes A. The premise condition index: a tool for streamlining surveys of *Aedes aegypti*. Am J Trop Med. Hyg. 1995;53(6):591-4.
26. Williams CR, Ritchie SA, Long SA, Dennison N, Russell RC. Impact of a Bifenthrin-Treated Lethal Ovitrap on *Aedes aegypti* Oviposition and Mortality in North Queensland, Australia. J Med Ent. 2007;44(2):256-62.
27. Rasic G, Endersby NM, Williams C, Hoffmann AA. Using *Wolbachia*-based release for suppression of *Aedes* mosquitoes: insights from genetic data and population simulations. Ecol Appl. 2014;24(5):1226-34.
28. Pritchard JK, Stephens M, Donnelly P. Inference of population structure using multilocus genotype data. Genetics. 2000;155:945-59.
29. Evanno G, Regnaut S, Goudet J. Detecting the number of clusters of individuals using the software STRUCTURE: a simulation study. Mol Ecol. 2005;14:2611-20.
30. Ritchie SA, Buhagiar TS, Townsend M, Hoffmann A, Van Den Hurk AF, McMahon JL, et al. Field validation of the gravid *Aedes* trap (GAT) for collection of *Aedes aegypti* (Diptera: Culicidae). J Med Ent. 2014;51(1):210-9.
31. Knox TB, Yen NT, Nam VS, Gatton ML, Kay BH, Ryan PA. Critical evaluation of quantitative sampling methods for *Aedes aegypti* (Diptera: Culicidae) immatures in water storage containers in Vietnam. J Med Ent. 2007;44(2):192-204.
32. Ritchie SA. Effect of some animal feeds and oviposition substrates on *Aedes* oviposition in ovitraps in Cairns, Australia. J Am Mosq Control Assoc. 2001;17(3):206-8.
33. Marks EN. An atlas of common Queensland mosquitoes: University of Queensland Bookshop; 1967.
34. Trewin BJ, Pagendam DE, Zalucki MP, Darbro JM, Devine GJ, Jansen CC, et al. Urban Landscape Features Influence the Movement and Distribution of the Australian Container-Inhabiting Mosquito Vectors *Aedes aegypti* (Diptera: Culicidae) and *Aedes notoscriptus* (Diptera: Culicidae). J Med Ent. 2020;57(2):443-53.
35. Ritchie S, Hanna J, Hills S, Piispanen J, McBride W, Pyke A, et al. Dengue Control in North Queensland, Australia: Case Recognition and Selective Indoor Residual Spraying. Dengue Bull. 2002;26:7-13.
36. Montgomery BL, Shivas MA, Hall-Mendelin S, Edwards J, Hamilton NA, Jansen CC, et al. Rapid Surveillance for Vector Presence (RSVP): Development of a novel system for detecting *Aedes aegypti* and *Aedes albopictus*. PLOS Negl Trop Dis. 2017;11(3):e0005505.
37. Department of Resources. Cadastral data - Queensland series. In: Government Q, editor. 6.13 ed. Open Data Portal2021.
38. Programme C. Copernicus Australasia Regional Data Hub [Available from: http://www.copernicus.gov.au.
39. Silver JB. Mosquito Ecology: Field Sampling Methods. 3rd Edition ed: Springer; 2008.
40. Darbro J, Halasa Y, Montgomery B, Muller M, Shepard D, Devine G, et al. An Economic Analysis of the Threats Posed by the Establishment of *Aedes albopictus* in Brisbane, Queensland. Ecol Econ. 2017;142:203-13.

41. Damal K, Murrell EG, Juliano SA, Conn JE, Loew SS. Phylogeography of *Aedes aegypti* (Yellow Fever Mosquito) in South Florida: mtDNA Evidence for Human-Aided Dispersal. Am J Trop Med Hyg. 2013;89(3):482-8.
42. Whelan PI, Pettit WJ, Krause V. Dengue mosquito incursion into Tennant Creek, 2011. Dis Cont Bull 2012;19(1):16-21.
43. Ritchie SA, Townsend M, Paton CJ, Callahan AG, Hoffmann AA. Application of *w*MelPop *Wolbachia* Strain to Crash Local Populations of *Aedes aegypti*. PLOS Negl Trop Dis. 2015;9(7):e0003930.
44. Russell B, Kay B, Shipton W. Survival of *Aedes aegypti* (Diptera: Culicidae) eggs in surface and subterranean breeding sites during the northern Queensland dry season. J Med Ent. 2001;38(3):441-5.
45. Braz Sousa L, Fricker SR, Doherty SS, Webb CE, Baldock KL, Williams CR. Citizen science and smartphone e-entomology enables low-cost upscaling of mosquito surveillance. Science of The Total Environment. 2020;704:135349.
46. Ritchie SA, Staunton KM. Reflections from an old Queenslander: can rear and release strategies be the next great era of vector control? Proc Royal Soc B. 2019;286(1905):20190973-.

Click here to access/download
Supporting Information - Compressed/ZIP File Archive
Supplemental.zip
