# Supplementary material for: Extensive public health initiatives drive the elimination of Aedes aegypti (Diptera, Culicidae) from a town in regional Queensland: A case study from Gin Gin, Australia
Source: PLoS Negl Trop Dis. 2022 Apr 8;16(4):e0010243. doi: 10.1371/journal.pntd.0010243 (PMC9020727; doi:10.1371/journal.pntd.0010243)
Supplement: Attachment — Submitted filename: ResponseToReviewers.docx [file pntd.0010243.s008.docx]

Dear PLOS NTD editorial staff and reviewers, below we have provided responses to reviewer comments from both the email that was sent and in-text, with line numbers corresponding to the newly revised manuscript. We hope these responses provide enough detail to assist you with your further review of our manuscript.

**Reviewer 1:**

**In Email Comments:**

**Methods:**

**The article is not a traditional research study, but instead documents an eradication campaign of Ae. aegypti from an area in Australia. The objectives could be made more clear and the methods expanded on. The author references methods described in other papers, but does not include brief abbreviated methods to aid the reader in understanding how the study was done. In my opinion, an improvement of the methods is needed to improve the paper.**

Abstract: We have included further detail that elimination was an objective from the start of the control campaign.

Lines 16-18: “Following the detection, larval habitat and adult control activities were conducted as a public health intervention to eliminate the *Ae. aegypti* population and reduce the risk of exotic disease transmission.”

Methods:

There has been extensive work undertaken over the nine years and a detailed methods section would not allow for a concise manuscript. We note that methods are documented extensively in the supplementary section and other published works referenced in the text. We have added some extra detail to the methods.

Lines 169-171: “Tank compliance and a larval sample was taken from each rainwater tank via the methods of Knox *et al*. (39) where a large sweep net is used to exhaustively sample a large volume of water.”

**Reference to a previously published MRR study seems misplaced in the methods or its relevance was not explained well enough. It's relevance to the presented study needs to be made more clear.**

We have provided more detail on why the MRR study undertaken in 2016 was important to the elimination of the species.

Methods:

Lines 196-198: “A second Ae. aegypti suppression program was undertaken in conjunction with a mark-release-recapture (MRR) study in Gin Gin during 2016 and reduce the probability that released Ae. aegypti females would increase the risk of disease transmission (40).”

Discussion:

Lines 395-400: “The Gin Gin elimination documented here benefited from health authorities and scientists partnering to conduct regular entomological surveillance and control activities. During the second Ae. aegypti suppression program in 2015/2016, the informal technical advisory group had oversight of key mosquito control activities which integrated source reduction (sealed rainwater tanks) and treatment of larval habitat with juvenile hormones (S-methoprene) in the central business and residential areas of the town.”

**Methods on how larval surveillance was conducted/tools that were utilized should be expanded on.**

We note that most of the larval surveillance was undertaken during the elimination when the species was first identified in 2011-2012, as this was the standard process for surveillance by Queensland Health at the time. We are limited in the detail we can provide for all methods, however, larval surveillance during this period are documented in Appendix 1.

We have included some clarification in text:

Lines 96-97: “Larval surveillance and control was undertaken over a two week period (30^th^ January to 17^th^ February 2012) with methods documented in Appendix S1.”

Lines 127-130: “Briefly these methods were; verbal consent was obtained to conduct an inspection at each premises and if nobody was present the property was re-visited. Premises were scored by the Premise Condition Index (PCI) (28) and all natural and artificial containers holding water were categorised into groups of breeding sites via the Barker-Hudson method (29) and checked for the presence of mosquito larvae.”

The remaining larval surveillance was undertaken inside rainwater tanks. We have provided some further clarification:

Lines 185-187: “Surveys include tank compliance with state health regulations (23) and a larval sample was taken from each rainwater tank via the methods of Knox et al. (39) where a large sweep net is used to exhaustively sample a large volume of water.”

**Results:**

**Both the results and discussion would benefit from an additional figure (that could be combined and used for the two separate sections) that has a visual representation of the surveillance activities and results. Because this study was conducted over several years and the surveillance activities changed year to year, it is hard to follow without a visual representation. The same goes for the results. A timeline indicating what activities were conducted when AND when Ae. aegypti were detected and when they weren't would be very useful.**

We hope the two new figures in the main manuscript assist with reviewer with these comments.

Line 360: We have now included a timeline of activities related to surveillance and control from 2011-2020.

Line 3: We have now included Appendix 6 in the main manuscript as Figure 3 as tiled maps of surveillance from 2012-2020.

**Some of the figures in the appendix would be better utilized in the main manuscript to aid in telling the story. For example, the figures in S6, if they could be combined into a figure show a FANTASTIC visual representation of the progression of surveillance and the author's findings. It is wasted as an appendix and would greatly aid a reader in interpreting the written findings of the authors.**

Line 321: We have included Appendix 6 into the main manuscript as Figure 3.

**Conclusions:**

**The conclusions are supported by the presented data. However, in the discussion section, the authors have a great deal of work to do relating their study to the research of others. The discussion is very weakly cited which I see as the greatest weakness of this manuscript. When the authors do reference other studies, it is almost exclusively for other studies conducted in Australia. There is a great deal of literature on eradication campaigns, community engagement studies for mosquito control, etc. This literature was not included and related to the current study. Therefore, the presented study cannot be considered in the context of previously conducted work. The discussion needs substantial improvement in this way.**

We hope the modifications made to the discussion, including further referencing and listed individually below, address this broad comment from the reviewer.

**Summary and General Comments:**

**Overall, it is clear that the authors invested a great deal of effort into this eradication campaign and documenting its progression. It clearly worked and the area is free of Ae. aegypti as a result. Because this was a SUCCESSFUL eradication campaign, communicating the process and findings effectively is all the more important so the methods can be utilized by others wishing to do the same. Additionally, relating this research to others may also help readers understand what successful eradication campaigns have in common and why others failed. With these changes, I believe this will be a very valuable piece of research.**

We hope the modifications made to the discussion, and listed individually below, address this comment from the reviewer.

**In Text Comments:**

**Line 36: It may also be worth mentioning that this is a significant factor in their role as an arbovirus vector as well. It fits nicely in your introduction of why this species is so important.**

We have included a statement linking *Aedes aegypti* as a competent vector of dengue

Lines 34-35: “*Aedes aegypti* exhibits several characteristics which make it one of the most invasive of all mosquito species and a competent vector of dengue.”

**Line 47: Expand on this just a little more in this introduction because it is important to your study. A short addition of how the threat was reduced (population reduction? interaction of Wolbachia and virus?)**

We have included a little extra detail on the mechanism of by which transmission has decreased:

Lines 49-52: “The artificial introduction and replacement of *Ae. aegypti* populations with a *Wolbachia* symbiont in 2010 (10) has subsequently reduced the threat of autochthonous dengue outbreaks in northern Queensland by blocking viral transmission within adult mosquitoes (11).”

**Line 77: Because the methods section is more of a chronology of how the intervention was done, a timeline with major activities would be useful. As I am reading through the methods, I personally would like to have a visual representation of the information to reference.**

Line 360: We have now included a timeline of activities related to surveillance and control from 2011-2020.

**Line 82: In brief, what do these acts and regulations specify?**

We have added further detail to what these acts allow for, however, this is now repeated in the discussion as well.

Lines 86-88: “These acts give local government the power to enter premises without consent for both mosquito surveillance and control, dependent upon the threat to the community (mosquito surveillance or prevention of disease transmission).”

**Line 83: Do you have a reference for this report? Are these points quoted from the report or just a summary?**

This report isn’t available for the public as I was unable to get permission from the authors at QLD Health to include it. The points here are a summary of key recommendations within the report.

**Line 91: Is this as a result of the report?**

This is a recommendation within the report. I have added extra detail:

Line 90: “In order to prevent the regional spread of the species, this report recommend:”

**Line 92: How many? And at what spacing/ density?**

We have provided explicit information about the placement of a single ovitrap and BGS at each positive premises:

Lines 98-103: “One oviposition trap (ovitrap) containing aged rainwater, a lucerne pellet and an oviposition substrate (typically a red material or water-proof sandpaper (80 grit) Appendix S1; 24) and BG Sentinel trap (25)(BGS; Biogents GmbH, Regensburg, Germany) were set at the original positive premise, the surrounding properties and other high risk locations (caravan park, ambulance station, and council depot). Ovitraps were collected weekly between the 4th and 11th of May and BGS traps between the 4th of May and 25th of October 2011.”

**Line 94: Was there a reason the ovitraps were only used for such a short period of time? It often takes multiple weeks from deployment before ovitraps begin tocollect/ detect Aedes. Aslo, please include a reference for the ovitraps used or a brief description if they have not been previously described.**

As this was a council survey, there was no particular reason the ovitraps were deployed for one week. In these regions winter occurs after May and there is generally no adult *Ae. aegypti* activity during this period as it is too cold. Furthermore, BGS traps are generally preferred as they do not require larvae to be reared in areas where the species is not present. BGS traps which are a more effective adult trap, were run for the entire winter period and up until October. We have inserted a reference for the ovitrap.

**Line 119: This is separate from the ovitrap surveillance described above?**

It was a separate round of surveillance the following year. We now refer to this a “second round of adult surveillance”:

Lines 133-135: “A second round of adult surveillance was undertaken by BGS traps set fortnightly (10 premises, 22nd March – 14th May 2012) and ovitraps set weekly (14 premises; 21st March – 22nd May 2012).”

**Line 122: Is there a reference for this?**

Line 137: We have included a reference for this government Act.

**Line 130: Does Prolink ProSand have the same AI as Prolink Pellets? I'm assuming so, but specifying is always beneficial**

Lines 142-147: We have included (*S*)-methoprene to the commercial products to be specific.

**132a: Is adulticide spraying conducted in the area? If not, might be worth mentioning here.**

Adulticide spraying is rarely used in Australia. We have included the reviewers suggestion:

Lines 148-150: “Adulticides are not used in the region for mosquito control, however, to target adult Ae. aegypti two lethal ovitraps (31) treated with Bifenthrin were set at each of the positive premises (S1 Appendix). “

**132b: Include information on active ingredient(s)**

We have included the active ingredient in the lethal ovitraps (see above)

**Line 148: Are there brief methods that can be supplied for this?**

The methods are available through the open access reference (32) provided in the text.

**Line 161a: Were these all within the same regions in Figure 2? Need to specify which blocks received the surveillance activities.**

They were in the same region as Figure 2. We have now included Figure 2 in the Introduction and Figure 5 in the Results if readers would like to cross-reference which blocks were surveyed in 2012-13.

**Line 161b: Manufacturer information needed**

We have included the manufacturer:

Lines 181-182: “Gravid *Aedes* Traps (GAT; Biogents GmbH, Regensburg, Germany)”

**Line 165: Briefly (abbreviated methods)**

We have included a brief description of these methods:

Lines 186-189: “Surveys include tank compliance with state health regulations (23) and a larval sample was taken from each rainwater tank via the methods of Knox et al. (39) where a large sweep net is used to exhaustively sample a large volume of water. Three ovitraps were set around unsealed rainwater tanks for four weeks.”

**Line 169: This can be included in parentheses to improve the readability of this sentence.**

This phrase is no longer present in its previous form.

**Line 173: Methods on how the MRR study was done need to be included. What mosquitoes were used? Lab? Field collected?**

We have briefly included a description of this study’s goals, including the use of Australian vectors Ae. aegypti and Ae. notoscriptus. Readers can follow the reference to the study if they require further detail on the MRR methods deployed. We have included all community engagement and risk management information for the MRR in the current manuscript’s appendices.

Lines 188-190: “During the MRR, locally caught adult Ae. aegypti and Ae. notoscriptus (Skuse) were released to study the movement of vectors between urban landscape features such as rainwater tanks , and recaptured in a network of BGS and GAT traps (40).”

**Line 176: Briefly describe**

We have briefly described the rainwater tank survey. Further details of the MRR are listed above.

Lines 199-200: “In this survey, rainwater tanks were examined for their compliance with state regulations (sealed, with mesh on both exit and entry) (23).”

**Line 177: I caution against using the word breeding as it relates to larval habitats. See Rupp et al. 2006. Of Breeding in the Journal of the American Mosquito Control Association**

We thank the reviewer for their concerns on the use of the word breeding. In this case we believe the term is correctly used as a verb for “to produce”. It does not specifically relate to larval habitat and therefore does not conform to any of the alternate terms recommended by Rupp et al. 2006. We have instead changed the word to presence but believe this does not read as well as the previous word describing the production of individuals.

Lines 203-204: “As part of this study, extensive efforts were made to engage and educate the local community on the risk of Ae. aegypti presence on their premises (S2 Appendix).”

**Line 183: Two words**

We have fixed this name

**Line 186: Two words**

We have fixed this name

**Line 186: With what? A pyrethroid?**

We have included the name of the pyrethroid used in the IRS.

Lines 201-202: “Indoor residual spraying (IRS; 41) with Bifenthrin was offered to residents once the experiment was complete (with two residents and one business opting to have their premises treated).”

**Line 192: Is there any information about the population from 2016-2017? From other studies? If this is covered in results or discussion, than disregard, but even if you don't have any data you collected, referencing other data so it can be interpreted as a whole would be useful.**

In the text your comments are referring to survey for 2017-18. Unfortunately there is no data available for this year from the council. However, the council has told us that surveys during this year did not detect *Ae. aegypti*. If you are interested in the 2016-2017 MRR population estimate data it is available from the MRR paper sited in the manuscript. We provide an estimate of the number of traps used, their positivity in the results of the current manuscript. There is also the number of premises surveyed and their positivity. Please see Table 5 in the results for this data.

**Line 194: Rephrase the latter part of this sentence or break into two sentences to improve readability.**

We have improved the structure of the sentence

Lines 207-209: “During summer 2017-2018 adult surveys using BGS traps for five weeks across five premises. Adults were classified adults to species by microscope and key (26).”

**Line 196: Is this different than the genetic study conducted in 2012-2013?**

This is referring to a different study using genomics, not genetics. We have provided the reference to the preprint of this manuscript and rephrased it to “genomics” to help differentiate.

Lines 224-226: “As part of a larger regional population genomics survey (43), BGS and ovitrapping was undertaken in Gin Gin for ten weeks (February until May 2018), in six houses previously positive to Ae. aegypti. A single BGS trap and four ovitraps were placed in the yard of each house and serviced weekly.”

**Line 216: Please give a range of the size of these blocks.**

We have provided a range on size based on hectares:

Lines 244-247: “Trapping and larval surveillance results are mapped at the block scale (Gin Gin residential blocks range from 0.39 to 15.20 ha), the spatial unit under which *Ae. aegypti* is optimally targeted due to limited dispersal abilities (45), while also preserving the privacy of individual premises where surveillance was undertaken.”

**Line 237: From larval survey? At what time point? Please specify**

We have clarified which larvae survey this sentence refers to and added years in which these were undertaken.

Lines 266-268: “Premises from the initial 2011 larval surveys for *Ae. aegypti* were positive in one of the 14 ovitrap locations and one of three BGS trap locations over the same ten-week period in 2012 (Figure 2).”

**Table 1a: Specify that this is the % of wet containers where Ae. aegypti was present.**

We have specified that the fourth column corresponds to wet container with Ae. aegypti present.

**Table 1b: Are there definitions for these categories somewhere? How does Discarded household item differ from Rubbish?**

We have clarified that these container categories are defined by the Barker-Hudson method.

Lines 270-271: “Container data collected during town-wide surveillance activities summer 2011-12 and classified by the Barker-Hudson method (30).”

**Line 279: Compared to x of x positive in a different year. Expand this to include that information so the reader can get the necessary information without needing to jump around between resources.**

We have now included all years of surveillance in Figure 4 to assist the reader. We now refer to Figure 4 in the text multiple times when referring to surveillance and positive residential blocks.

**Line 284: Briefly, what were the findings? If not your results, maybe it is more relevant to bring this piece up in the discussion**

We now refer to this time as the “second Ae. aegypti suppression program” and include some of the results from the non-compliance survey for reference from Trewin et al 2020.

Lines 324-330: “Surveillance during the second suppression program (2015-2016, Fig 5), that included a rainwater tank survey for compliance and background Ae. aegypti population monitoring, revealed 46.6% of tanks non-compliant with regulatory standards (35). Wild (unmarked) Ae. aegypti were collected in twelve of 26 premises (46%), while four of ten rainwater tanks sampled were positive (Fig 3). Control activities included treating 40 containers and one tank with S-methoprene pellets and briquettes, respectively, with four IRS treatments, 24 non-compliant tanks sealed (Fig 4), and 44 rubbish containers removed (Table 2).”

**Line 313: Remove “suggesting the species is no longer present”**

We have removed this Figure and replaced it with the new tiled map (Figure 3).

**Line 327: This needs to be rephrased. The mosquito can't transmit an epidemic.**

We have removed this phrase.

**Line 336: Do you have any references that support this? Relating your findings to other literature is crucial.**

We have provided references to these statements.

Lines 382-385: “However, lessons learnt since these historical campaigns suggest traditional methods may not be sustainable for larger, modern towns and cities, where introductions will vary both spatially (heterogeneous distributions resulting from low dispersal behaviours) and temporally (drought-resistant eggs) (50, 51).”

**Line 341: Why exclude insecticide based methods? During transmission events, this is arguably one of the most important tools as it kills the potentially infected adult population. I would suggest rephrasing this.**

This manuscript focuses on the elimination of Ae. aegypti populations. Modern insecticide methods have continued to fail to control large establishments of this species and others. In this paragraph we are focused on elimination strategies and how they have failed in the past because traditional methods do not scale very well. We conclude the paragraph with an answer to this quandary by suggesting that emergent technologies need to be considered as an alternative.

Lines 365-368: “The logistical resources and costs to scale our model to large urban areas would be significant (54) and suggests strategic planning for incursions (20) should embed genetic analyses and additional innovative measures within routine entomologic surveillance and emergency non-insecticide-based responses, including Wolbachia or other emergent technologies, should be considered in public health policy.”

**Line 345: Specify larvicides**

We have included more specificity on the insecticide we deployed.

Lines 395-399: “During the second *Ae. aegypti* suppression program in 2015/2016, the informal technical advisory group had oversight of key mosquito control activities which integrated source reduction (sealed rainwater tanks) and treatment of larval habitat with juvenile hormones (S-methoprene) in the central business and residential areas of the town.“

**Line 365: More references are needed to support the point you are trying to make here. There are other studies on dispersal and flight range of Ae. aegypti**

We have included references to support this statement:

Lines 415-417: “This species exhibits low movement over a lifetime (<200 m), however, Ae. aegypti and other anthropophilic species utilise human-mediated transportation thereby facilitating long-distance dispersal (55, 56).”

**Line 367: From this point through the rest of the paragraph, you are severely lacking in references. Other authors have discussed points like this and referencing that literature here is necessary to substantiate your hypotheses on reinvasion.**

We have included a reference where statements require further support. We do not believe it is necessary to reference statements about commercial trucking or campervan movements in Gin Gin and from areas where Ae. aegypti is common. We have provided a link showing anthropophilic mosquitoes can be observed in cars.

Lines 421-425: “There is also a large commercial trucking stop at the northern end of Gin Gin, two blocks from the detection where large numbers of trucks stay overnight after travelling for extended periods from areas where Ae. aegypti is abundant. Adult mosquitoes may have entered a campervan or truck freight and ‘hitchhiked’ from northern or central Queensland to Gin Gin, a trait which is common in anthropophilic mosquitoes (57).”

**Line 376: Same point as above. Have other authors explored something similar? Were there findings** **similar? You absolutely have to incorporate reference to other similar studies into your discussion to drive your points home.**

We have updated this paragraph to include what other authors have found, including adding further references.

Lines 430-446: “Genetic analysis indicated that the Ae. aegypti within Gin Gin formed an homogeneous cluster with a small effective population size. The resolution of the analysis did not establish whether this population was newly founded in 2011 or a relict population, but no first-generation immigrants were identified. The population was deemed vulnerable to eradication with low potential for reinvasion given the very low level of genetic admixture observed as well as the level of inbreeding and the degree of differentiation from the other samples from Queensland. A similar conclusion was reached for several semi-isolated populations in other parts of central Queensland (33). Small effective population size in Ae. aegypti has been observed in other locations around the world and is a favourable attribute for population suppression or replacement of this species (58). Genetic and genomic analysis of any Ae. aegypti incursion (e.g. Tennant Creek invasion of 2021; 13, 14) is important to identify invasion pathways and determine the long-term effectiveness of suppression strategies (56, 59). Such analyses, based on reference populations throughout Queensland, would provide definitive answers to whether future detections in Gin Gin are from the original population or introduced from elsewhere. Characterization of Ae. aegypti genotypes from all Queensland population centres would inform a point-of-origin assessment within SEQ and would be useful for identifying or eliminating sources of other Australian incursions. Genomic databases of Ae. aegypti from southeast Asia and the Indo-Pacific region would also provide a reference point for potential international incursions (59).”

**Line 396: What about elimination campaigns in other parts of the world? What made them successful or unsuccessful? How does that differ from your study and what hurdles were you able to overcome to make your eradication campaign successful.**

We have included further information on elimination campaigns in other parts of the world in paragraph 1 of the Discussion and discussed why they were unsuccessful. We have now divided lines 424-444 into two paragraphs which include how our results were similar to previous Australian elimination campaigns. Besides the extensive efforts undertaken to engage the community, there were no hurdles overcome to make our eradication campaign successful.

Lines 379-387: “Similar techniques that targeted larval habitat were first utilized to control Ae. aegypti during the construction of the Panama Canal (47) and later when the species was eliminated from 23 countries in the Americas (48, 49). However, lessons learnt since these historical campaigns suggest traditional methods may not be sustainable for larger, modern towns and cities, where introductions will vary both spatially (heterogeneous distributions resulting from low dispersal behaviours) and temporally (drought-resistant eggs) (50, 51). Unfortunately, the current invasions of Ae. aegypti through California and Ae. albopictus through Europe are examples where undetected introductions were able to escape modern control methods (52, 53).”

**Line 412: References for these**

We have now included references for these regulatory documents:

Lines 469-474: “Queensland Health has the overall responsibility under the Public Health Act 2005 (22) for the surveillance and control of communicable diseases in Queensland, including exotic mosquito-borne diseases such as dengue fever. Provisions within Chapter 2 of the Public Health Act 2005 (22) provide local governments with the statutory support and powers to undertake mosquito surveillance and control activities (via insecticide treatment) and to prevent and control public health risks in relation to mosquitoes within residents’ premises.”

**Line 427: Suggest replacing with a more precise word such as larval habitat.**

We have to respectfully disagree with the use of larval habitat here. It is not always larval habitat that results in the presence of mosquitoes on a premises. We have instead replaced breeding with the term “presence”.

Lines 484-486: “Thus, a central element of urban mosquito control is to raise awareness about the community’s role to adopt behaviours that eliminate mosquito presence at home and in the workplace.”

**Reviewer 2:**

In Email Comments:

**Methods:**

**Needs summary of control activities**

We have provided a summary of control activities in Table 2. Line 275

**Results:**

**Summarize control activities**

Text describing control activities has been included in the results.

Lines 273-276: “Control activities included treating wet larval habitat sources such as bromeliad axils (503 total) and rainwater tanks (9 total) with S-methoprene pellets and briquettes, respectively (Table 2). Eight rainwater tanks were sealed, and 17 rubbish-containers removed (Table 2).”

Lines 320-322: “Control activities for the second suppression program (2015-2016) included treating 40 containers and one tank with S-methoprene pellets and briquettes, respectively, with four IRS treatments, 24 non-compliant tanks sealed (Fig 4), and 44 rubbish containers removed (Table 2)”

**Summary and General Comments:**

**This ms describes vector control and surveillance activities in a small regional town near Brisbane Queensland Australia,. In recent years there has been increased concern regarding reinvasion of the dengue vector Aedes aegypti into Brisbane, where this mosquito once caused large epidemics of the virus. You could argue that the ms is rather trivial, the simple elimination of Ae. aegypti from a country Australian town. Afterall, it has been done before, most recently published accounts by Whelan et al. for incursions into Tennant Creek in the NT. That said, the ms describes operational use of some novel surveillance tools and genetic analysis that will be of interest to readers.**

In our manuscript we show how a locally run approach, without the extensive funding thrown at past elimination campaigns, can achieve elimination when key larval habitat is targeted in low rainfall areas. We hope that is not just the novel tools and analysis that draw readers attention, but our low-cost approach and the extensive time and effort that has gone into documenting the disappearance of an Ae. aegypti after four years of surveillance (including the community engagement strategy, risk management strategy, control strategies, surveillance and mapping) will be an important contribution to the publicly accessible PLOS NTD literature, especially in developing countries where resources are poor.

**‘A few suggestions to improve the ms.**

**•	The ms could be shortened. The conclusions are just a repeat if what was said in the discussion. If you want to use it, use dot point of key messages.**

We thank the reader for the suggestion to decrease the length of our manuscript, but one of the reasons we have chosen PLOS NTD is that there is no word length on articles. This manuscript does not focus on simply a genetic analysis or novel surveillance activities, but the holistic approach to coordinating multiple stakeholders towards the goal of eliminating a population of Ae. aegypti. Considering reviewer 1’s request to provide more information, we think it is necessary for the manuscript to document the extensive efforts that have gone into the final result.

**•	The ms really needs summary table of control activities by year: no. premises surveyed, no. +ve, no containers treated or removed;**

We thank the reviewer for the suggestion to include a summary of control activities. We have now included this information in Table 2, including premises where treatment occurred. The number of premises surveyed is already provided in Table 5.

**•	The 2011 survey: was that organised by Pipi Mottram of QH? If so, she should be included as an author.**

Although Pipi was one of many involved in the original surveys, that was her total contribution to the outcome of this manuscript. We have now included her and Jarod Butler (who was responsible for the original report to the Bundaberg Regional Council suggesting the population be eliminated) in the Acknowledgements.

**•	Please cite relevant key used to ID captured mosquitoes, for both opvitraps and BG surveys.**

We have cited the key used to ID captured mosquitoes.

Lines 191-193: “Traps were serviced fortnightly, eggs counted, and larvae reared to fourth instar for identification to species via microscopy and key (26)”

Lines 221-222: “Adults were classified adults to species by microscope and key (26).”

Lines 227-228: “Concurrent BGS surveillance was undertaken by BRC (five weeks across five premises) in late summer. Larvae (reared to 4th instar) and adults were identified to species via microscope and key (26). “

**•	Line 167: I’d move ovitrap description to where they were first used (line 119).**

We have moved the description up where ovitraps were first mentioned and added a link to Appendix S1:

Lines 98-102: “One oviposition trap (ovitrap) containing aged rainwater, a lucerne pellet and an oviposition substrate (typically a red material or water-proof sandpaper (80 grit) Appendix S1; 24) and BG Sentinel trap (BGS; Biogents GmbH, Regensburg, Germany) (25) were set at the original positive premise, the surrounding properties and other high risk locations (caravan park, ambulance station, and council depot).”

**•	Ln 181: Please provide RWT mesh size, according to statute.**

We have included the size and the reference to this regulation in the text:

Lines 207-210: “During this second *Ae. aegypti* suppression program, all non-compliant rainwater tanks as defined by regulatory standards (e.g. mesh size apertures no greater than 1 mm to prevent entry or egress of mosquitoes) (23) were sealed with new mesh screens or silicone in rust-related holes.”

**•	Ln 232: “Mosquitoes were present…” all species? or just aegypti?**

This is referring to larvae of all species. I have included a reference to the species table in the Appendix:

Lines 260-261: “Mosquito larvae (Appendix S1-Table 4) were present in approximately 40% and 11% of all premises and wet containers, respectively (Table 1).”

**•	Ln 361: your genetic analysis should tell you if population is relict or recent introduction.**

With the resolution used in the genetic analysis applied here, it is not always possible to know if a population is newly established or has moved through a recent genetic bottleneck. We did not identify first generation immigrants within the analysis, which would indicate if individuals were not born in the population sampled. We have included a method for this analysis

Methods:

Lines 175-178: “To identify first generation immigrants, we used GeneClass2 (36) with a Bayesian method (37) for detecting first-generation immigrants with the resampling algorithm of Paetkau et al. (38) with 10000 simulated individuals, α = 0.01 and the L_home likelihood computation.”

Results:

Lines 293-394 “The inbreeding coefficient for the Gin Gin sample was moderate, but significant (Table 3) and no first generation immigrants were detected.”

Discussion:

Lines 432-433: “The resolution of the analysis did not establish whether this population was newly founded in 2011 or a relict population, but no first-generation immigrants were identified.”

**In Text Comments:**

**needs summary table of control activities:**

**no. premises surveyed, no. +ve, no containers treated or removed.**

We have provided a summary of control activities in Table 2.

**Line 1: The 2011 survey: was that organised by Pipi Mottram? If so she should be included as an author.**

Although Pipi was one of many involved in the original surveys, that was her total contribution to the outcome of this manuscript. We have now included her and Jarod Butler (who was responsible for the original report to the Bundaberg Regional Council suggesting the population be eliminated) in the Acknowledgements.

**Line 44: I'd say then a subsequent decline in the vector from SE Qld.**

We thank the reviewer for the suggestion. We have included a new sentence:

Lines 45-47: “During the mid-1900s, the Ae. aegypti distribution subsequently declined into central and northern Queensland, where temperature and rainfall conditions are favourable for the species (5,8).”

**Line 118: relevant key used**

We have cited the key used to ID captured mosquitoes.

Lines 191-193: “Traps were serviced fortnightly, eggs counted, and larvae reared to fourth instar for identification to species via microscopy and key (26)”

Lines 221-222: “Adults were classified adults to species by microscope and key (26).”

Lines 227-228: “Concurrent BGS surveillance was undertaken by BRC (five weeks across five premises) in late summer. Larvae (reared to 4th instar) and adults were identified to species via microscope and key (26). “

**Line 120: how ovitraps processed. you have other aedes. how distinguised**

We have included further detail on how ovitraps were processed earlier in the section:

Lines 98-104: “One oviposition trap (ovitrap) containing aged rainwater, a lucerne pellet and an oviposition substrate (typically a red material or water-proof sandpaper (80 grit) Appendix S1; 24) and BG Sentinel trap (BGS; Biogents GmbH, Regensburg, Germany) (25) were set at the original positive premise, the surrounding properties and other high risk locations (caravan park, ambulance station, and council depot). Ovitraps were collected weekly between the 4th and 11th of May and BGS traps between the 4th of May and 25th of October 2011. Eggs from ovitraps reared to fourth instar larvae for identification to species via microscopy and key (26).”

**Line 171: move this to describe ovitraps where queried earlier**

We have moved this to earlier in the section. Please see above.

**Line 181: please provide, according to statute**

We have included the size and the reference to this regulation in the text:

Lines 207-210: “During this second *Ae. aegypti* suppression program, all non-compliant rainwater tanks as defined by regulatory standards (e.g. mesh size apertures no greater than 1 mm to prevent entry or egress of mosquitoes) (23) were sealed with new mesh screens or silicone in rust-related holes.”

**Line 230: detected were they treated? if so what with?**

We have included the treatment results:

Lines 273-276: “Control activities included treating wet larval habitat sources such as bromeliad axils (503 total) and rainwater tanks (9) with with S-methoprene pellets and briquettes, respectively (Table 2). Eight rainwater tanks were sealed, and 17 rubbish-containers removed (Table 2).”

**Line 231: all species? or just aegypti?**

This is referring to larvae of all species. I have included a reference to the species table in the Appendix:

Lines 260-261: “Mosquito larvae (Appendix S1 Table 4) were present in approximately 40% and 11% of all premises and wet containers, respectively (Table 1).”

**Line 275: one**

We have included one in the sentence:
Lines 317-318: “Six positive premises were detected in 2013-2014 (five from larval surveys and from one GAT at a separate premises, across five residential blocks in north-eastern Gin Gin (Fig 3).”

**Line 344: need summary of control activities**

Text describing control activities has been included in the results.

Lines 273-276: “Control activities included treating wet larval habitat sources such as bromeliad axils (503 total) and rainwater tanks (9 total) with S-methoprene pellets and briquettes, respectively (Table 2). Eight rainwater tanks were sealed, and 17 rubbish-containers removed (Table 2).”

Lines 320-322: “Control activities for the second suppression program (2015-2016) included treating 40 containers and one tank with S-methoprene pellets and briquettes, respectively, with four IRS treatments, 24 non-compliant tanks sealed (Fig 4), and 44 rubbish containers removed (Table 2)”

**Line 361: your genetic analysis should tell you**

From above:

With the resolution used in the genetic analysis applied here, it is not always possible to know if a population is newly established or has moved through a recent genetic bottleneck. We did not identify first generation immigrants within the analysis, which would indicate if individuals were not born in the population sampled. We have included a method for this analysis

Methods:

Lines 175-178: “To identify first generation immigrants, we used GeneClass2 (36) with a Bayesian method (37) for detecting first-generation immigrants with the resampling algorithm of Paetkau et al. (38) with 10000 simulated individuals, α = 0.01 and the L_home likelihood computation.”

Results:

Lines 293-394 “The inbreeding coefficient for the Gin Gin sample was moderate, but significant (Table 3) and no first generation immigrants were detected.”

Discussion:

Lines 432-433: “The resolution of the analysis did not establish whether this population was newly founded in 2011 or a relict population, but no first-generation immigrants were identified.”

**Line 396: human**

This has been removed in the current version of the manuscript.

**Line 450: this seems redundant a shorter dot point style of key factors woukd be better**

We have included a larger conclusion as this manuscript represents an important summary of a considerable amount of work involving a number of stakeholders with interests in this area. It is important that we summarize the outcomes of the control programs and research undertaken in Gin Gin as well as expanding these implications to the greater implications at the state and national level regarding invasive mosquito species. With the ever increasing detection of invasive species at Australian first ports, it is only a matter time until we see a major establishment. We believe the large number of outcomes of this manuscript need to be summarized in a way that non-scientists can take away the implications of the work and without resorting to dot points.

**References: Capitalisation**

We thank the reviewer for noticing the error in the references. We have corrected capitalisation in this section.
