# Supplementary material for: Extensive public health initiatives drive the elimination of Aedes aegypti (Diptera, Culicidae) from a town in regional Queensland: A case study from Gin Gin, Australia
Source: PLoS Negl Trop Dis. 2022 Apr 8;16(4):e0010243. doi: 10.1371/journal.pntd.0010243 (PMC9020727; doi:10.1371/journal.pntd.0010243)
Supplement: Attachment — Submitted filename: ResponseToReviewers02.docx [file pntd.0010243.s009.docx]

Dear PLOS NTD editorial staff and reviewers, below we have provided responses to reviewer #2’s comment from the email that was sent from PLOS NTD, with line numbers corresponding to the newly revised manuscript. We hope these responses provide enough detail to assist you with your further review of our manuscript.

**Reviewer 2:**

**In Email Comments addressing the use of the term “cost”:**

**Reviewer #2: Sorry I missed this point on 1st review, but cost is critical.**

**In the Author summary you state: “Here we document the successful elimination of a population of Ae. aegypti from Gin 35 Gin, Australia, through a low-cost but extensive public health initiative”**

**Then in the discussion line 388 you state “The logistical resources and costs to scale our model to large urban areas would be significant (56)…”**

**But no costings of the initiative are given. So, can you provide a brief summary of the cost of the intervention, in labor and materials.**

**And how they compare to the NT (Tennant Creek, Groyte Is). elimination.**

**Author Summary:**

The Author Summary where we mention “cost” was only incorporated after the first review process. No where else is cost mentioned in the manuscript except to describe how hard it would be to scale our process over a large city. The resources that supported the elimination program documented in this manuscript were primarily in-kind labour support from local council, public health units and students, with some operating expenses such as juvenile hormones sourced from local councils. As there were a number of organisations involved, acting primarily through standard procedures, and no dedicated budget for the public health intervention, it will be impossible to estimate the actual cost of the elimination program. As such we have decided to remove the wording of “low-cost” and instead replace it with the term “locally resourced”.

Lines 34-37: “Here we document the elimination of a population of Ae. aegypti from Gin Gin, Australia, through an extensive and locally resourced public health initiative which included the use of community engagement, traditional mosquito control, population genetics and novel surveillance tools.”
